# (Phosphanyl)phosphaketenes as building blocks for novel phosphorus heterocycles†
†Electronic supplementary information (ESI) available: Characterization data, crystallographic data and Cartesian coordinates of DFT optimized structures. CCDC 1432815(**4**), 1432814 (**5**), 1528506 (**6**), 1432817 (**8**), 1528508 (**10**) and 1528507 (**10′**). For ESI and crystallographic data in CIF or other electronic format see DOI: 10.1039/c7sc00300e



**DOI:** 10.1039/c7sc00300e

**Published:** 2017-03-08

**Authors:** Max M. Hansmann, David A. Ruiz, Liu (Leo) Liu, Rodolphe Jazzar, Guy Bertrand

**Affiliations:** a UCSD-CNRS Joint Research Chemistry Laboratory (UMI 3555) , Department of Chemistry , University of California San Diego , La Jolla , CA 92093-0343 , USA . Email: guybertrand@ucsd.edu

## Abstract

Although BH_3_ simply coordinates the endocyclic P of (phospholidino)phosphaketene **1^Dipp^**, the bulkier B(C_6_F_5_)_3_ gives rise to a zwitterionic diphosphirenium, which is a novel type of 2π-electron aromatic system as shown by the calculated NICS values. While the reaction of **1^Dipp^** with Na[PCO(dioxane)_*x*_] is unselective, the same reaction with the sterically bulky (phospholidino)phosphaketene 1^Ar**^ [Ar** = 2,6-bis[di(4-*tert*-butylphenyl)methyl]-4-methylphenyl selectively affords a sodium bridged dimer containing a hitherto unknown λ^3^,λ^5^,λ^3^-triphosphete core. The latter formally results from “P^–^” addition to a 1,3-P/C-dipole. Similarly, adamantyl isonitrile adds to **1^Dipp^** giving a 4-membered phosphacycle. In contrast to **1**, the phosphaketene derived from the electrophilic diazaphospholidine-4,5-dione is unstable and reacts with a second molecule of Na[PCO(dioxane)_*x*_] to afford a 1,3,4-oxadiphospholonide derivative.

## Abstract 2

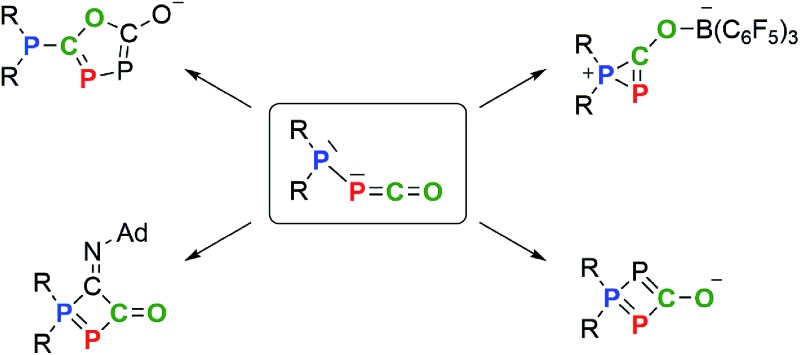
Although BH_3_ simply coordinates the endocyclic P of (phospholidino)phosphaketene **1^Dipp^**, the bulkier B(C_6_F_5_)_3_ gives rise to a zwitterionic diphosphirenium, which is a novel type of 2π-electron aromatic system as shown by the calculated NICS values.

## Introduction

**Scheme 1:**
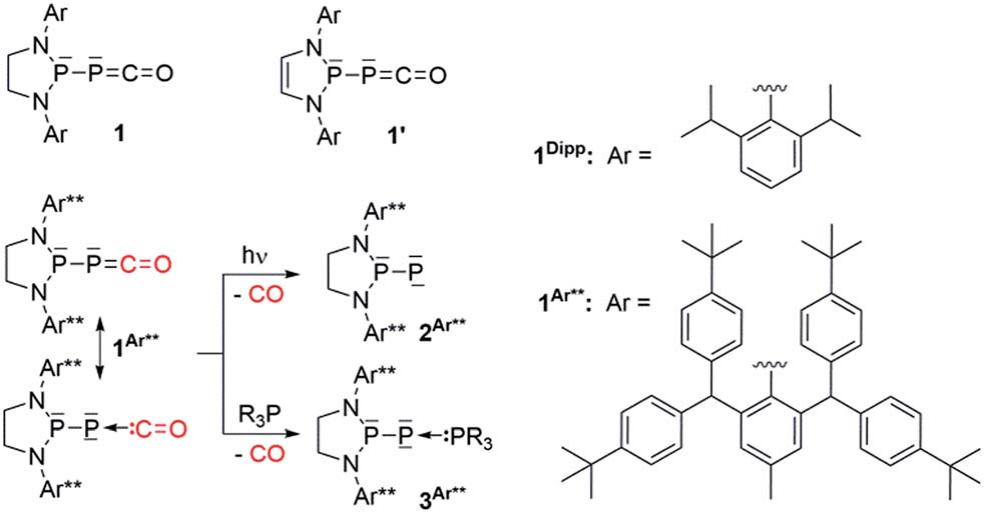
Recently reported (phosphino)phosphaketenes **1** (ref. 10) and **1′**,^11^ elimination and substitution of CO leading to the stable phosphinidene 2^Ar**^ and adducts 3^Ar**^, respectively.

Compared to the well-known isocyanates [R–N=C=O], the chemistry of their heavier homologues, namely phosphaketenes [R–P=C=O], has been largely unexplored. This is presumably the result of limited synthetic access and poor stability of their alkyl and aryl substituted derivatives.^1^ Indeed, pioneering work by Appel *et al.* showed that although the very bulky [C_6_H_3_(^*t*^Bu)_3_]–P=C=O can be isolated at room temperature, ^*t*^Bu-PCO dimerizes above –60 °C.^2^ However, the recent discovery of efficient preparation^3^ of phosphaethynolate salts (PCO^–^M^+^)^4^ has allowed access to group 14- (Si,^5^ Sn,^6^ Ge,^7^ Pb^5^) and transition metal- (Re, Au, Co, W)^8^ substituted phosphaketenes. In addition, the reaction of chlorodiaza-phospholidines and -phospholines^9^ with Na[PCO(dioxane)_*x*_] has allowed for the isolation of (phosphino)phosphaketenes **1** ^10^ and **1′**,^11^ respectively (Scheme 1). Although the phosphaketene moiety of **1′** reacts with the unsaturated backbone to give various rearrangement products,^11^ (phospholidino)-phosphaketene **1^Dipp^** is thermally very stable (heating a toluene solution of **1^Dipp^** overnight at 80 °C does not lead to decomposition or any rearrangement products), which allows for studying the reactivity of the [P]–PCO moiety. We have already reported that elimination of CO occurred under irradiation of 1^Ar**^, affording the corresponding room temperature stable phosphinidene 2^Ar**^,^10*a*^ while addition of phosphines to 1^Ar**^ and **1^Dipp^** leads to adducts **3**.^12^ In both of these reactions, the phosphaketene group acts as a phosphinidene-carbonyl adduct. This behavior is reminiscent of the chemistry of transition metal carbonyl complexes, and it is noteworthy that before our work the chemistry of main group carbonyl compounds^13^ was essentially limited to boranes,^14^ polyboranes^15^ and carbenes.^16^ Herein we report that the P–PCO scaffold can also react without loss of CO to give access to a variety of hitherto unknown phosphorus heterocycles.

## Results and discussion

**Scheme 2:**
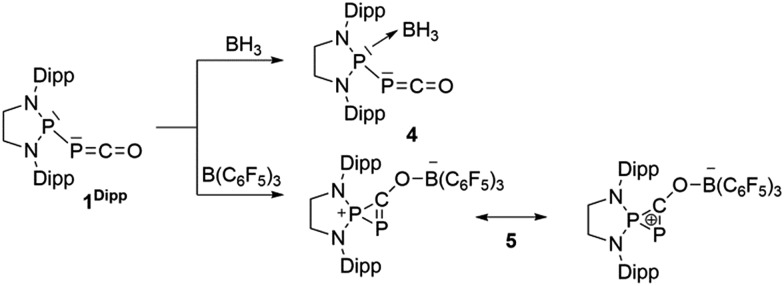
Reactivity of **1^Dipp^** with boranes.

**Fig. 1:**
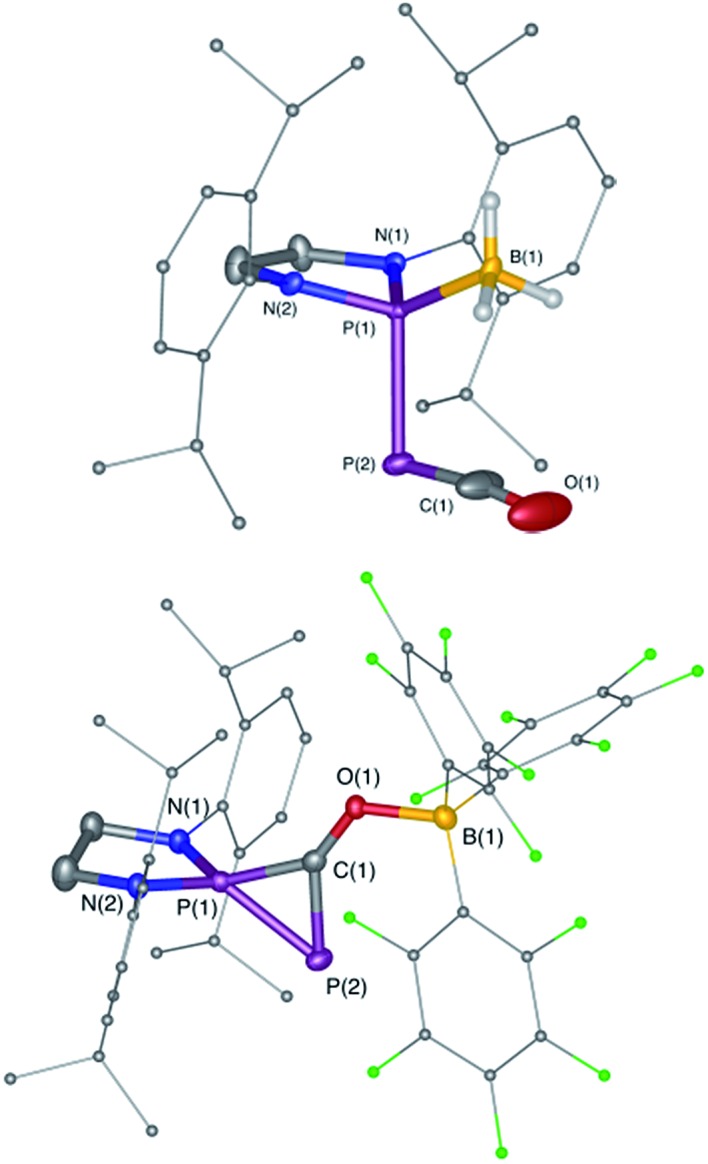
Solid-state structures of **4** (top) and **5** (bottom). Hydrogen atoms are omitted for clarity. Ellipsoids shown at 50% probability. Selected bond parameters for **4** in [Å] and [°]: P1–P2 2.2657(16), P2–C1 1.533(6), C1–O1 1.165(8), P1–B1 1.906(6), P1–P2–C1 96.8(2), P2–C1–O1 171.9(6), B1–P1–P2 113.0(2); and **5**: P1–P2 2.0804(14), P2–C1 1.748(4), P1–C1 1.727(4), C1–O1 1.289(4), O1–B1 1.560(5), P2–P1–C1 53.67(13), P1–C1–P2 73.55(15), P1–C1–O1 143.7(3), C1–O1–B1 125.0(3).

**Fig. 2:**
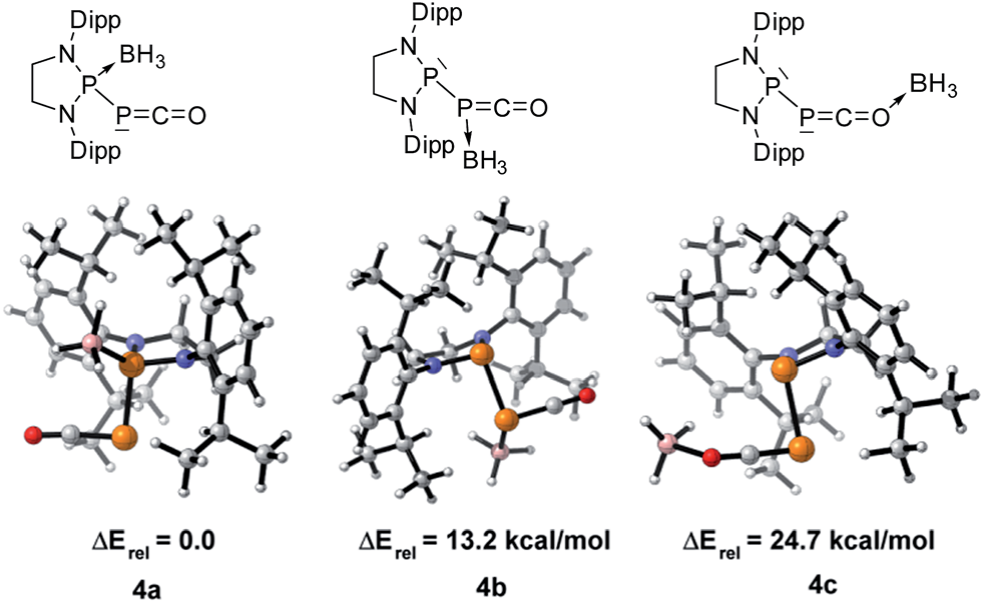
Optimized structures of three BH_3_ adduct isomers and their relative electronic energies at the B3LYP/def2-TZVP level of theory.

We started our investigation by studying the electrophilic activation of the [P]–PCO moiety of **1^Dipp^**, with the aim of triggering the loss of carbon monoxide. We chose two different boron-derived Lewis acids. Upon addition of excess BH_3_, simple coordination to the endocyclic P center occurs giving **4**, as shown by the ^31^P NMR spectrum [–226 ppm (d), +131 ppm (br. d), *J*_PP_ = 295 Hz] and by a single crystal X-ray diffraction study (Scheme 2; Fig. 1, top). To understand the regioselectivity of the reaction, three BH_3_ adduct isomers were optimized at the B3LYP-D3BJ/def2-TZVP level of theory (Fig. 2). The results show that the observed product **4** is more thermodynamically stable than **4b** and **4c** by +13.2 and +24.7 kcal mol^–1^ (gas-phase electronic energies), respectively. Moreover, since the absolute coefficient of the HOMO of **1^Dipp^** at the endocyclic P (0.42) is much larger than those at the phosphorus of PCO (0.32) and at O (0.11), **4** is also the kinetic product of the reaction.

**Scheme 3:**
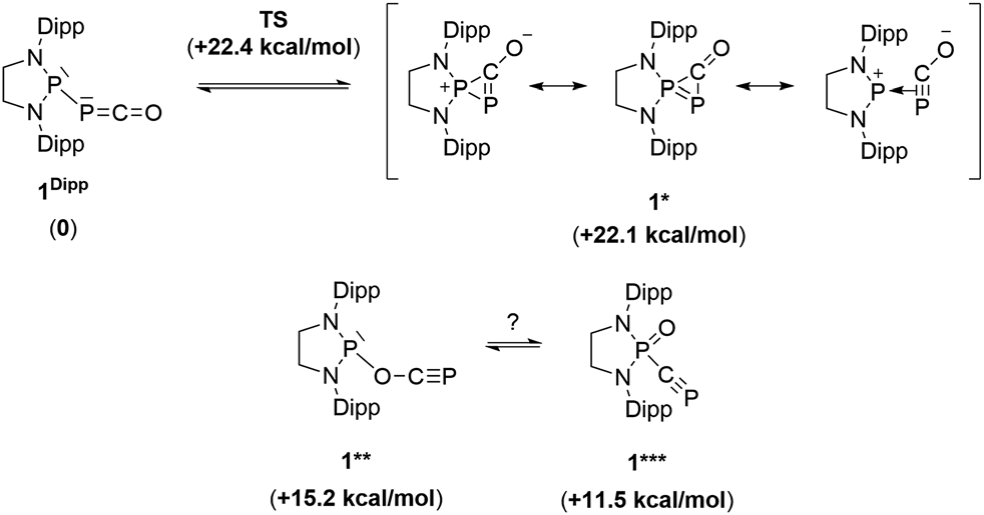
Possible isomers of the P–PCO moiety and their relative electronic energies (in brackets) compared to **1^Dipp^** (B3LYP-D3BJ/cc-pVDZ level of theory).

Due to the steric environment around the endocyclic P atom, we wondered whether a larger borane would react at a different site (Scheme 2). Mixing **1^Dipp^** and B(C_6_F_5_)_3_ resulted in a new product as observed by ^31^P NMR spectroscopy with two sharp doublets at *δ* = +206 and –11 ppm (*J*_PP_ = 215 Hz). An X-ray diffraction study revealed the formation of the unusual zwitterionic diphosphirenium **5** (Fig. 1, bottom).^17^ The PP bond distance (2.0804(14) Å) becomes significantly shorter than in **1^Dipp^** (2.3782(8) Å)^10^ and is in the outer range for PP double bonds (1.985–2.050 Å).^18^ Concomitantly, the CO bond elongates from 1.170(3) Å in **1^Dipp^** to 1.289(4) Å in **5**. It is important to note that the computed nucleus independent chemical shift (NICS)^19^ values for the central three-membered ring are negative [NICS(0) = –17.33 and NICS(1) = –11.71 ppm], which suggests that the three-membered ring of **5** is a 2π-electron aromatic system. Mechanistically, the interaction of the borane with the oxygen atom induced a ring closure between the carbon ketene atom and the endocyclic P center. Alternatively, a reviewer suggested that the borane abstracts the PCO^–^ moiety to form a close ion contact-pair [P]^+^[PCO-BR_3_]^–^ followed by coordination of the phosphaalkyne to the electrophilic phosphorus center.^20^ However, DFT calculations indicate that the heterolytic cleavage of the P–P bond is energetically very costly. Moreover, a transition state in agreement with a concerted Lewis acid activation of **1** has been located using the small BF_3_ Lewis acid as a model (Fig. S1†). Interestingly, **5** can be regarded as 1*, the cyclic isomer of **1** trapped by a Lewis acid. DFT calculations predict an energy barrier of 22.4 kcal mol^–1^ for the endergonic interconversion of **1** into its cyclic isomer 1* (Δ*E* = 22.1 kcal mol^–1^) (Scheme 3). Note that the [P]–OCP (1**) and [P=O]–CP (1***) isomers are predicted to be 15.2 and 11.5 kcal mol^–1^, respectively, higher in energy than **1**.^21^

**Scheme 4:**
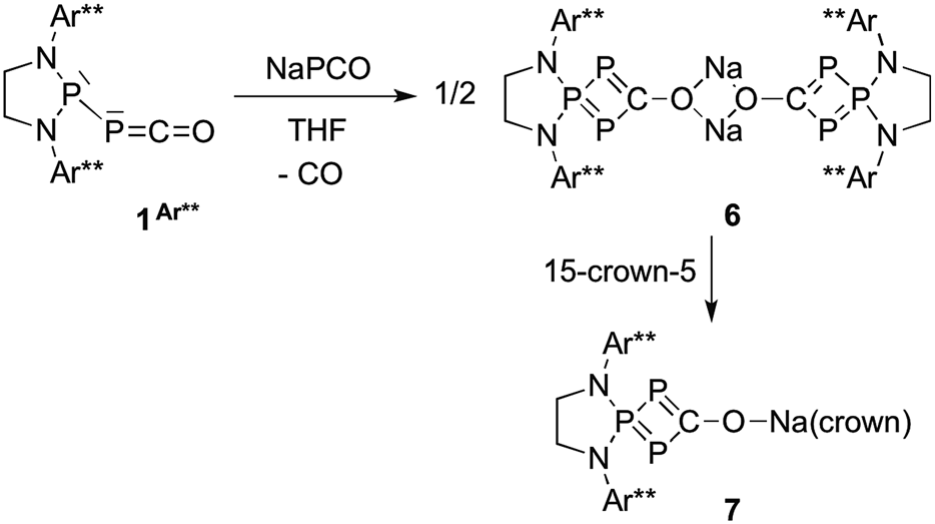
Synthesis of the 1,2,3-triphosphete **7** *via* a formal [1 + 3]-cycloaddition of P^–^ to the PPC unit of 1^Ar**^.

**Fig. 3:**
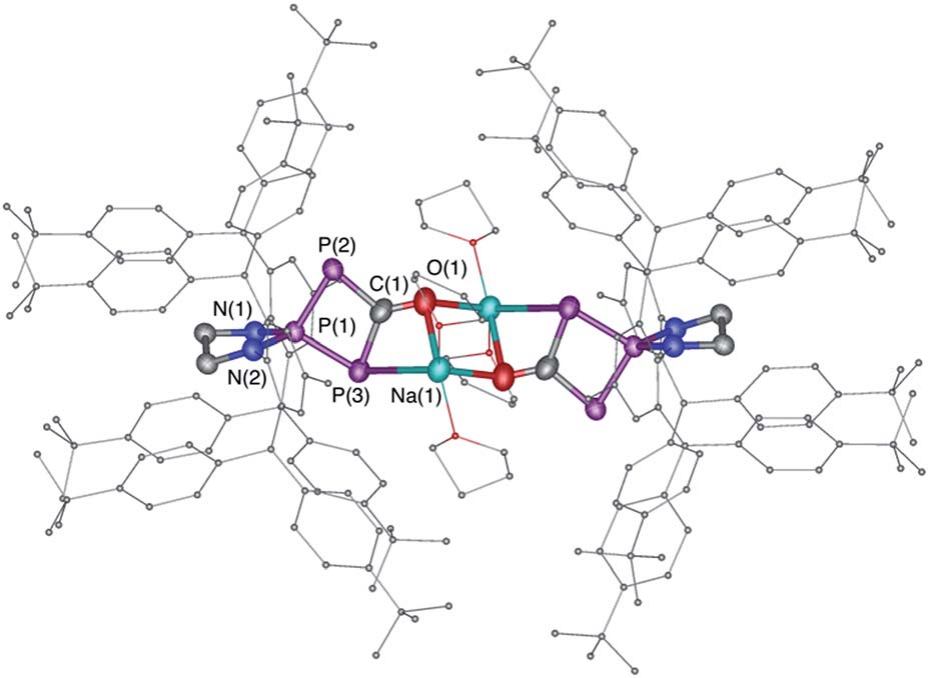
Solid-state structure of **6**. Hydrogen atoms are omitted for clarity. Ellipsoids shown at 50% probability. Selected bond parameters in [Å] and [°]: P1–P2 2.1213(15), P1–P3 2.1371(14), P2–C1 1.820(5), C1–P3 1.854(5), C1–O1 1.257(5), O1–Na1 2.305(4), P3–P1–P2 91.44(5), P1–P2–C1 78.76(15).

**Fig. 4:**
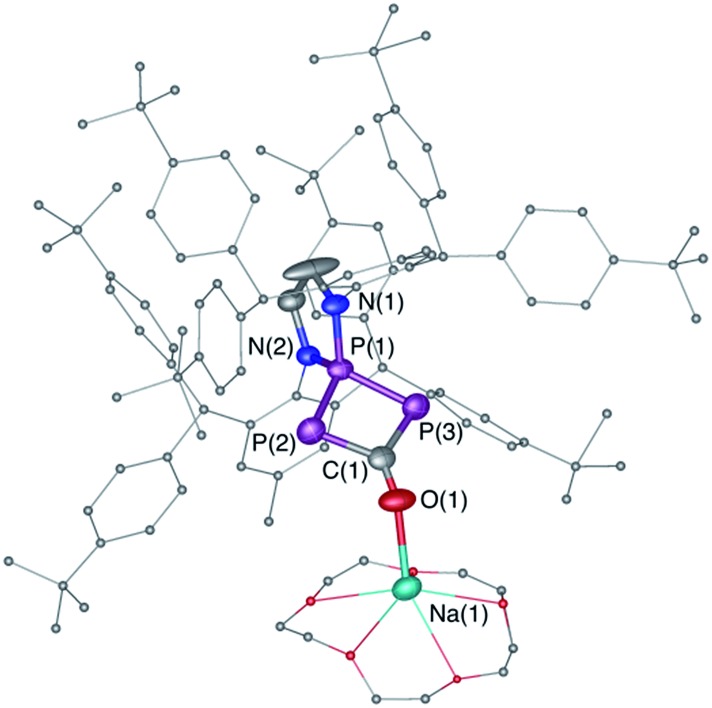
Solid-state structure of **7**. Hydrogen atoms are omitted for clarity. Structural data are not presented due to the low quality of the X-ray data.

While the reaction of **1^Dipp^** with Na[PCO(dioxane)_*x*_] is unselective, giving rise to several compounds, we observed that the same reaction with the sterically bulky (phosphino)phosphaketene 1^Ar**^, featuring 2,6-bis[di(4-*tert*-butylphenyl)methyl]-4-methylphenyl substituents,^22^ was highly selective. Independent of the excess Na[PCO(dioxane)_*x*_] used (or one equivalent), the ^31^P NMR spectrum showed the formation of a single product [+126.1 (d), +69.6 ppm (t), *J*_PP_ = 302 Hz]. An X-ray diffraction study revealed that it was the sodium bridged dimer **6** containing the hitherto unknown λ^3^,λ^5^,λ^3^-triphosphete core (Scheme 4, Fig. 3). Upon addition of 15-crown-5 to **6** a slight shift of the ^31^P NMR signals [+119.7 (d) and +78.8 ppm (t), *J*_PP_ = 310 Hz] was observed and the corresponding monomer **7** could be characterized by X-ray diffraction (Fig. 4).

The formation of the triphosphete scaffold formally results from a [1 + 3]-cycloaddition of “P^–^”^23^ (from NaPCO) to the PPC unit. Indeed, when the ^13^C labeled phosphaketene 1^Ar**^ was reacted with non-^13^C-labeled Na[PCO(dioxane)_*x*_], we observed an intense broad resonance^24^ at *δ* ^13^C = 250.2 ppm demonstrating that the PPCO moiety remains, at least to a large extent, in the final product. Mechanistically, DFT calculations indicate that the reaction involves an initial attack onto the carbon atom of the PCO moiety followed by cyclization with simultaneous loss of CO (Δ*G* ^‡^ = 16.1 kcal mol^–1^) (see Fig. S2 in ESI†). Aside from the formation of a novel type of phosphorus heterocycle, these results are interesting because they give important information on the synthesis of (phosphino)phosphaketenes **1**. Indeed, to prepare the latter, it is crucial to use only one equivalent of Na[PCO(dioxane)_*x*_] and toluene as the solvent in which Na[PCO(dioxane)_*x*_] is only poorly soluble. Otherwise, instead of **1**, heterocycles of type **6** are formed as the major product.

**Scheme 5:**
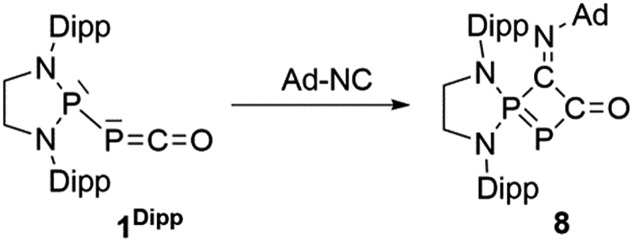
Synthesis of heterocycle **8** *via* a formal [1 + 3]-cycloaddition of adamantyl isonitrile to the PPC unit of 1^Ar**^.

**Fig. 5:**
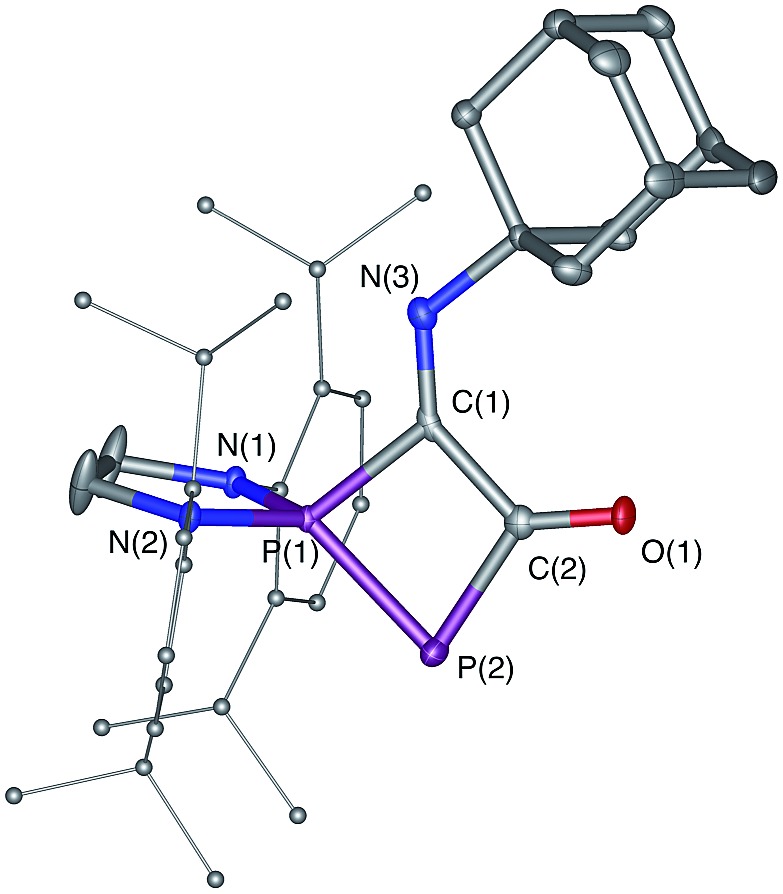
Solid-state structure of **8**. Hydrogen atoms are omitted for clarity. Ellipsoids shown at 50% probability. Selected bond lengths [Å] and angles [°]: P1–P2 2.1028(11), P1–C1 1.837(3), C1–C2 1.535(4), C2–P2 1.832(3), C2–O1 1.225(4), C1–N3 1.265(4), C2–P2–P1 77.96(11), P2–P1–C1 84.17(10).

Serendipitously, we also prepared another novel type of phosphorus heterocycle formally resulting from a [1 + 3]-cycloaddition. As isonitriles and carbon monoxide are isoelectronic, we were interested in the thermal substitution of the CO in phosphaketene **1^Dipp^** by an isonitrile, using our recently reported ligand exchange strategy.^12^ Surprisingly, the isonitrile does not add at the phosphorus center of PCO to displace CO, as previously observed with phosphines, but attacks at the carbon.^25^ This is followed by a cyclization involving the endocyclic P and the resulting heterocycle **8** was isolated in 85% yield (*δ* ^31^P 148.0 and 88.3 ppm, *J* = 370 Hz) (Scheme 5, Fig. 5).

**Fig. 6:**
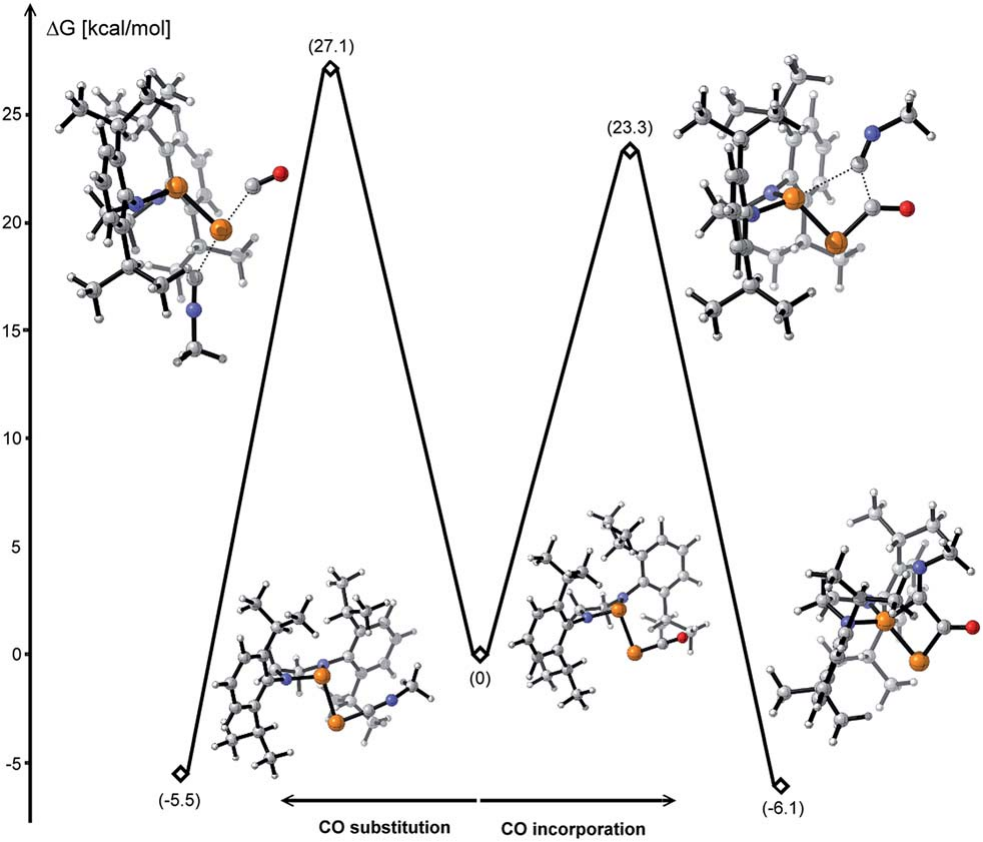
Energy profiles for the CO substitution and incorporation of an isonitrile to **1** calculated at the B3LYP-D3BJ/cc-pVDZ level of theory.

The formal insertion of an isonitrile giving **8** can be rationalized by a mechanism similar to that postulated for the insertion of P- leading to **6**. According to DFT calculations, this process is exergonic by 6.1 kcal mol^–1^ with an energy barrier of 23.3 kcal mol^–1^ (Fig. 6, right). Note that direct substitution of CO by the isonitrile is also exergonic by 5.5 kcal mol^–1^ but with a higher activation energy barrier (27.1 kcal mol^–1^) (Fig. 6, left).

**Scheme 6:**
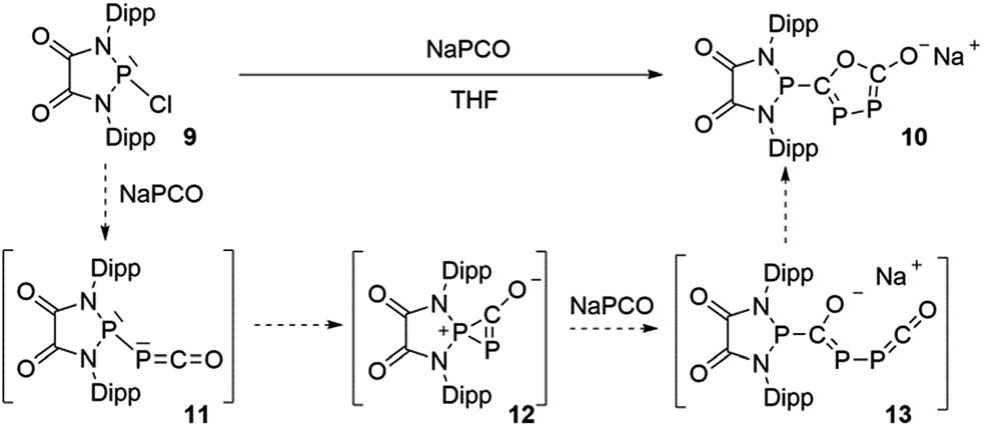
Attempted synthesis of (phopshino)phosphaketene **11** and the formation of heterocycle **10**.

**Fig. 7:**
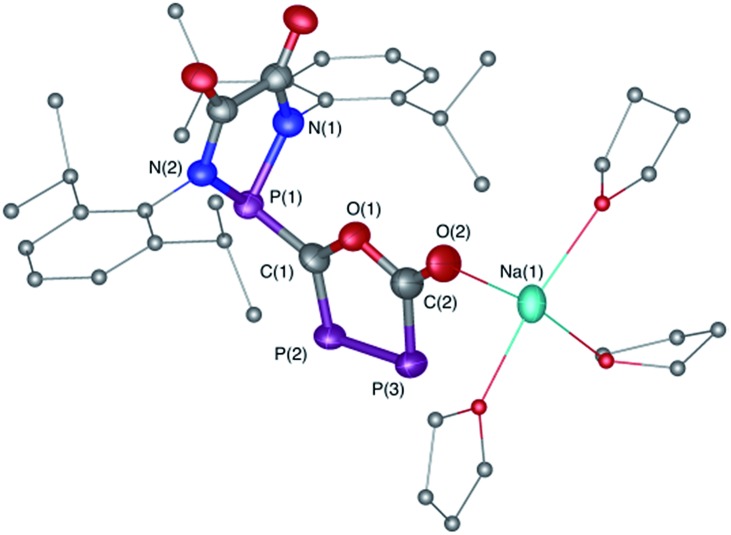
Solid-state structure of **10** crystalized from THF. Hydrogen atoms and THF molecules coordinated are omitted for clarity. Ellipsoids shown at 50% probability. Selected bond parameters for **10** in [Å] and [°]: P1–C1 1.759(5), C1–P2 1.696(5), P2–P3 2.096(3), P3–C2 1.795(6), C2–O1 1.405(6), C2–O2 1.231(7), O1–C1 1.389(6), O2–Na1 2.329(4), P1–C1–P2 118.3(3), C1–P2–P3 94.9(2), P2–P3–C2 93.5(2).

**Fig. 8:**
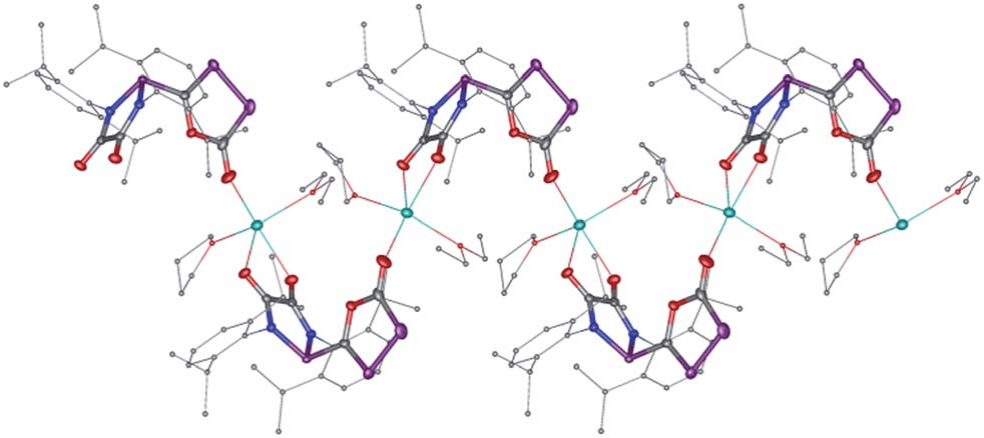
Stolid-state structure of **10** crystalized from THF/Et_2_O/CH_3_CN showing the polymeric chain nature. Ellipsoids shown at 50% probability.

The difficulty in synthesizing (phosphino)phosphaketenes is illustrated by our attempt to prepare **11** derived from the electrophilic diazaphospholidine-4,5-dione (Scheme 6). A single product was formed upon mixing **9** with NaPCO, but the ^31^P NMR spectrum revealed the presence of three different phosphorus nuclei [^31^P NMR *δ* = +323 (dd, *J* = 466 Hz, 282 Hz); +48 (d, *J* = 282 Hz); +45 (d, *J* = 466 Hz) ppm]. An X-ray diffraction study revealed the 1,3,4-oxadiphospholonide core **10** (Fig. 7), a type of heterocycle previously only observed by Grützmacher *et al.* in the reaction of NaPCO with tetraphenyl-cyclopentadienone.^26^ Interestingly, in the solid-state this compound features a linear polymeric network structure in which the sodium cation is bridging between the diketone moiety and the phosphorus heterocycle (Fig. 8).

Mechanistically, it seems reasonable to postulate the initially formed (phosphino)phosphaketene **11** spontaneously rearranges into the spirocyclic zwitterionic derivative **12** which resembles the borane adduct **5**. Then, a second equivalent of NaPCO induces a ring opening giving **13**, which undergoes a ring closure leading to the observed product **10**.

## Conclusions

This work has shown that (phosphino)phosphaketenes are powerful building blocks in heterocyclic chemistry. In contrast to our recent reported CO substitution approach,^12^ this work demonstrates the feasibility of nucleophiles to add to carbon on the phosphaketene moiety. The endocyclic P center can either activate the phosphaketene by forming highly reactive diphosphirenium species or engage in ring closing reactions. Importantly the stability and chemical behaviour of these novel heterocycles is strongly dependent on the nature of the phosphino substituents.
