# Supplementary material for: (Phosphanyl)phosphaketenes as building blocks for novel phosphorus heterocycles
Source: Chem Sci. 2017 Mar 8;8(5):3720–5. doi: 10.1039/c7sc00300e (PMC5437511; doi:10.1039/c7sc00300e)
Supplement: Supplementary file 1 [file SC-008-C7SC00300E-s001.pdf]

Electronic Supplementary Material (ESI) for Chemical Science.
This journal is © The Royal Society of Chemistry 2017

Supplementary Information

for

(Phosphanyl)phosphaketenes as Building Blocks for Novel Phosphorus Heterocycles

Max M. Hansmann, David A. Ruiz, Liu (Leo) Liu, Rodolphe Jazzar and Guy Bertrand*

Table of Contents

1. *General Information* 1
2. **Synthesis and Characterization data** 2
3. **X-ray data** 15
4. **Computational part** 29
5. *References* 64

1. *General Information*

All manipulations were performed under an atmosphere of dry argon using standard Schlenk or dry box techniques. Solvents were dried by standard methods and distilled under argon. ^{1}H, ^{31}P, ^{19}F and ^{13}C NMR spectra were recorded on a Varian VX 500, Bruker 300 and Jeol 500 spectrometer at 25°C. NMR multiplicities are abbreviated as follows: *s* = singlet, *d* = doublet, *t* = triplet, *sept* = septet, *m* = multiplet, *br* = broad signal. Chemical shifts are given in ppm and coupling constants *J* are given in Hz. Single crystal X-ray diffraction data were collected on a Bruker Apex II-CCD detector using Mo-Kα radiation (λ = 0.71073 Å) or Cu-Kα radiation (λ = 1.54178 Å) at the UC San Diego Crystallography Facility. Mass spectra were performed at the UC San Diego Mass Spectrometry Laboratory. Melting points were measured with an electrothermal MEL-TEMP apparatus. **1**$^{\mathbf{Dipp}}$ and **1**$^{\mathbf{Ar**}}$ were synthesized according to literature procedures.[1]

2. Synthesis and Characterization data

Preparation of 4:

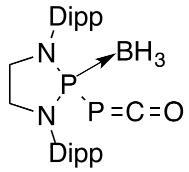

A mixture of $\mathbf{1}^{\mathbf{Dipp}}$ (30 mg, 0.064 mmol) and excess BH_3-SMe_2 was dissolved in pentane (2 mL). The solution stirred for 30 minutes and the resulting white powder was separated by filtration and washed with 2 × 2 mL of pentane. **4** was isolated as a white solid (22 mg, yield: 72 %). Single crystals were grown from a concentrated pentane solution. M.p.: 130 ºC (dec.). $^{31}P\{^{1}H\}$ NMR (pentane, 300 MHz): δ = 131.2 (br d, J_{PP} = 295 Hz), –226.1 (J_{PP} = 295 Hz). In solution the mixture bubbled continuously leading to decomposition, making meaningful interpretation of the ^{13}C and ^{1}H NMR spectra impossible. Numerous attempts towards recrystallization resulted in crystals of poor quality. However, a structure was successfully solved confirming the coordination of the borane onto the cyclic P atom.

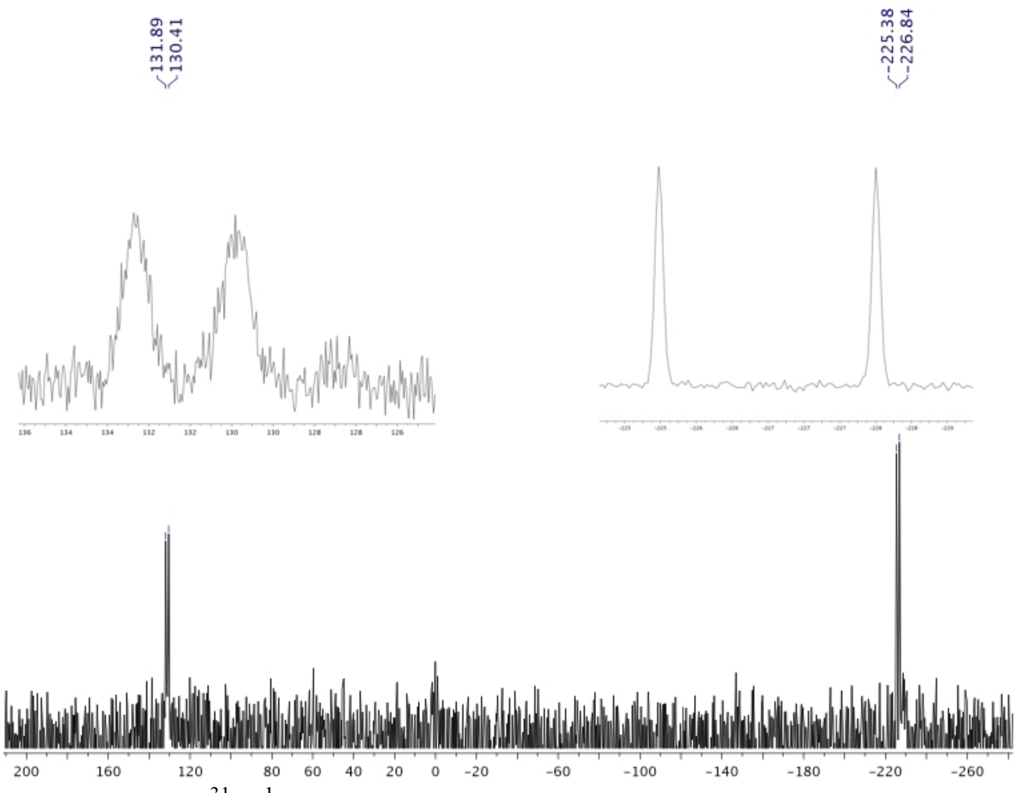

$^{31}P\{^{1}H\}$ NMR (pentane, 300 MHz, 298K) of **4**

Preparation of 5:

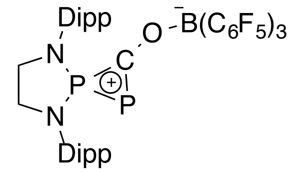

A mixture of **1**[Dipp] (30 mg, 0.064 mmol) and $B(C_6F_5)_3$ (33 mg, 0.064 mmol) was dissolved in pentane (1 mL). The solution stirred for 30 minutes and resulted in a white powder, which was separated from the solution and washed with 2 × 2 mL of pentane. **5** was isolated as a white solid (50 mg, yield: 80 %). Single crystals were grown form a concentrated pentane solution. M.p.: 152 ºC (dec.). **^{1}H NMR** (C_6D_6, 500 MHz, 298K): δ [ppm] = 7.21 (t, J = 7.5 Hz, 2H), 7.09 (d, J = 7.5 Hz, 1H), 7.00 (d, J = 7.5 Hz, 1H), 6.95 (d, J = 7.5 Hz, 2H), 3.48 (m, 2H), 3.23 (m, 2H), 3.13 (sept, J = 6.5 Hz, 2H), 3.08 (sept, J = 6.5 Hz, 2H), 1.33 (d, J = 6.5 Hz, 3H), 1.24 (dd, 9H), 1.03 (m, 12H); **^{13}C NMR** (C_6D_6, 125 MHz, 298K): δ [ppm] = 195.1 (d, J_{PC} = 97.8 Hz), 149.3, 148.4 (br d, J_{CF} = 245 Hz), 147.9, 140.6 (br d, J_{CF} = 250 Hz), 137.5 (br d, J_{CF} = 248 Hz), 130.6, 125.5, 125.0, 124.7, 55.8 (d, J_{PC} = 8.7 Hz), 49.0 (d, J_{PC} = 12.0 Hz), 29.5 (d, J_{PC} = 9.9 Hz), 26.0, 25.8, 25.4, 24.8, 23.9, 22.4; **^{31}P{^{1}H} NMR** (C_6D_6, 121 MHz, 298K) δ [ppm] = 205.8 (d, J_{PP} = 215.4 Hz, *P*PCO), –10.6 (d, J_{PP} = 215.4 Hz, P*P*CO); **^{19}F NMR** (C_6D_6, 283 MHz, 298K) δ [ppm] = –132.1 (br), -157.4 (t, J_{FF} = 22.0 Hz), –163.8 (t, J_{FF} = 22.0 Hz). **HR-MS**: m/z calculated for $[C_{45}H_{39}BF_{15}N_2O_2P_2]^-$ $(M\text{-}H)^-$ 996.2382; found 996.2378.

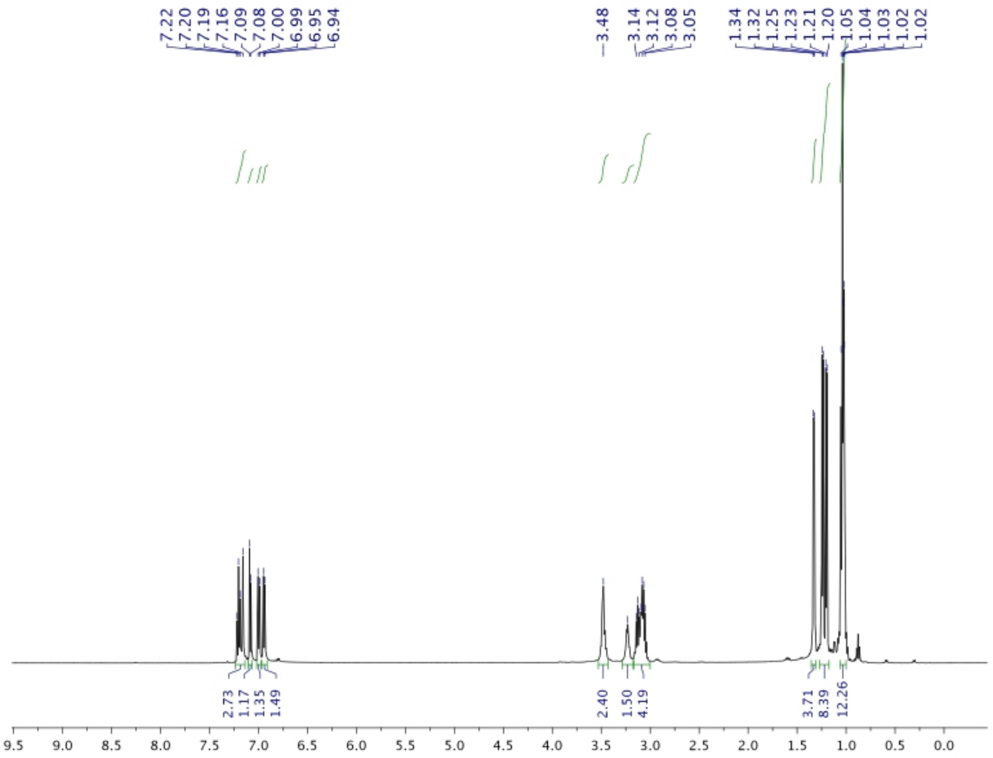

^{1}H NMR (C_6D_6, 500 MHz, 298K) of **5**

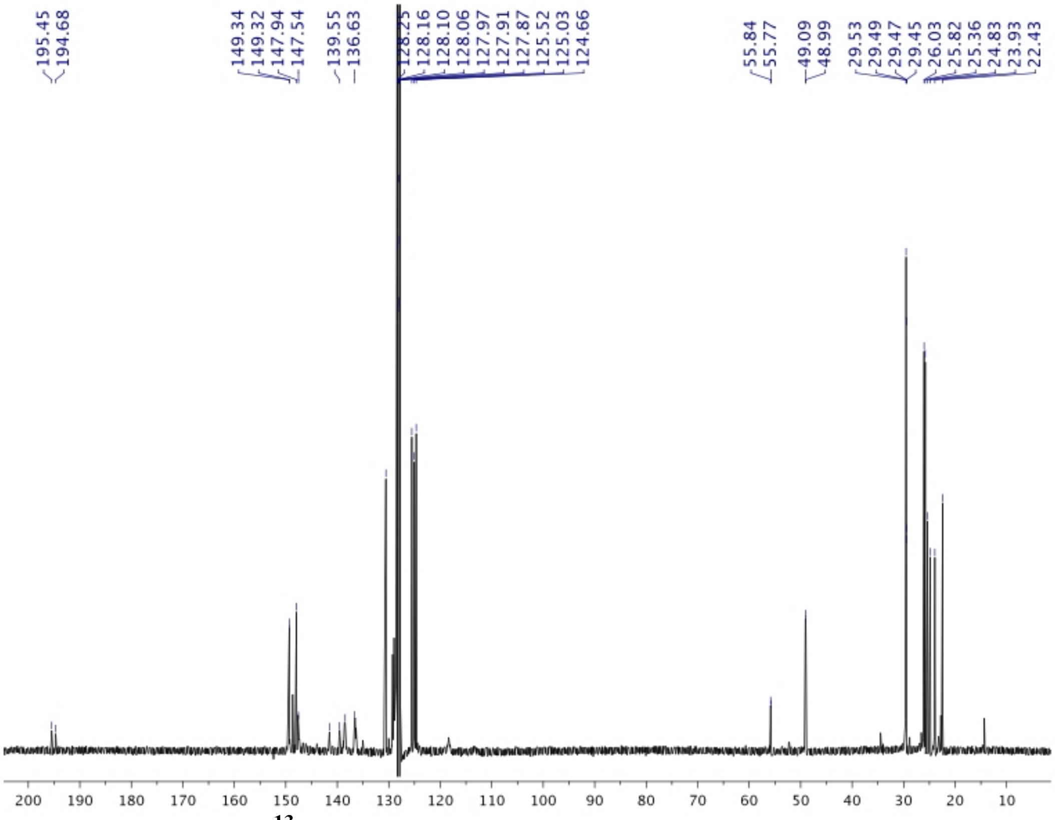

^{13}C NMR (C_6D_6, 125 MHz, 298K) of **5**

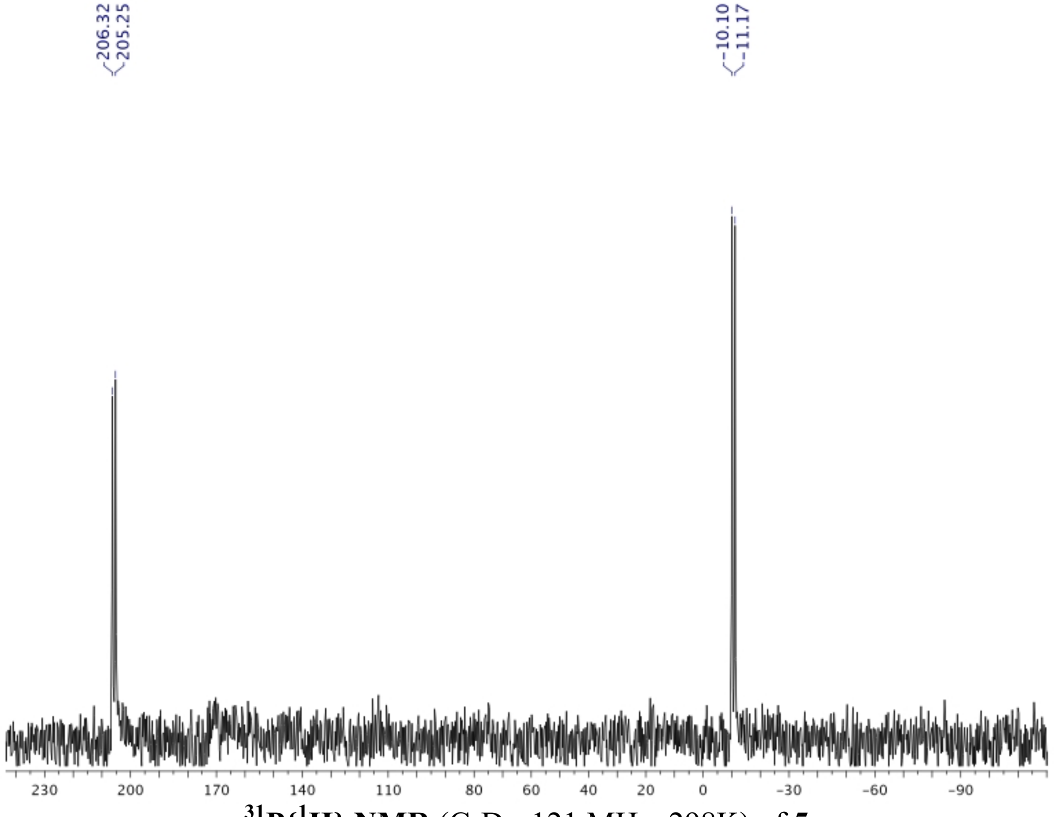

^{31}P{^{1}H} NMR (C_6D_6, 121 MHz, 298K) of **5**

Preparation of 6:

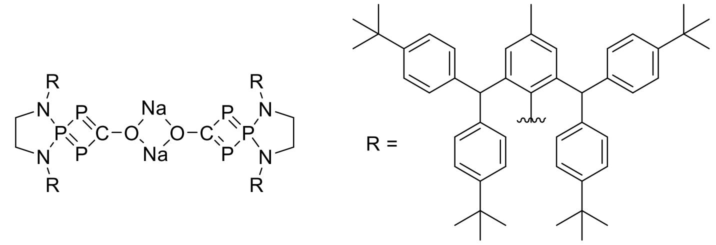

1^{Ar**} (80 mg, 0.06 mmol) and NaPCO(dioxane)$_{3.0}$ (60 mg, 0.17 mmol, 3.0 eq) were dissolved in THF (1 mL). The solution was stirred for 15 min, the solvent removed under reduced pressure and the remaining solid dissolved in toluene (2 mL). The suspension was filtered and the solvent removed under reduced pressure to give **6** as slightly yellow solid (62 mg, 73%). Even though this transformation is very selective, bulk samples of **6** could not be obtained free from impurities (hydrolysis of starting material).

^{1}H NMR (C_6D_6, 300 MHz, 298K): δ [ppm] = 7.74 (d, J = 8.3 Hz, 8H), 7.35-7.29 (m, 8H), 7.19-7.09 (m, 12H), 7.02 (d, J = 8.3 Hz, 8H), 3.64 (brs, coord. THF), 3.42 (s, 4H), 2.37-2.26 (m, 4H), 1.90 (s, 6H), 1.44 (brs, coord. THF), 1.24 (s, 36H), 1.19 (s, 36H); **^{13}C NMR** (C_6D_6, 125 MHz, 298K): δ [ppm] = 250.2 (m, P_3C-ring), 148.6, 148.5, 146.9, 144.2, 142.6, 138.9, 125.3, 131.0, 130.9, 129.8, 125.2, 125.0, 68.1, 67.3, 51.2, 49.2, 34.5, 34.4, 31.7, 31.7, 25.8; **^{31}P NMR** (C_6D_6, 121 MHz, 298K): δ [ppm] = + 126.1 (d, J = 302 Hz), + 69.6 (J = 298 Hz, 310 Hz). **HR-MS**: m/z calculated for $[C_{101}H_{122}BN_2OP_3]^-$ [Monomer; M-Na]$^-$1471.8770; found 1471.8755.

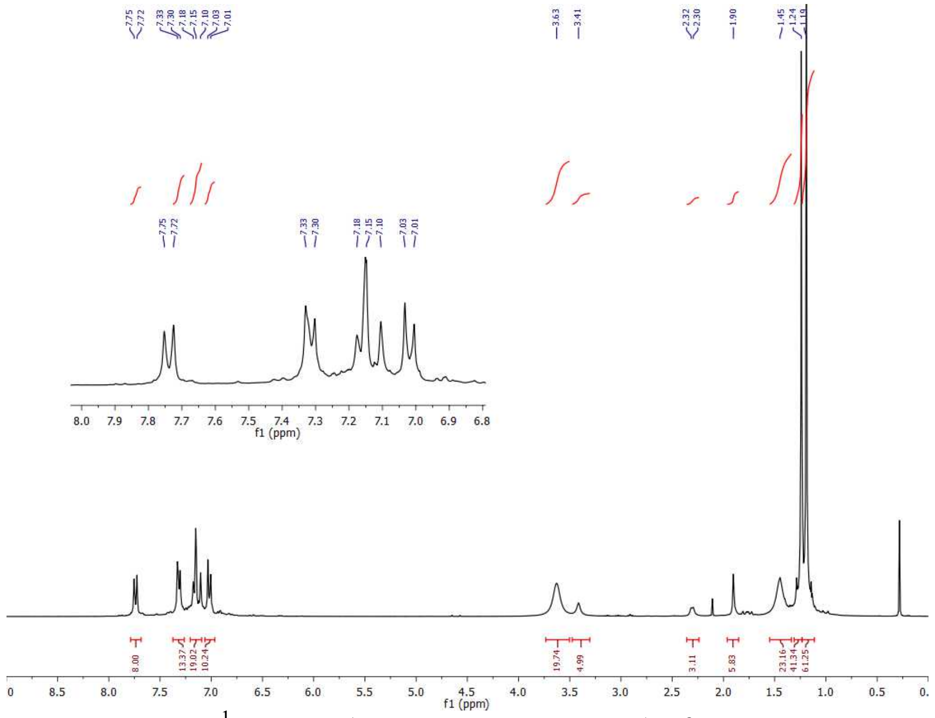

^{1}H NMR (C_6D_6, 300 MHz, 298K) of **6**

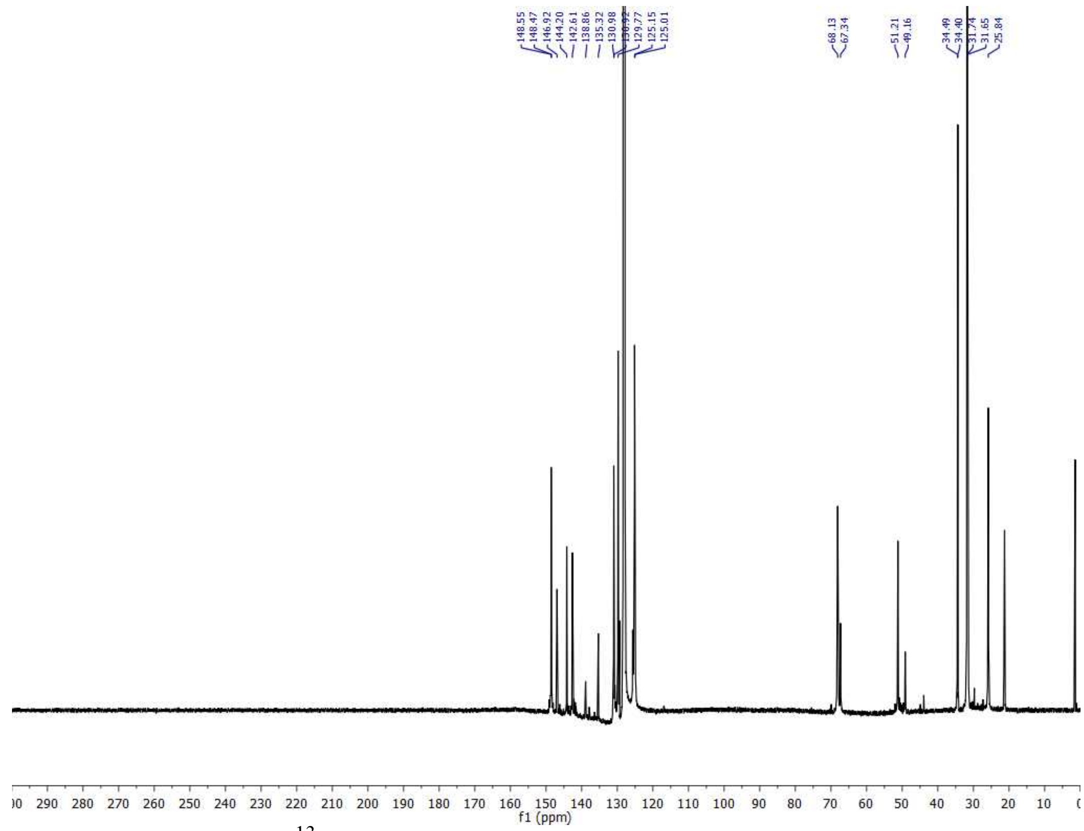

^{13}C NMR (C_6D_6, 125 MHz, 298K) of **6**

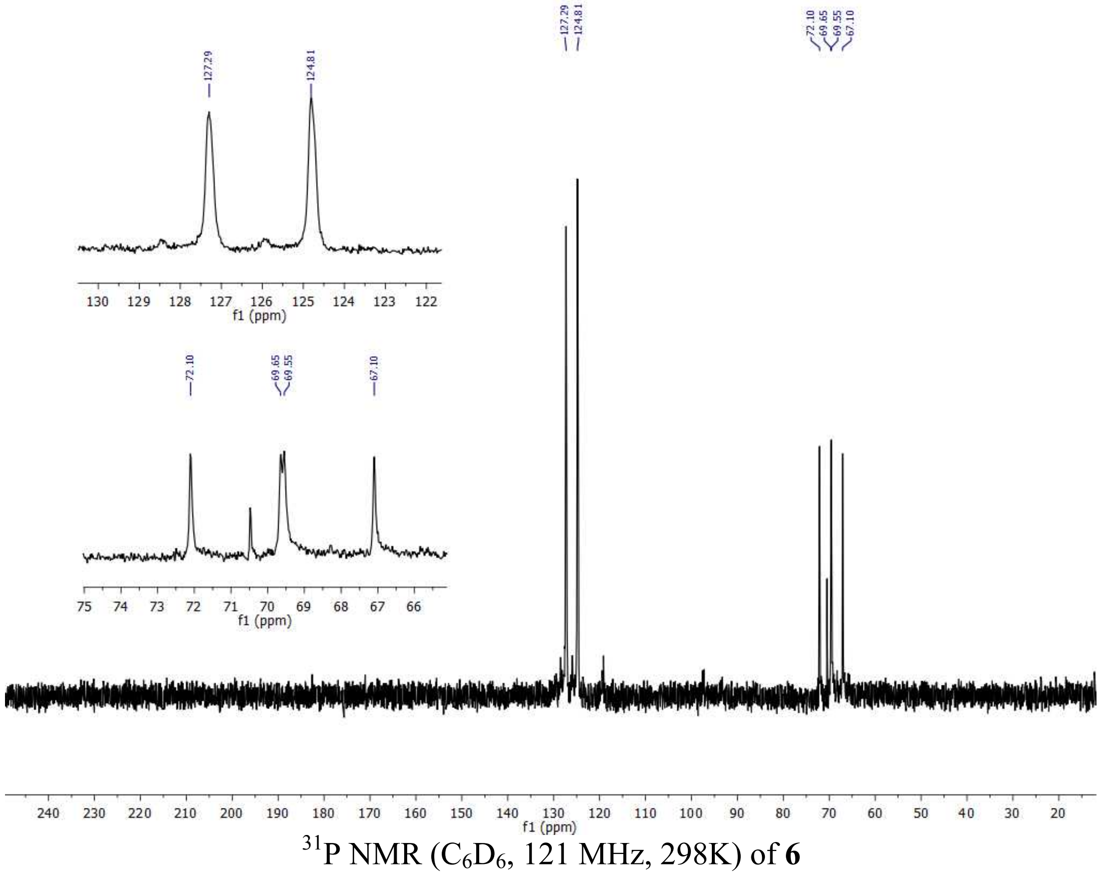

^{31}P NMR (C_6D_6, 121 MHz, 298K) of **6**

Preparation of 7:

To a solution of **6** (60 mg, 0.040 mmol) in benzene (1 mL) was added excess 15-crown-5-ether (0.1 mL). Upon standing single crystals of **7** formed. The solution was decanted off the solid washed with little pentane and the remaining solvent evaporated to give **7** as slightly yellow solid (40 mg, 58%). **$^{31}P\{^1H\}$ NMR** (C_6D_6, 121 MHz, 298K) δ [ppm] = +119.7 (d, J = 310 Hz), + 78.8 (t, J = 310 Hz).

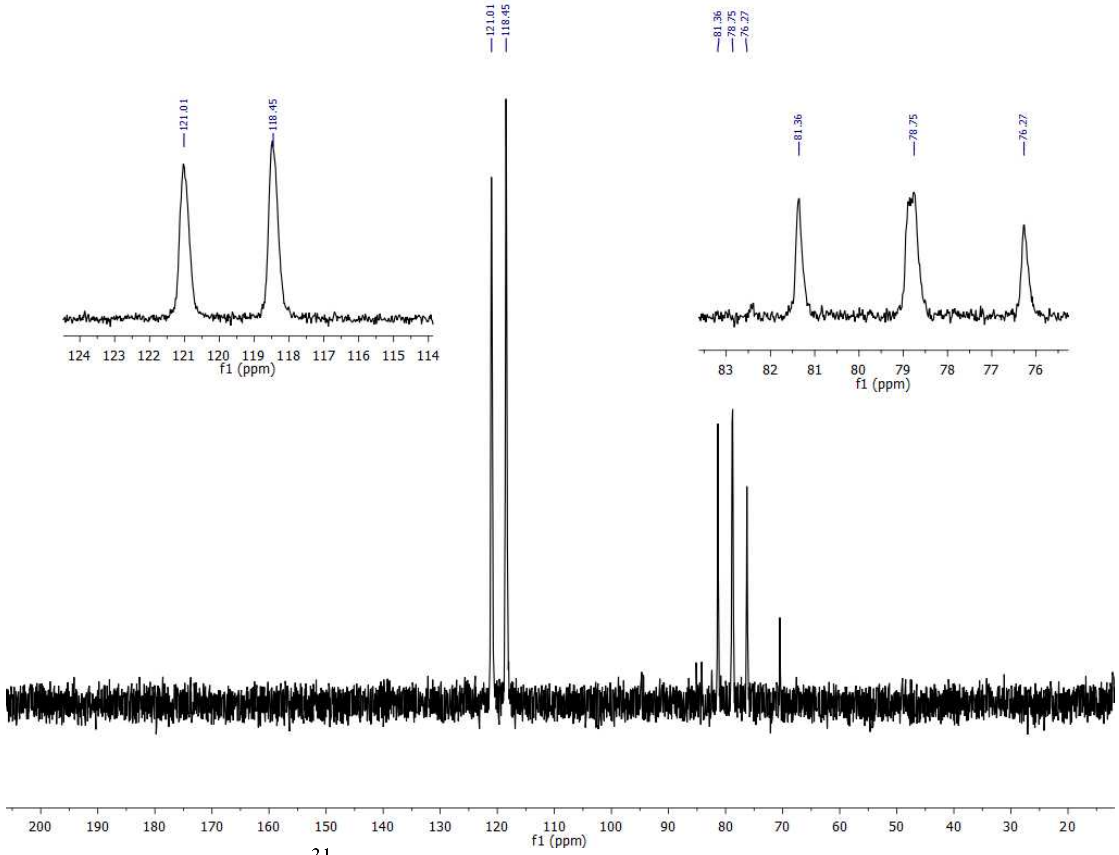

^{31}P NMR (C_6D_6, 121 MHz, 298K) of **7**

Labeling experiment:

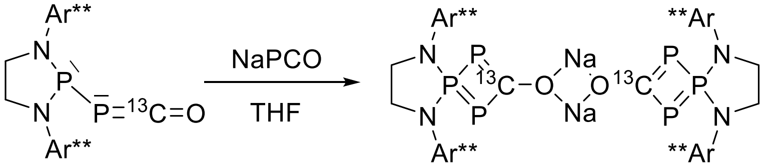

1^{Ar**} **6**

To a solution of ^{13}C labelled **1^{Ar**}**,[1] (50 mg, 0.035 mmol) in a C_6D_6(0.5 mL)/THF(0.2 mL) mixture was added NaPCO(dioxane)$_{3.0}$ (36 mg, 0.10 mmol, 3eq.). The ^{13}C NMR and ^{31}P NMR was directly measured (see below). While the ^{13}C NMR of the non-labelled experiment did not allow the identification of the carbon resonance of the phosphorus cycle, the ^{13}C NMR of the labelled one did (see below).

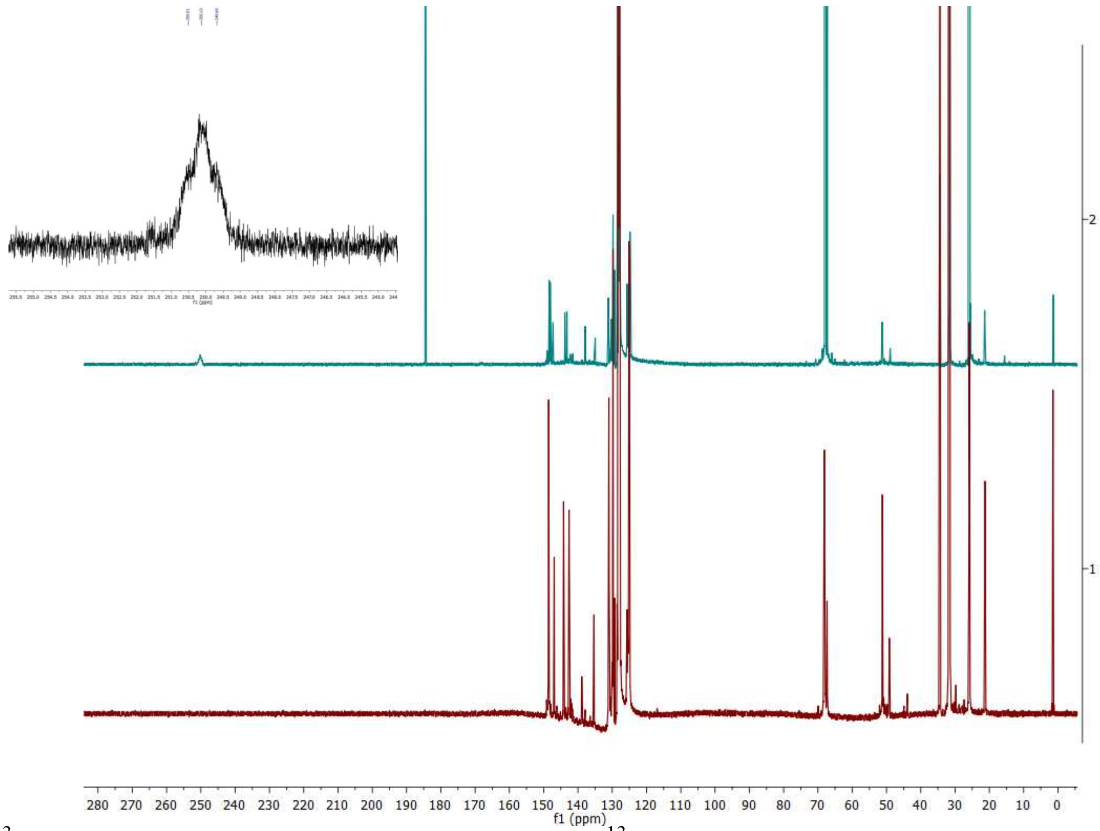

^{13}C NMR (C_6D_6, 125 MHz, 298K) overlay of **6** ^{13}C-labelled (top) and unlabeled (below).

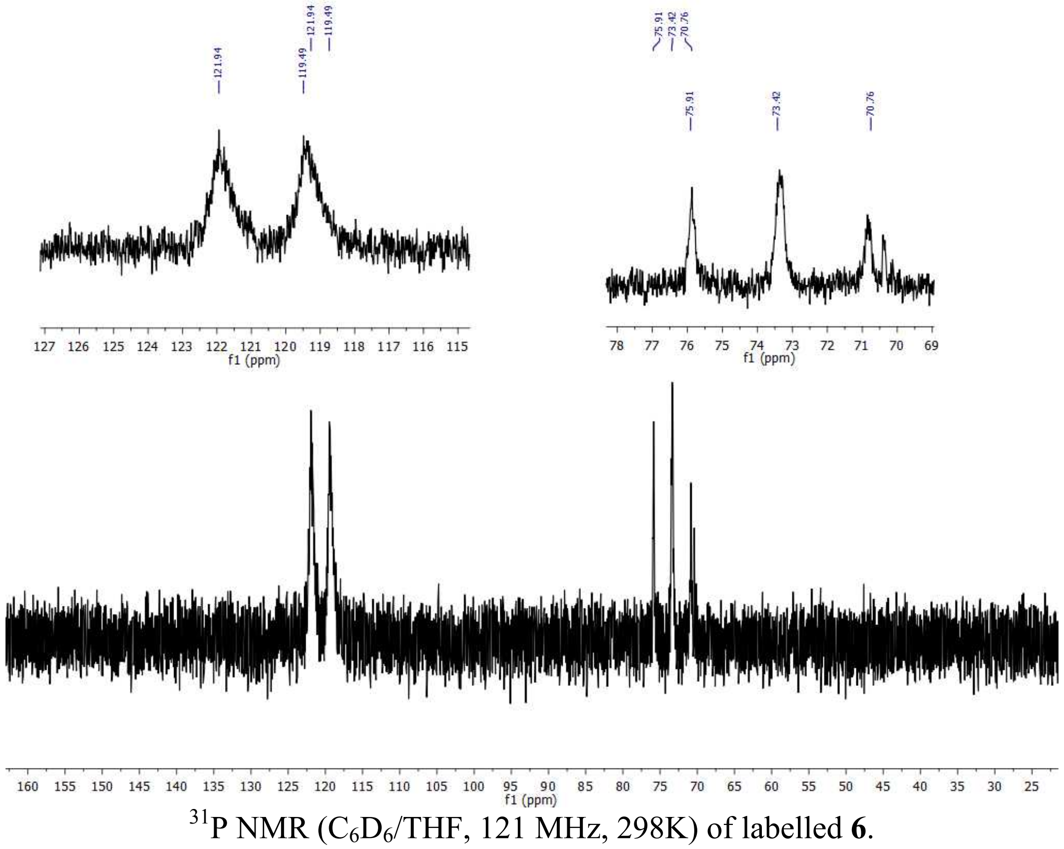

^{31}P NMR (C_6D_6/THF, 121 MHz, 298K) of labelled **6**.

Preparation of 8:

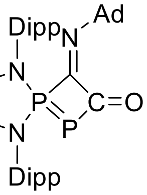

1[Dipp] (10 mg, 0.021 mmol) and 1-adamantyl isocyanide (4 mg, 0.025 mmol) were dissolved in pentane (0.5 mL). The mixture was left overnight, upon which yellow crystals formed. The solution was decanted yielding 11 mg of **8** (85% yield). M.p.: 165 ºC (dec.). **^{1}H NMR** (C_6D_6, 500 MHz, 298K): δ [ppm] = 7.14 (br, 2H), 7.07 (br m, 4H), 3.92 (sept, *J* = 6.8 Hz, 2H), 3.60 (m, 2H), 3.45 (sept, *J* = 6.8 Hz, 2H), 3.25 (m, 2H), 2.34 (br, 6H), 2.02 (br, 3H), 1.75 (d, *J* = 12.0 Hz, 3H), 1.56 (d, *J* = 12.0 Hz, 3H), 1.37 (d, *J* = 6.8 Hz, 6H), 1.35 (d, *J* = 6.8 Hz, 6H), 1.20 (d, *J* = 6.8 Hz, 6H), 1.17 (d, *J* = 6.8 Hz, 6H); **^{13}C NMR** (C_6D_6, 125 MHz, 298K): δ [ppm] = 210.8 (dd, J_{PC} = 90.0 Hz, J_{PC} = 65.9 Hz), 180.8 (dd, J_{PC} = 86.3 Hz, J_{PC} = 2.7 Hz), 150.2, 149.0, 134.4, 134.3, 129.3, 124.9, 61.3, 61.2, 51.9, 51.8, 43.2, 36.6, 30.5, 29.6, 27.9, 26.6, 26.5, 25.2, 24.0, 23.9; **^{31}P NMR** (C_6D_6, 121 MHz, 298K): δ [ppm] = 148.0 (d, J_{PP} = 369.5 Hz), 88.3 (d, J_{PP} = 369.5 Hz).

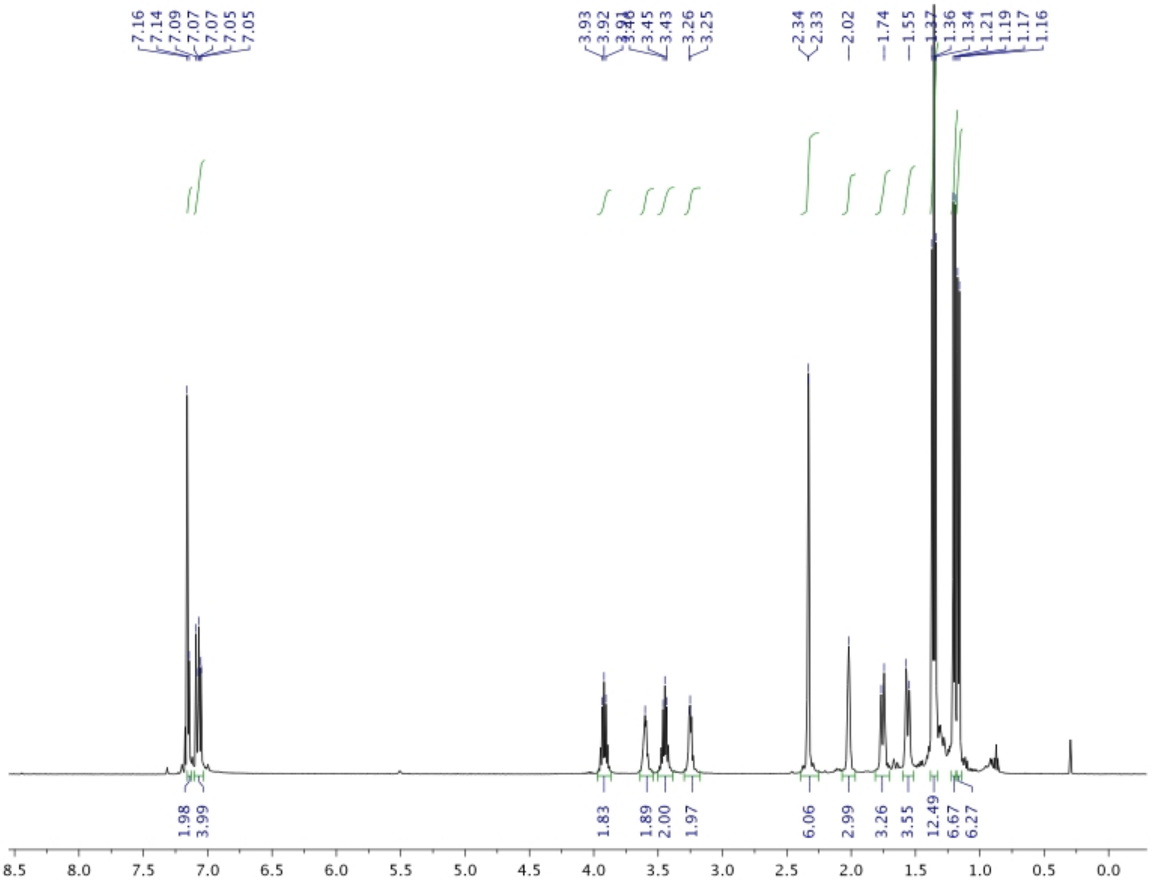

^{1}H NMR (C_6D_6, 500 MHz, 298K) of **8**

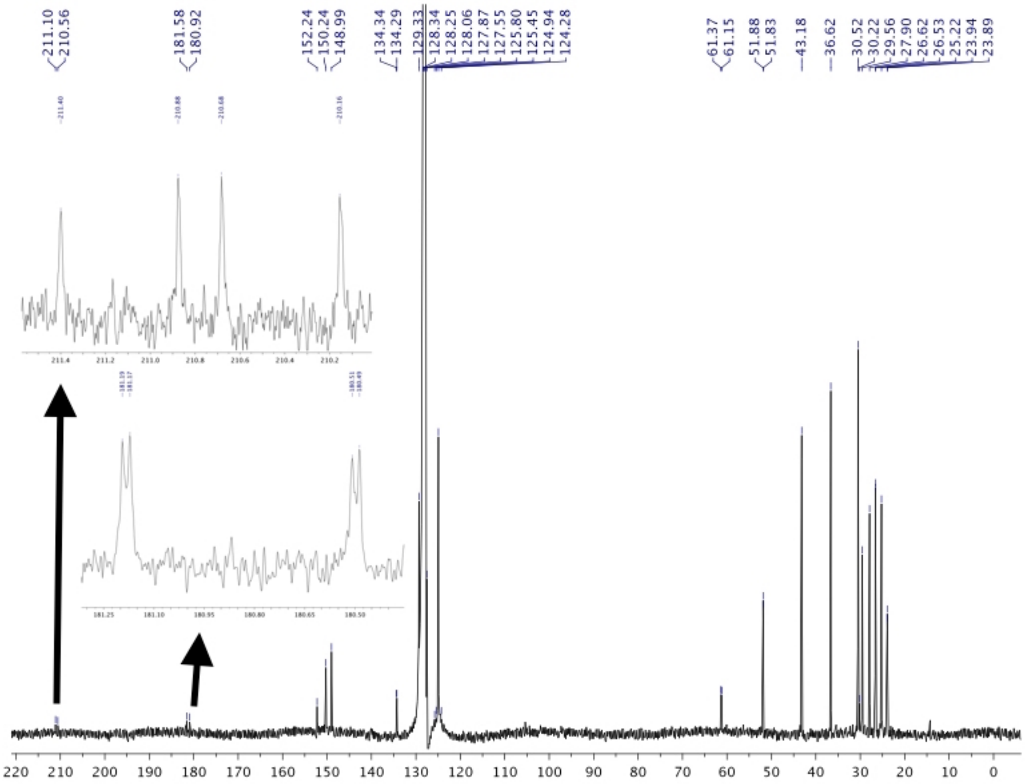

^{13}**C NMR** (C_6D_6, 125 MHz, 298K) of **8**

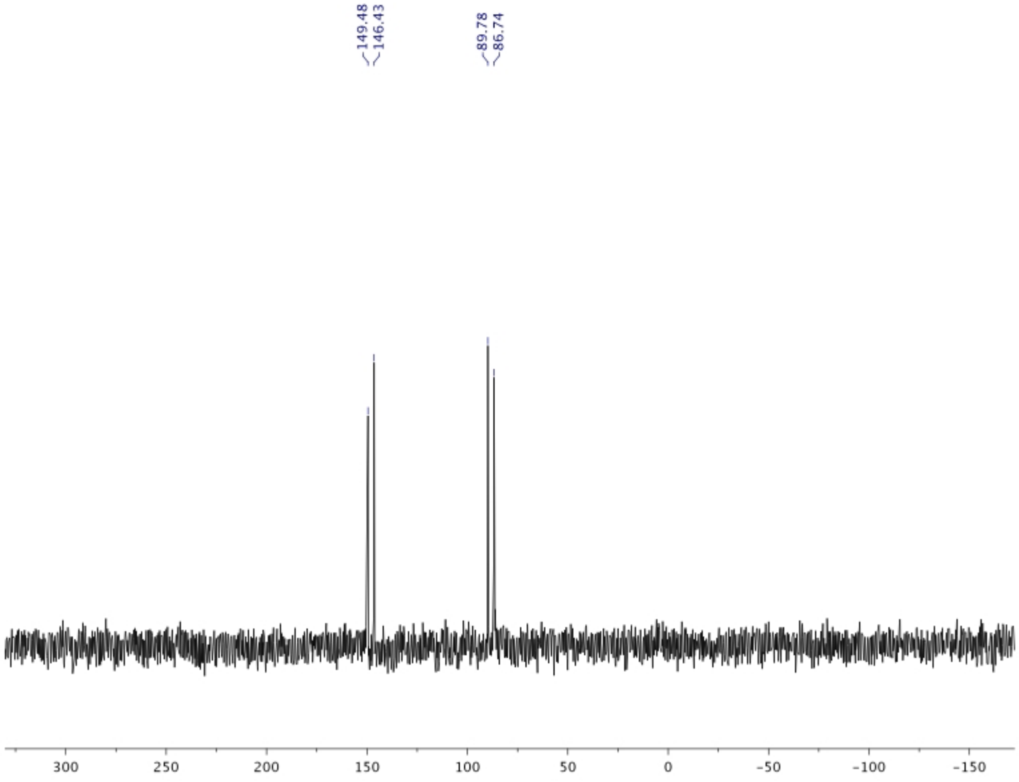

^{31}**P NMR** (C_6D_6, 121 MHz, 298K) of **8**

Preparation of 9:

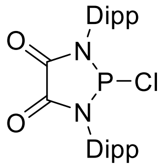

To a solution of N,N-bis(2,6-diisopropylphenyl)oxalamide[2] (3.5 g, 8.57 mmol) in THF (80 mL) was added at -78 °C dry NEt_3 (17.9 mL, 128 mmol, 15 eq.) followed by distilled PCl_3 (1.0 mL, 8.57 mmol, 1.0 eq.). The solution was stirred for 30 min at this temperature and then warmed up over night. The solvent was evaporated under reduced pressure and the compound extracted with benzene (60 mL). Evaporation of the solvent affords **9** as colorless solid (2.4 g, 5.1 mmol, 59%). **^{1}H NMR** (C_6D_6, 300 MHz, 298K): δ [ppm] = 7.24-7.19 (m, 2H), 7.17-7.12 (m, 2H), 7.04-7.01 (m, 2H), 3.37 (sept., *J* = 6.8 Hz, 2H), 3.05-2.97 (m, 2H), 1.25 (d, *J* = 6.8 Hz, 6H), 1.23 (d, *J* = 6.8 Hz, 6H), 1.13 (d, *J* = 6.8 Hz, 6H), 1.02 (d, *J* = 6.8Hz, 6H); **^{13}C NMR** (C_6D_6, 75 MHz, 298K): δ [ppm] = 156.8 (d, *J* = 3.6 Hz), 149.2, 146.0, 130.8, 128.6, 125.7, 124.7, 30.6, 30.5, 29.9, 25.0, 24.9, 24.0, 23.5, 23.5; **^{31}P NMR** (C_6D_6, 121 MHz, 298K): δ [ppm] = 118.7 (s); **HR-MS**: m/z calculated for $[M+H]^+$ $[C_{26}H_{35}ClN_2O_2P]^+$ 473.2119; found 473.2123.

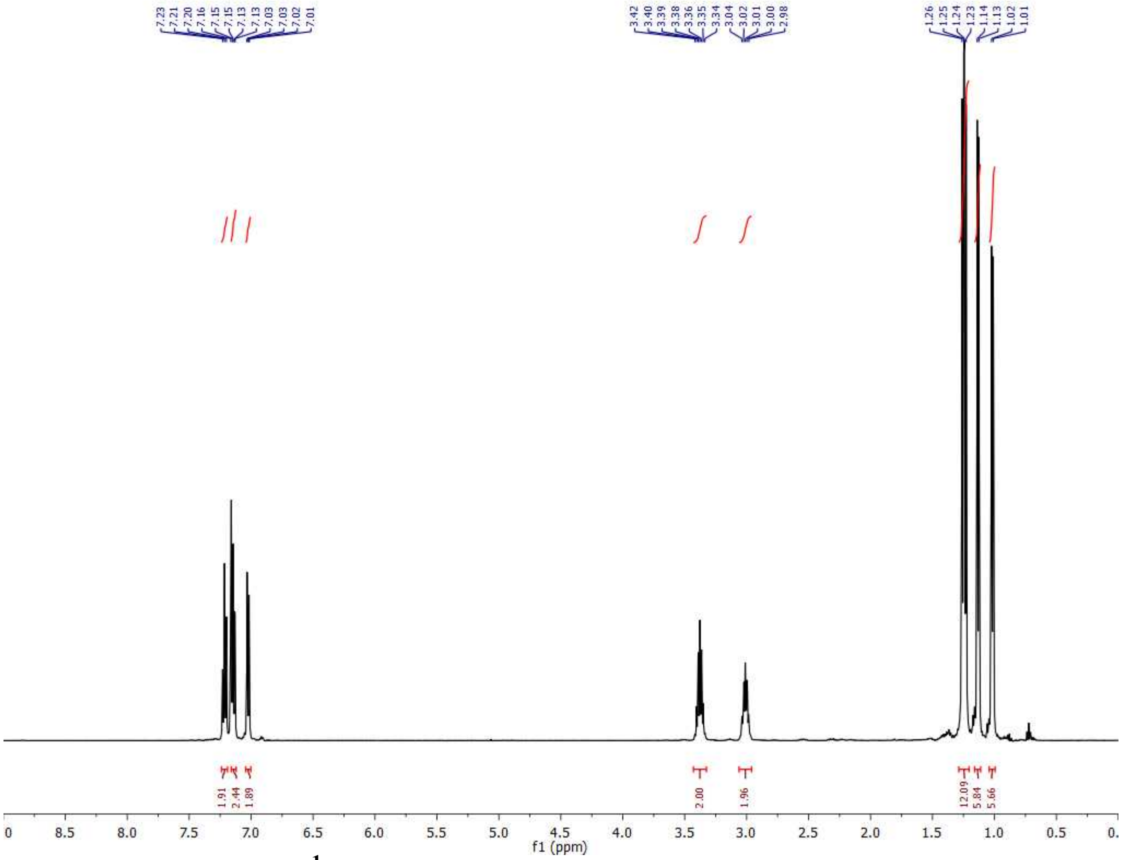

^{1}H NMR (C_6D_6, 300 MHz, 298K) of **9**

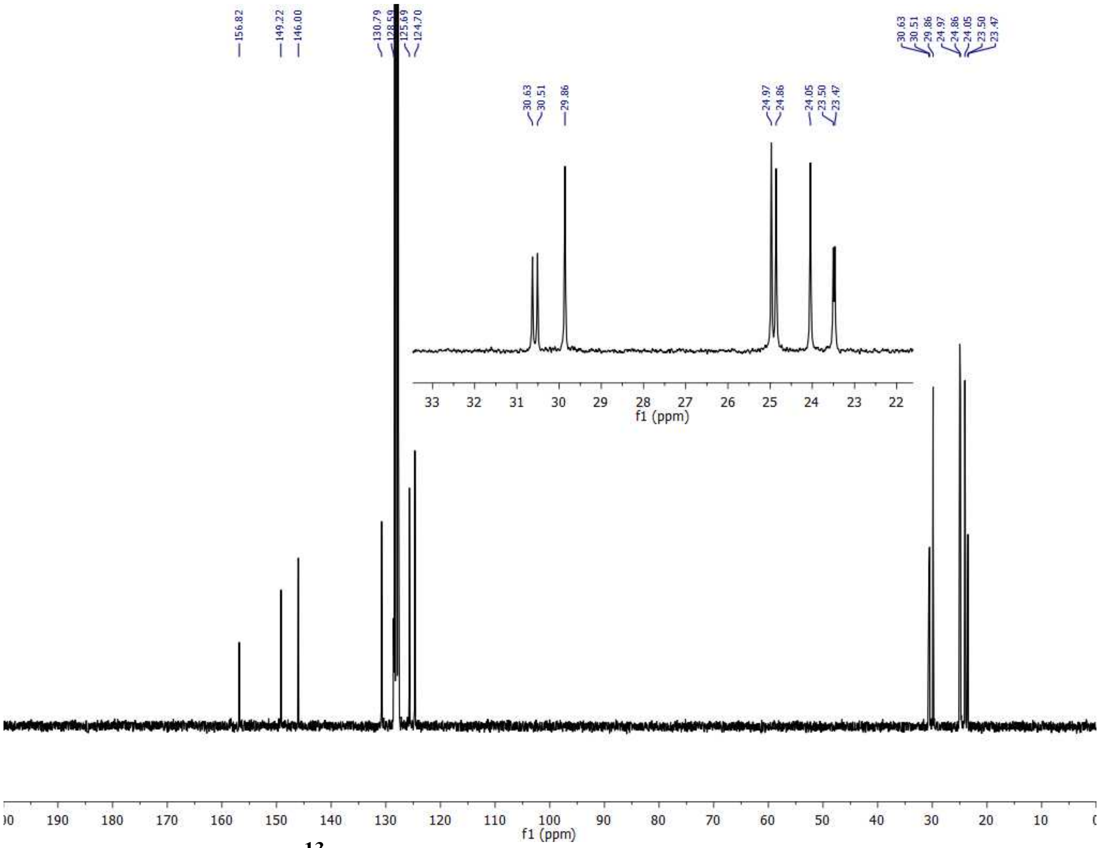

^{13}C NMR (C_6D_6, 75 MHz, 298K) of **9**

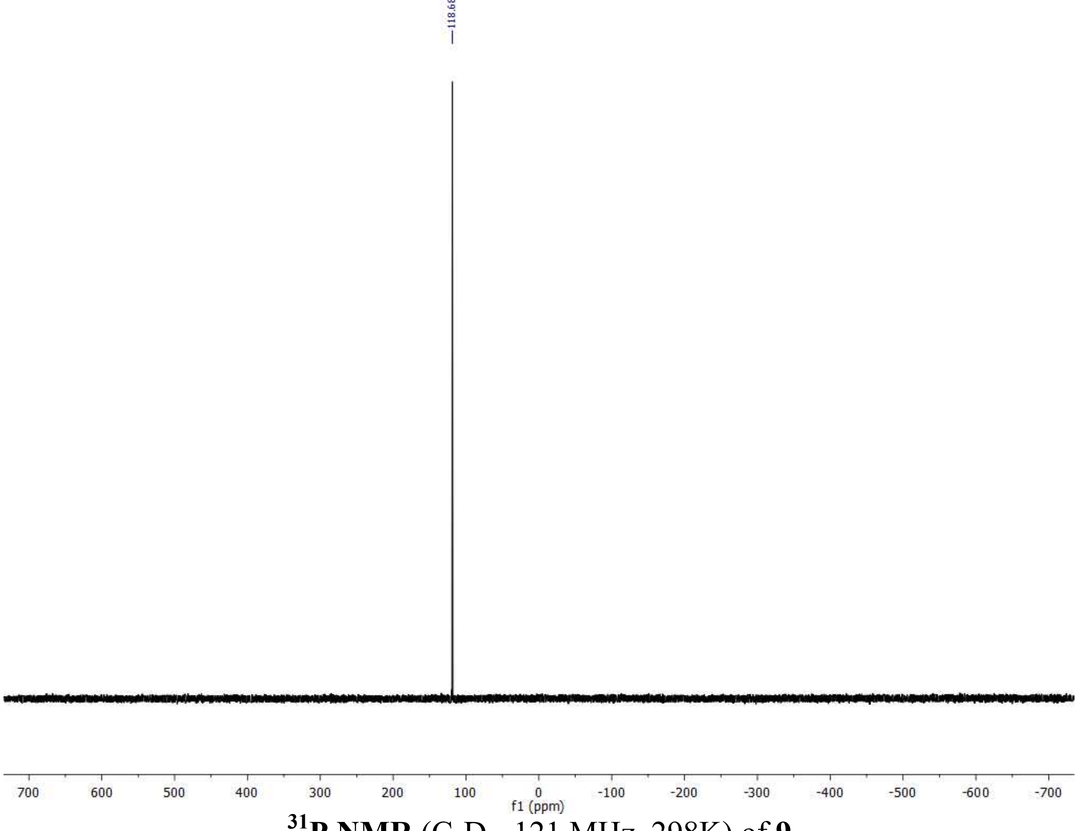

^{31}P NMR (C_6D_6, 121 MHz, 298K) of **9**

Preparation of 10:

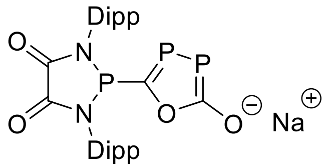

To a solution of **9** (100 mg, 0.21 mmol) in THF (3 mL) was added NaPCO(dioxane)$_{3.0}$ (150 mg, 0.433 mmol, 2.1 eq) at room temperature and stirred for 20 min. The clear solution turns into a dark orange suspension. The suspension is filtered and the remaining solid dissolved in CH_3CN (4 mL). Layering the CH_3CN solution with Et_2O (10 mL) gives single crystals for x-ray diffraction. Removal of the solvent of the THF solution also affords the desired compound **10** as yellow solid (combined masses m = 87 mg, 72%). **^{1}H NMR** (CD_3CN, 300 MHz, 298K): δ [ppm] = 7.44-7.36 (m, 2H), 7.34-7.30 (m, 2H), 7.28-7.24 (m, 2H), 3.69-3.60 (m, 8H, coord. THF), 3.26-3.13 (m, 4H), 1.84-1.78 (m, 8H, coord. THF), 1.36 (d, J = 6.8 Hz, 6H), 1.18 (d, J = 6.8 Hz, 6H), 1.13 (d, J = 6.8 Hz, 6H), 0.83 (d, J = 6.8 Hz, 6H); **^{13}C NMR** (CD_3CN, 75 MHz, 298K): δ [ppm] = 203.8 (d, J_{CP} = 105 Hz), 160.7, 156.1 (m), 149.9, 147.1, 130.9, 130.9, 130.4, 126.6, 125.1, 67.6 (coordinated THF), 30.6, 30.5, 29.1, 26.2 (coordinated THF), 25.0, 24.7, 24.4, 23.8, 23.8; **^{31}P NMR** (CD_3CN,121 MHz, 298K): δ [ppm] = +322.7 (dd, J = 466 Hz, 282 Hz); 47.9 (d, J = 282 Hz); 45.3 (d, J = 466 Hz).

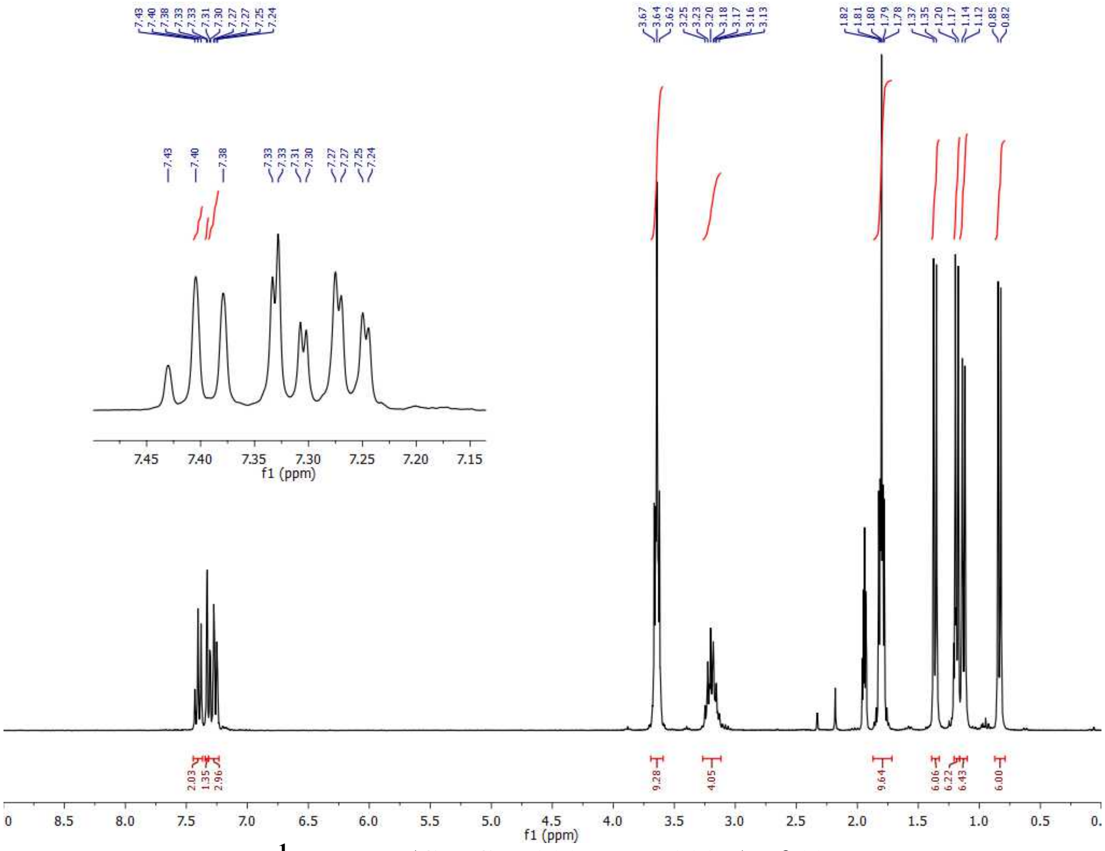

^{1}H NMR (CD_3CN, 300 MHz, 298K) of **10**

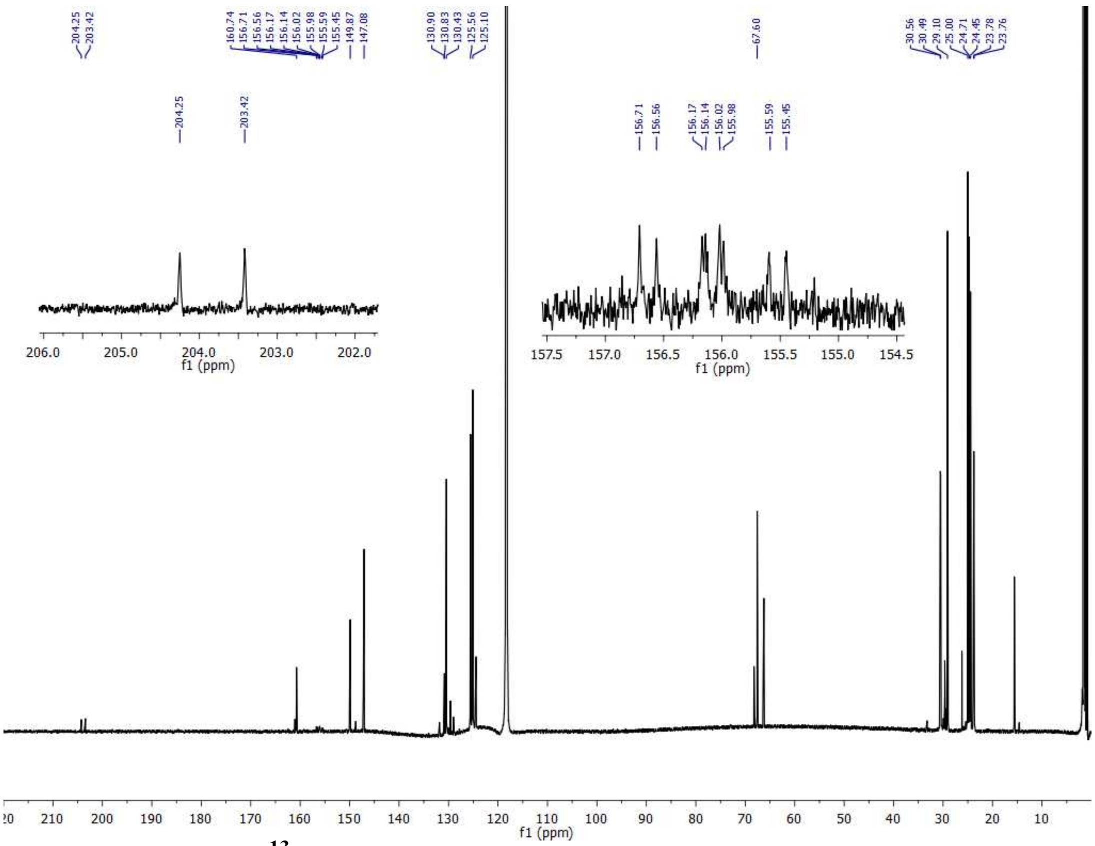

^{13}C NMR (CD$_3$CN, 125 MHz, 298K) of **10**

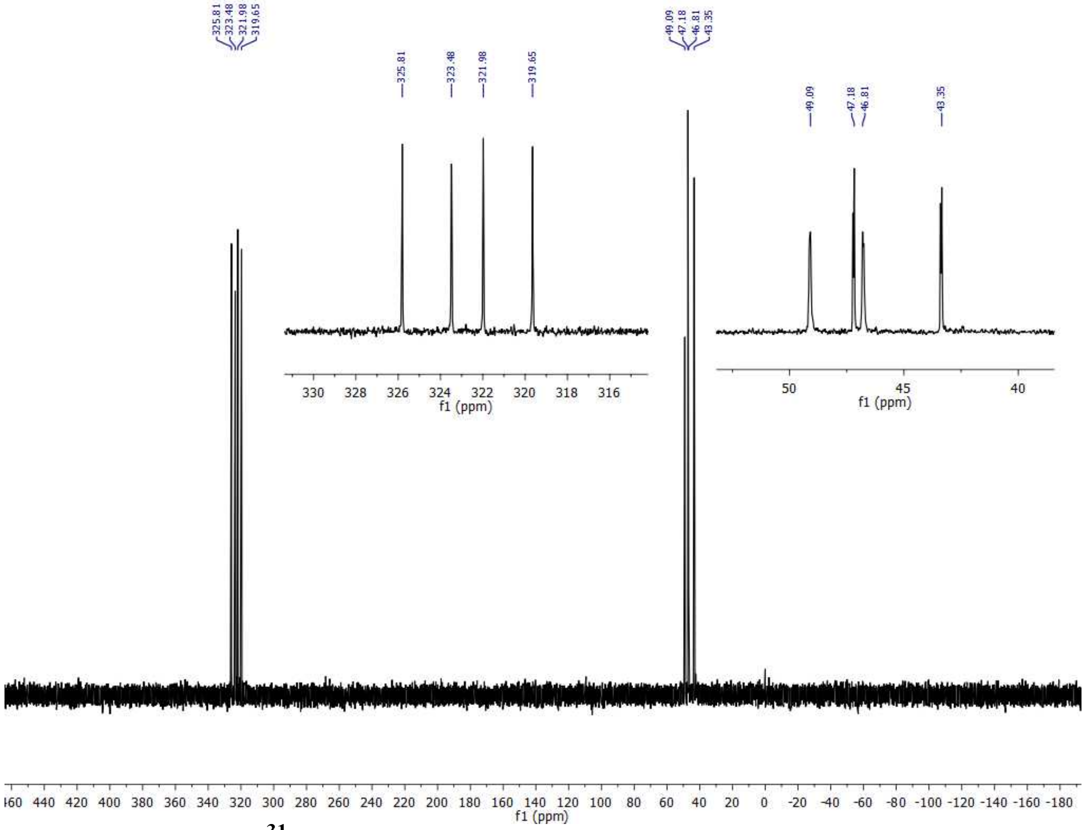

^{31}P NMR (CD$_3$CN, 121 MHz, 298K) of **10**

3. X-ray data

Xray Table 1. Crystal data and structure refinement for 4.

Identification code	BH3
Empirical formula	$C_{27}H_{41}BN_2OP_2$
Formula weight	482.37
Temperature/K	100.0
Crystal system	orthorhombic
Space group	Pnma
a/Å	12.9335(12)
b/Å	20.8660(18)
c/Å	10.2861(9)
α/°	90
β/°	90
γ/°	90
Volume/Å^3	2775.9(4)
Z	4
ρ_{calc}g/cm^3	1.154
μ/mm^{-1}	1.571
F(000)	1040.0
Crystal size/mm^3	0.3 × 0.24 × 0.2
Radiation	CuKα (λ = 1.54178)
2Θ range for data collection/°	8.474 to 136.656
Index ranges	$-15 \le h \le 11, -25 \le k \le 24, -12 \le l \le 12$
Reflections collected	24990
Independent reflections	2624 [R_{int} = 0.0474, R_{sigma} = 0.0236]
Data/restraints/parameters	2624/24/182
Goodness-of-fit on F^2	1.082
Final R indexes [I>=2σ (I)]	R_1 = 0.0597, wR_2 = 0.1716
Final R indexes [all data]	R_1 = 0.0641, wR_2 = 0.1764
Largest diff. peak/hole / e Å^{-3}	0.86/-0.94

Summary of Data CCDC 1432815

Compound Name: 4

Formula: C27 H41 B1 N2 O1 P2,x(C1 O1),0.16(B1)

Unit Cell Parameters: a 12.9335(12) b 20.8660(18) c 10.2861(9) Pnma

---

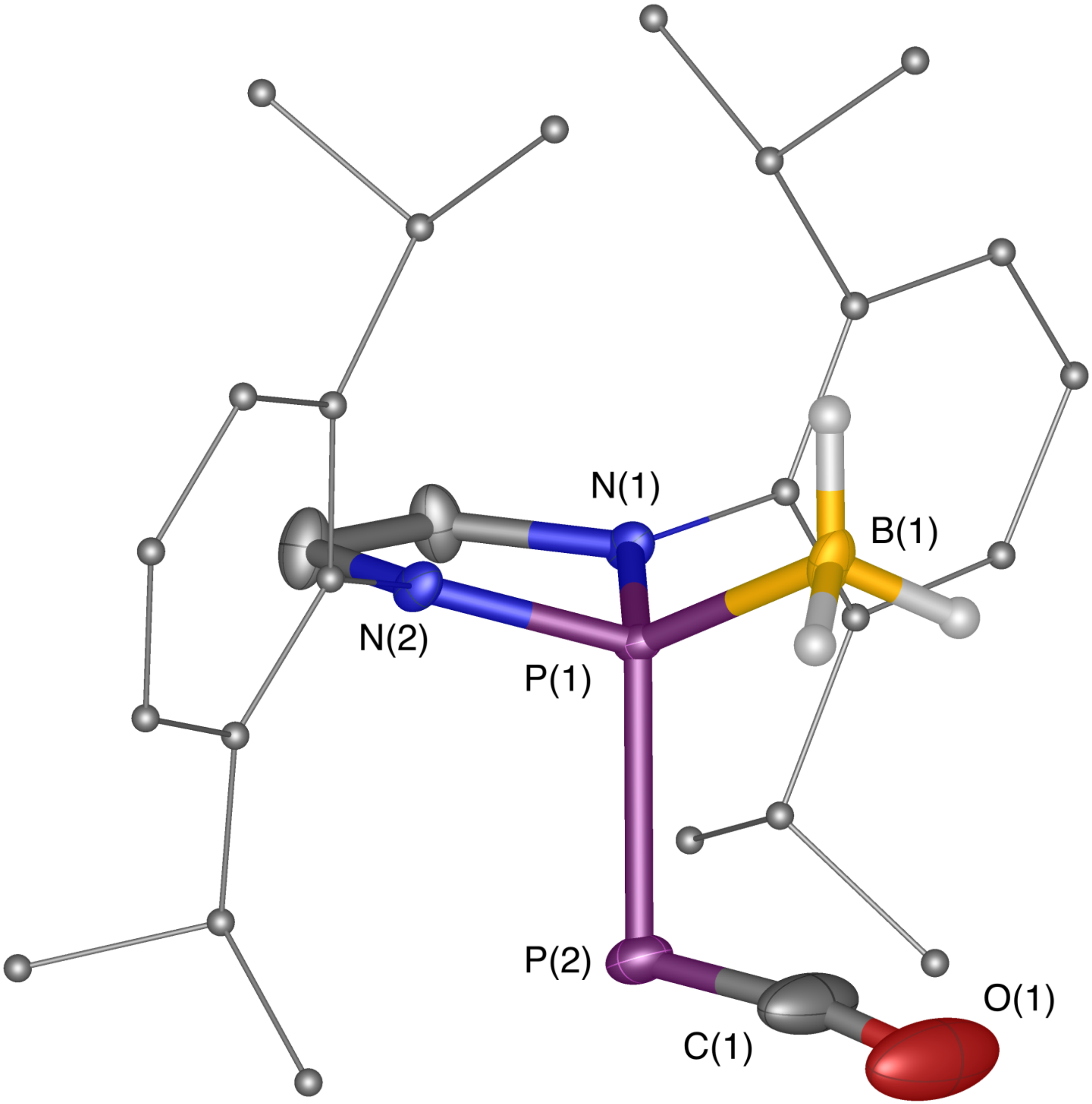

Structure of 4 in the solid state. All hydrogen atoms, except for BH_3, omitted for clarity

Xray Table 2. Crystal data and structure refinement for 5.

Identification code	BCF
Empirical formula	$C_{45}H_{38}BF_{15}N_2OP_2$
Formula weight	980.52
Temperature/K	100
Crystal system	monoclinic
Space group	$P2_1/n$
a/Å	14.0829(9)
b/Å	20.6872(19)
c/Å	15.9795(14)
α/°	90
β/°	99.559(5)
γ/°	90
Volume/$Å^3$	4590.8(7)
Z	4
$\rho_{calc}g/cm^3$	1.419
μ/mm^{-1}	0.193
F(000)	2000.0
Crystal size/mm^3	0.32 × 0.25 × 0.25
Radiation	MoKα (λ = 0.71073)
2Θ range for data collection/°	3.532 to 50.062
Index ranges	$-16 \leq h \leq 14, -22 \leq k \leq 24, -13 \leq l \leq 19$
Reflections collected	24382
Independent reflections	8073 [R_{int} = 0.1023, R_{sigma} = 0.1109]
Data/restraints/parameters	8073/0/603
Goodness-of-fit on F^2	0.983
Final R indexes [I>=2σ (I)]	R_1 = 0.0572, wR_2 = 0.1234
Final R indexes [all data]	R_1 = 0.1148, wR_2 = 0.1501
Largest diff. peak/hole / e $Å^{-3}$	0.35/-0.31

--

Summary of Data CCDC 1432814

--

Compound Name: 5
Formula: C45 H38 B1 F15 N2 O1 P2
Unit Cell Parameters: a 14.0829(9) b 20.6872(19) c 15.9795(14) P21/n

--

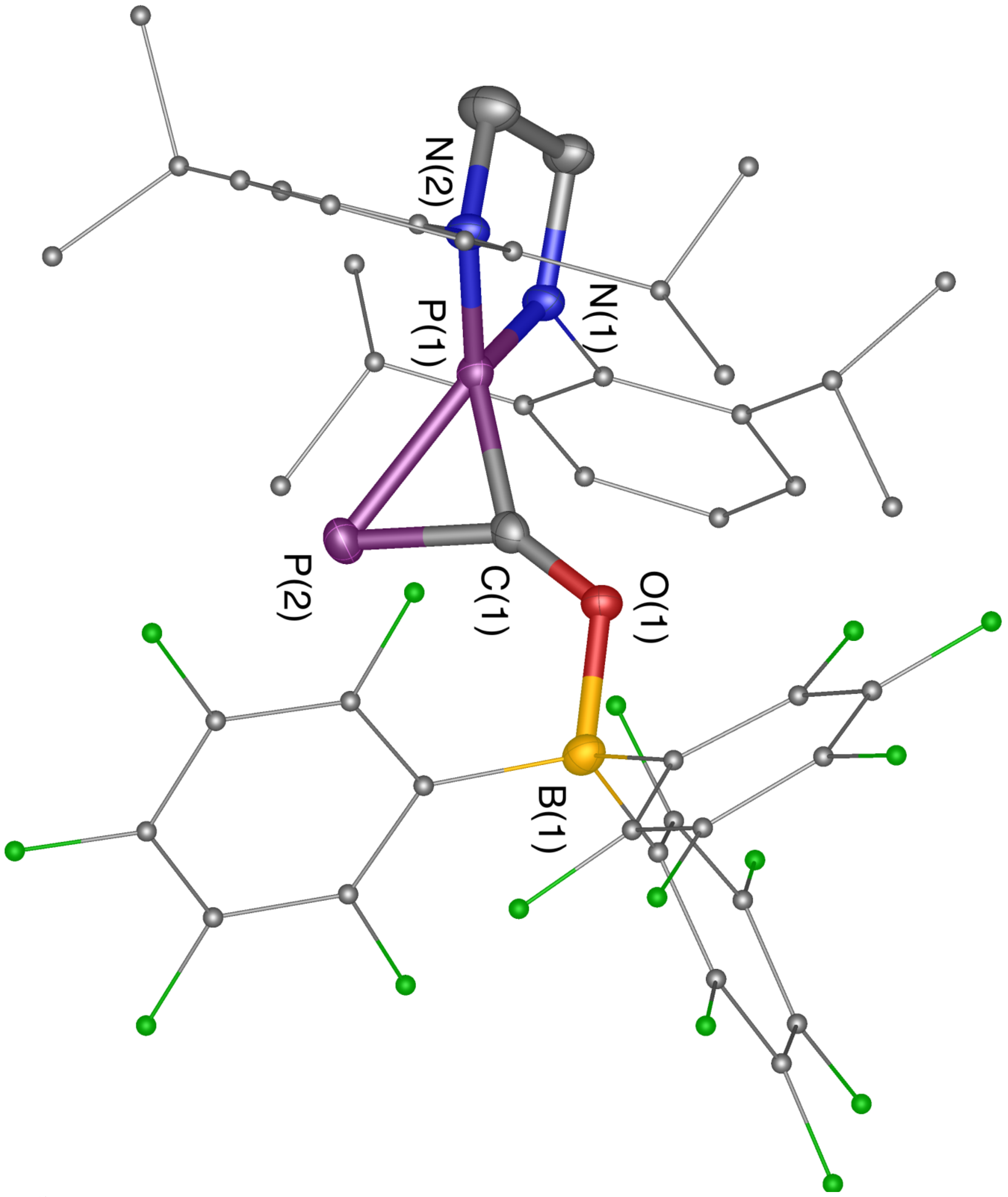

Structure of 5 in the solid state. All hydrogen atoms omitted for clarity

Xray Table 3 Crystal data and structure refinement for 6.

Identification code	**6**
Empirical formula	$C_{218}H_{276}N_4Na_2O_6P_6$
Formula weight	3280.21
Temperature/K	100.0
Crystal system	triclinic
Space group	P-1
a/Å	14.6157(11)
b/Å	18.3339(15)
c/Å	23.554(2)
α/°	85.401(7)
β/°	81.374(6)
γ/°	78.671(5)
Volume/Å^3	6110.2(9)
Z	1
ρ_{calc}g/cm^3	0.891
μ/mm^{-1}	0.783
F(000)	1772.0
Crystal size/mm^3	0.3 × 0.1 × 0.1
Radiation	CuKα (λ = 1.54178)
2Θ range for data collection/°	3.8 to 137.65
Index ranges	$-17 \le h \le 17$, $-19 \le k \le 21$, $-28 \le l \le 27$
Reflections collected	102657
Independent reflections	21898 [R_{int} = 0.0672, R_{sigma} = 0.0576]
Data/restraints/parameters	21898/30/1157
Goodness-of-fit on F^2	1.020
Final R indexes [I>=2σ (I)]	R_1 = 0.0850, wR_2 = 0.2248
Final R indexes [all data]	R_1 = 0.1025, wR_2 = 0.2404
Largest diff. peak/hole / e Å^{-3}	1.34/-0.65

--

Summary of Data CCDC 1528506

--

Compound Name: 6

Formula: C218 H276 N4 Na2 O6 P6

Unit Cell Parameters: a 14.6157(11) b 18.3339(15) c 23.554(2) P-1

--

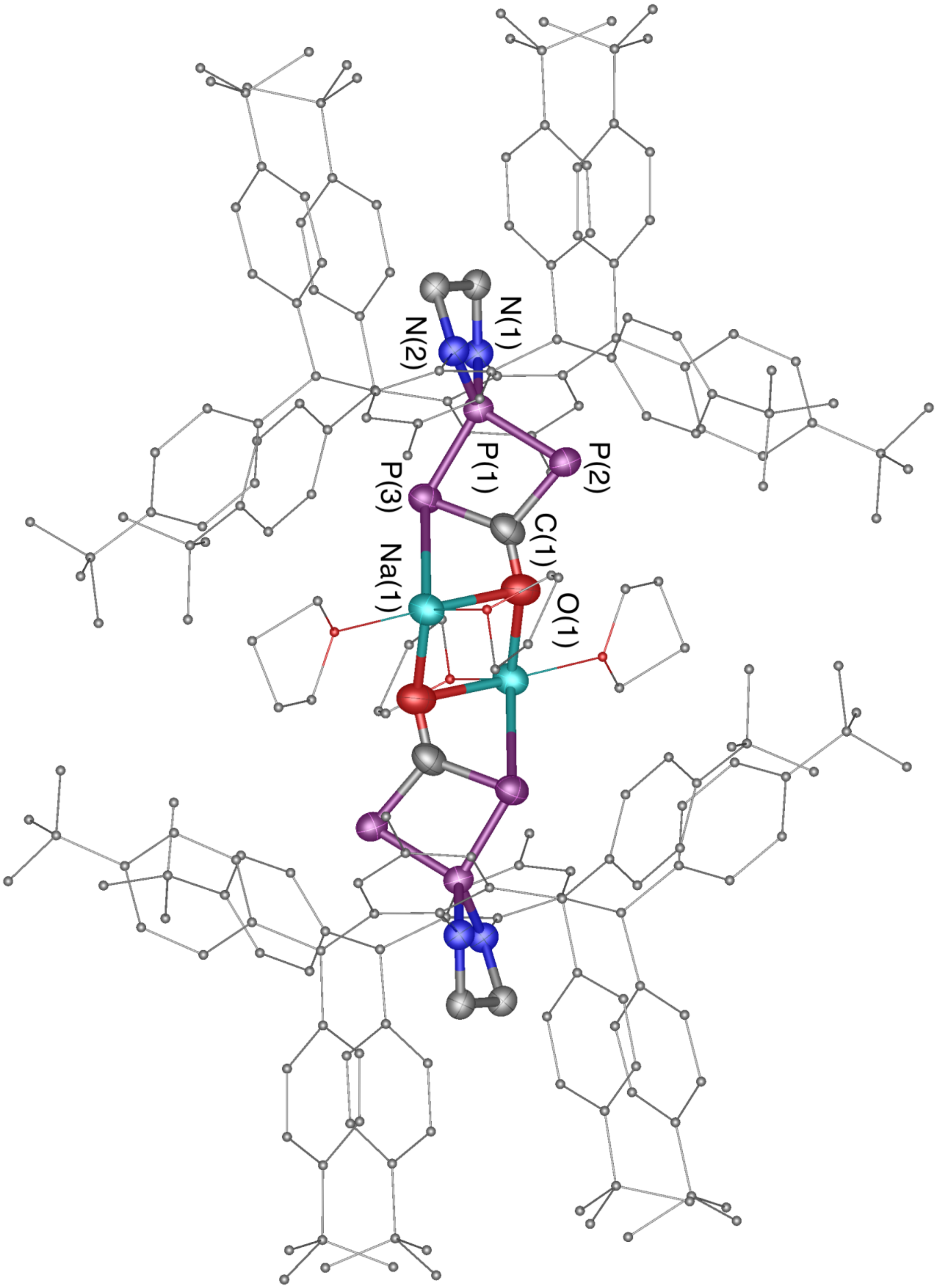

Structure of **6** in the solid state. All hydrogen atoms omitted for clarity.

Xray Table 4 Crystal data and structure refinement for 7.

Identification code	Crown
Crystal system	trigonal
Space group	P-3
a/Å	26.1613(10)
b/Å	26.1613(10)
c/Å	38.921(2)
α/°	90
β/°	90
γ/°	120
Volume/Å^3	23069(2)
Z	8
ρ_{calc}g/cm^3	1.313
μ/mm^{-1}	1.071
F(000)	9688.0
Crystal size/mm^3	0.1 × 0.2 × 0.15
Radiation	CuKα (λ = 1.54178)
2Θ range for data collection/°	3.9 to 101.108
Index ranges	-26 ≤ h ≤ 26, -25 ≤ k ≤ 26, -38 ≤ l ≤ 38
Reflections collected	136290
Independent reflections	16142 [R_{int} = 0.0937, R_{sigma} = 0.0663]
Data/restraints/parameters	16142/1234/1329
Goodness-of-fit on F^2	2.429

Structure could not be refined below 16%. Hence the corresponding data which only allowed us to confirm the formation of this species has not been submitted to the CCDC.

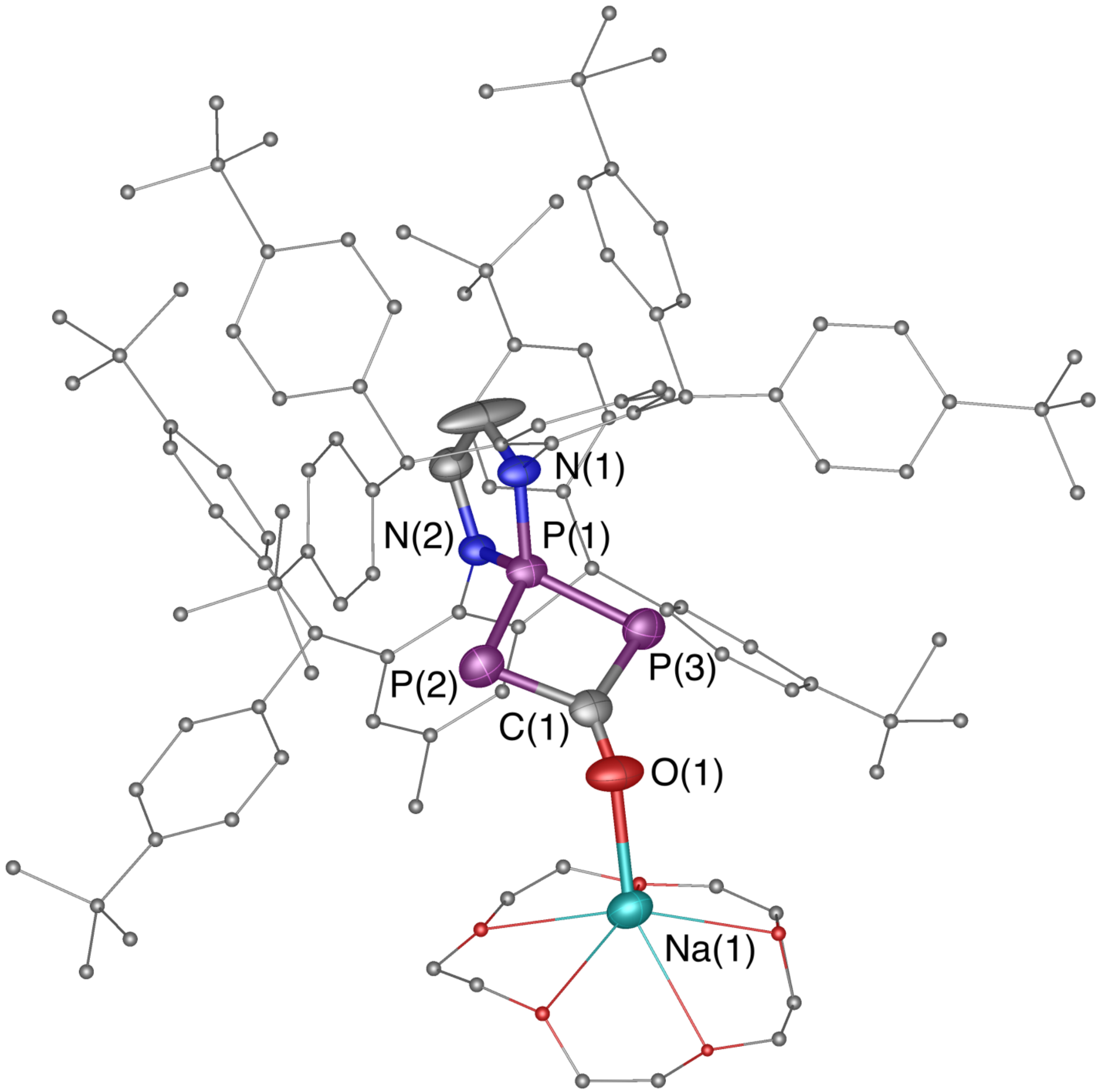

Structure of **7** in the solid state. All hydrogen atoms omitted for clarity

Xray Table 5 Crystal data and structure refinement for 8.

Identification code	Adamant
Empirical formula	$C_{38}H_{53}N_3OP_2$
Formula weight	629.77
Temperature/K	100.15
Crystal system	orthorhombic
Space group	Pnma
a/Å	21.5089(11)
b/Å	17.8123(9)
c/Å	9.2449(5)
α/°	90
β/°	90
γ/°	90
Volume/Å^3	3541.9(3)
Z	4
ρ_{calc}g/cm^3	1.181
μ/mm^{-1}	0.156
F(000)	1360.0
Crystal size/mm^3	0.28 × 0.25 × 0.2
Radiation	MoKα (λ = 0.71073)
2Θ range for data collection/°	4.574 to 50.99
Index ranges	$-26 \leq h \leq 22$, $-17 \leq k \leq 21$, $-11 \leq l \leq 11$
Reflections collected	24783
Independent reflections	3402 [R_{int} = 0.0784, R_{sigma} = 0.0495]
Data/restraints/parameters	3402/0/218
Goodness-of-fit on F^2	1.059
Final R indexes [I>=2σ (I)]	R_1 = 0.0483, wR_2 = 0.1031
Final R indexes [all data]	R_1 = 0.0724, wR_2 = 0.1140
Largest diff. peak/hole / e Å^{-3}	0.49/-0.42

--

Summary of Data CCDC 1432817

--

Compound Name: 8

Formula: C38 H53 N3 O1 P2

Unit Cell Parameters: a 21.5089(11) b 17.8123(9) c 9.2449(5) Pnma

--

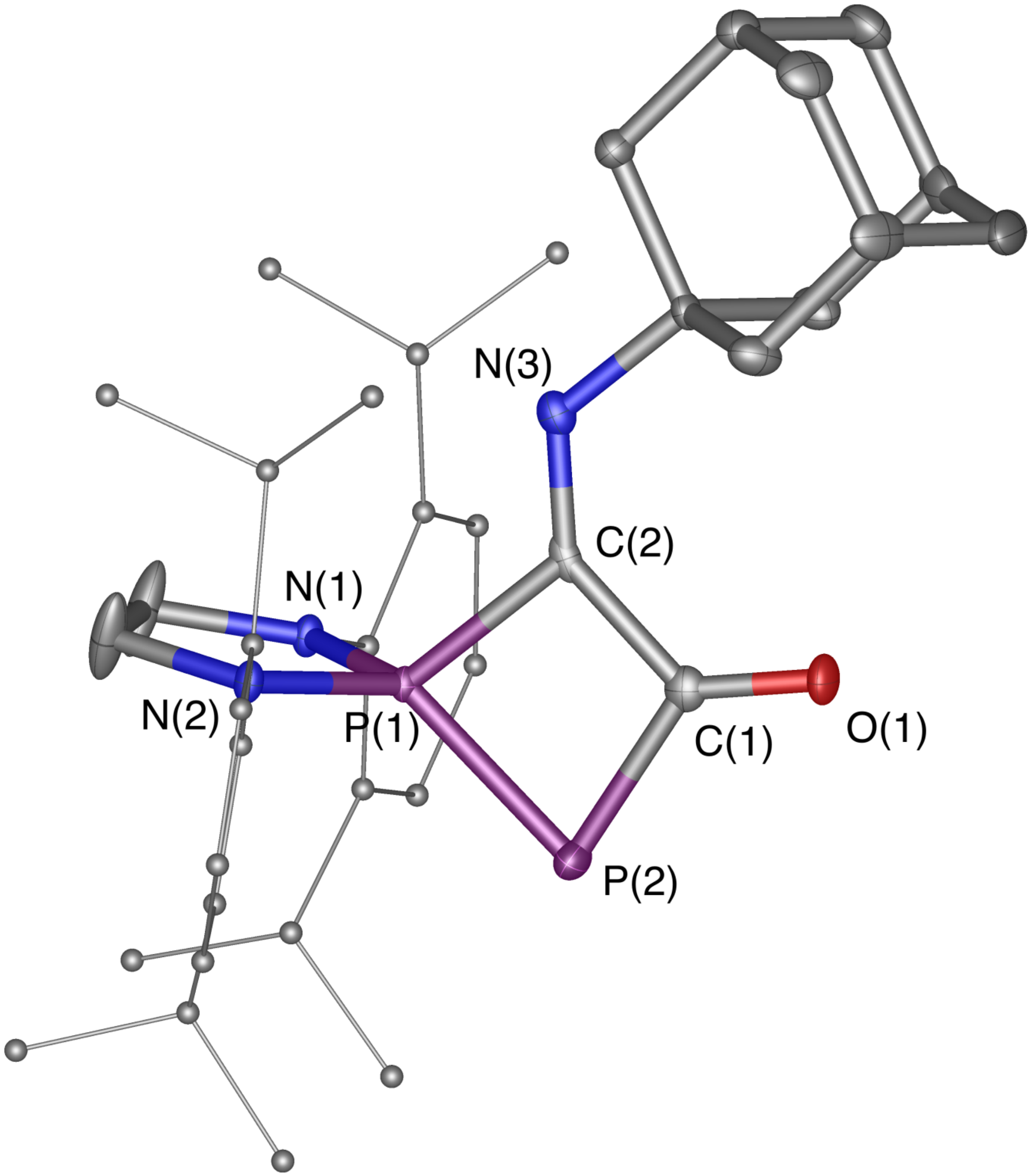

Structure of **8** in the solid state. All hydrogen atoms omitted for clarity.

Xray Table 6 Crystal data and structure refinement for 10.

Identification code	gb_liuamidep4_0m
Empirical formula	C40H58N2NaO7P3
Formula weight	794.78
Temperature/K	100.0
Crystal system	orthorhombic
Space group	P212121
a/Å	13.7779(4)
b/Å	14.7189(4)
c/Å	21.2291(6)
α/°	90
β/°	90
γ/°	90
Volume/Å3	4305.2(2)
Z	4
ρcalcg/cm3	1.226
μ/mm-1	1.755
F(000)	1696.0
Crystal size/mm3	0.1 × 0.1 × 0.1
Radiation	CuKα (λ = 1.54178)
2Θ range for data collection/°	7.308 to 140.016
Index ranges	-16 ≤ h ≤ 16, -17 ≤ k ≤ 16, -25 ≤ l ≤ 25
Reflections collected	77347
Independent reflections	8126 [Rint = 0.0657, Rsigma = 0.0403]
Data/restraints/parameters	8126/30/509
Goodness-of-fit on F2	1.055
Final R indexes [I>=2σ (I)]	R1 = 0.0622, wR2 = 0.1736
Final R indexes [all data]	R1 = 0.0649, wR2 = 0.1762
Largest diff. peak/hole / e Å-3	0.57/-0.40
Flack parameter	0.024(6)

--
Summary of Data CCDC 1528508
--
Compound Name: 10
Formula: (C40 H58 N2 Na1 O7 P3)n
Unit Cell Parameters: a 13.7779(4) b 14.7189(4) c 21.2291(6) P212121
--

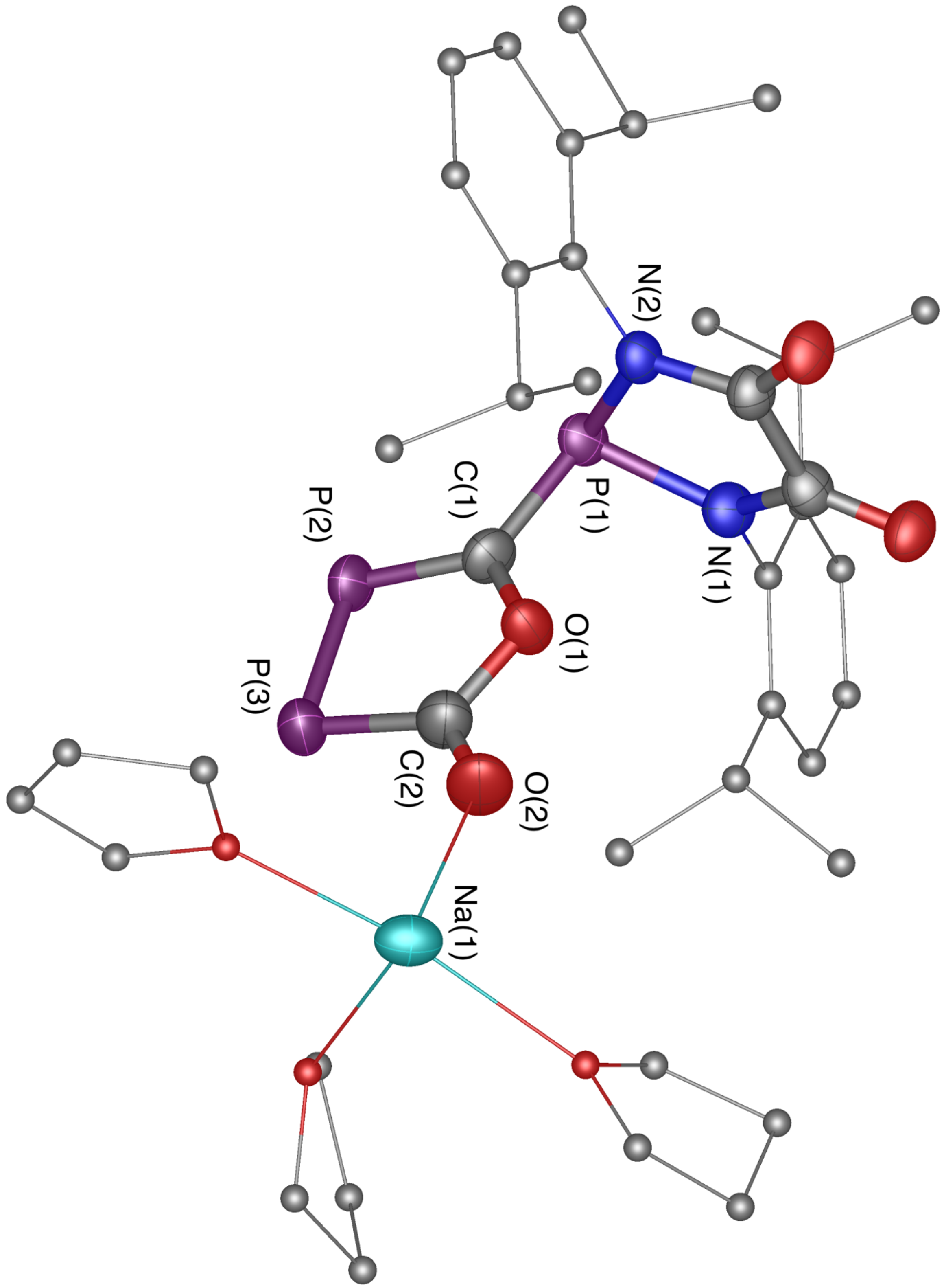

Structure of **10** in the solid state. All hydrogen atoms omitted for clarity.

Xray Table 7 Crystal data and structure refinement for 10'.

Identification code	GB_RJ_MH5_48
Empirical formula	$C_{70}H_{99}N_5Na_2O_{11}P_6$
Formula weight	1418.34
Temperature/K	100.0
Crystal system	monoclinic
Space group	$P2_1/c$
a/Å	27.6426(13)
b/Å	11.7291(6)
c/Å	24.5253(12)
α/°	90
β/°	103.246(2)
γ/°	90
Volume/Å^3	7740.1(7)
Z	4
ρ_{calc}g/cm^3	1.217
μ/mm^{-1}	1.867
F(000)	3016.0
Crystal size/mm^3	0.18 × 0.15 × 0.15
Radiation	CuKα (λ = 1.54178)
2Θ range for data collection/°	6.57 to 140.512
Index ranges	$-33 \leq h \leq 33$, $-14 \leq k \leq 14$, $-29 \leq l \leq 29$
Reflections collected	127799
Independent reflections	14712 [R_{int} = 0.0510, R_{sigma} = 0.0230]
Data/restraints/parameters	14712/0/872
Goodness-of-fit on F^2	1.029
Final R indexes [I>=2σ (I)]	R_1 = 0.0355, wR_2 = 0.0924
Final R indexes [all data]	R_1 = 0.0409, wR_2 = 0.0961
Largest diff. peak/hole / e Å^{-3}	0.76/-0.58

Summary of Data CCDC 1528507

Compound Name: 10b

Formula: (C36 H52 N2 Na1 O6 P3)n,(C34 H47 N3 Na1 O5 P3)n

Unit Cell Parameters: a 27.6426(13) b 11.7291(6) c 24.5253(12) P21/c

---

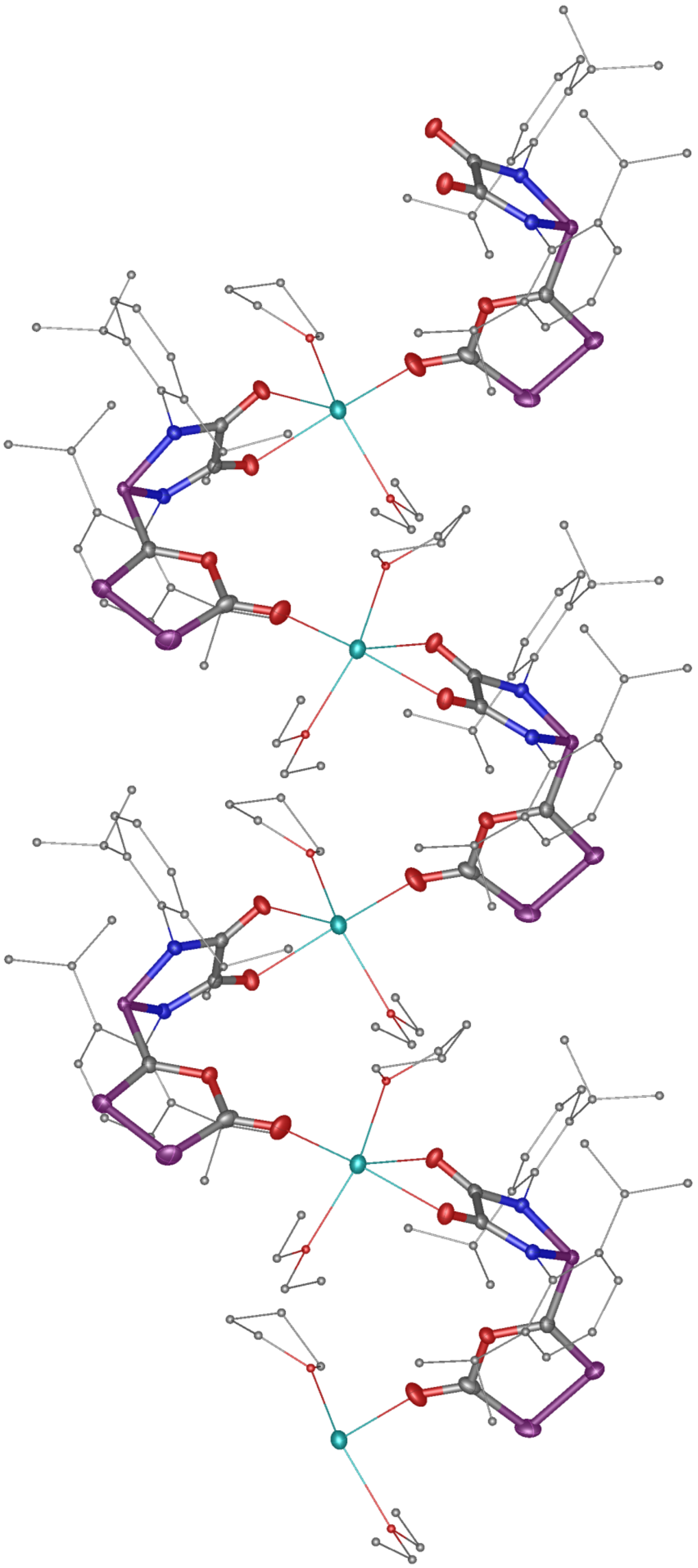

Structure of **10'** in the solid state. All hydrogen atoms omitted for clarity.

4. Computational part

Calculations were carried out with the Gaussian 09 package[3] (version g09, rev.d01). Geometry optimizations were performed with the B3LYP[4] or M06-2X[5] functional. The def2-TZVP, 6-31G(d)[6] or cc-pVDZ basis sets were used for all the atoms. Frequency calculations at the same level of theory were performed to identify the number of imaginary frequencies (zero for local minimum and one for transition states) and provide the Gibbs free energy and frontier molecular orbitals (HOMO and LUMO). Dispersion correction calculations using the corresponding B3LYP-D functional were performed with the DFT-D3 program of Grimme.[7] Transition states were located by using the quadratic synchronous transit (QST3) algorithm developed by Schlegel et al.[8] The connection of all transition states with the related ground states was verified by following the intrinsic reaction coordinate (IRC).[9] Gibbs free reaction energies and enthalpies were calculated for standard conditions (p = 1 atm, T = 298 K) and are unscaled. Nucleus independent chemical shift was computed at the M06-2X/6-311++G(2d,p)//M06-2X/6-31G(d) level of theory in the gas-phase. When the environments at points 1 angstrom above and below the ring centers are not equivalent, the averaged values are used for NICS(1). Optimized structures were visualized using the CYLview program.[10]

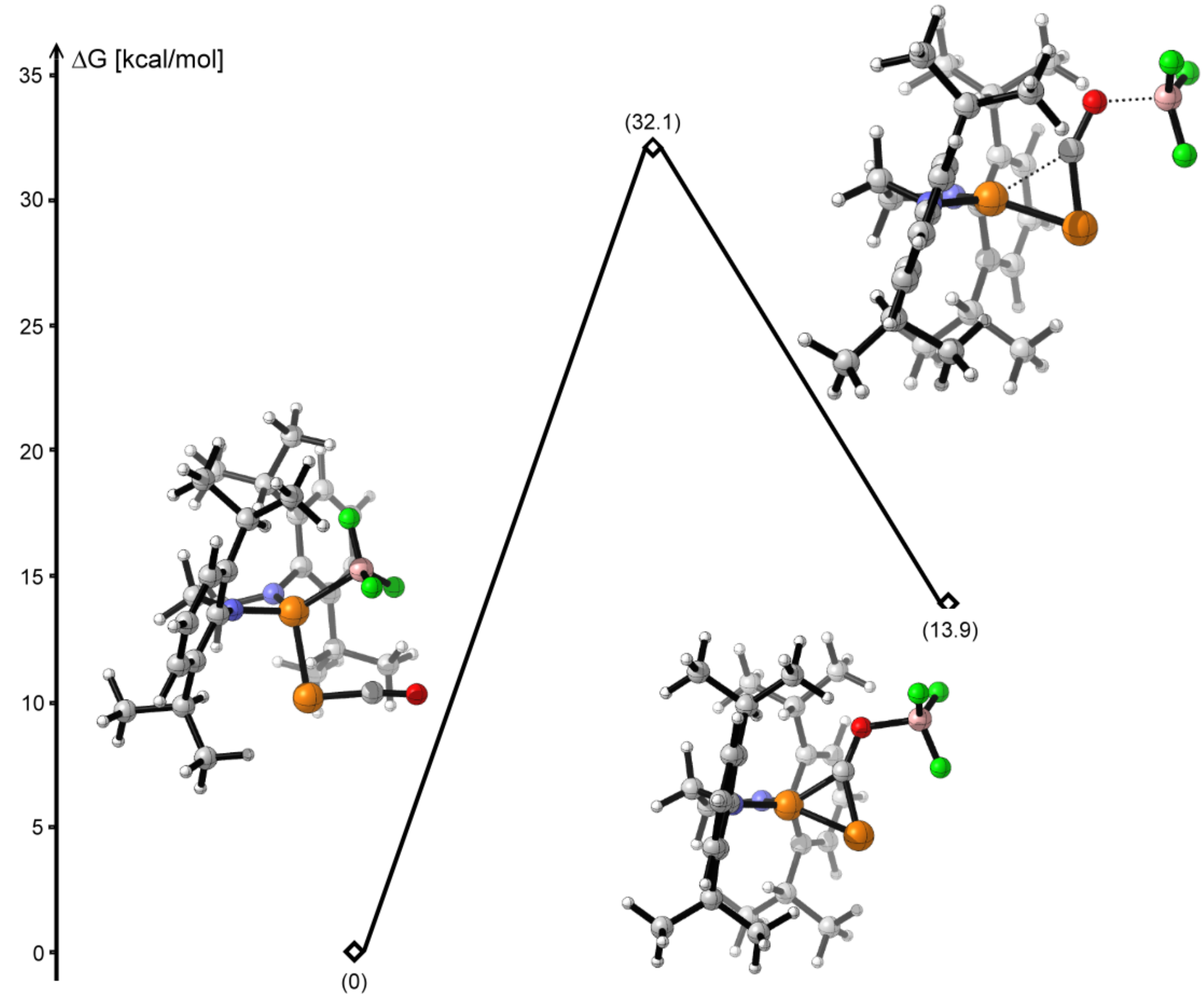

Figure S1. Energy profile for the BF_3 assisted cyclization calculated at the B3LYP-D3BJ/cc-pVDZ level of theory.

Comment to Figure S1: Even though the activation barrier of Lewis-acid activation is fairly high ($\Delta G^{\ddagger}$ = 32.1 kcal/mol) it should be pointed out that the in reference (starting) molecule BF_3 is coordinated to the inner phosphorus of the [**P**]-PCO molecule. As this reaction also only proceeds when employing a bulky Lewis acidic borane such as $B(C_6F_5)_3$, in which this coordination is inhibited based on sterics, it seems reasonable to assume that the energies of transition state and product will significantly decrease.

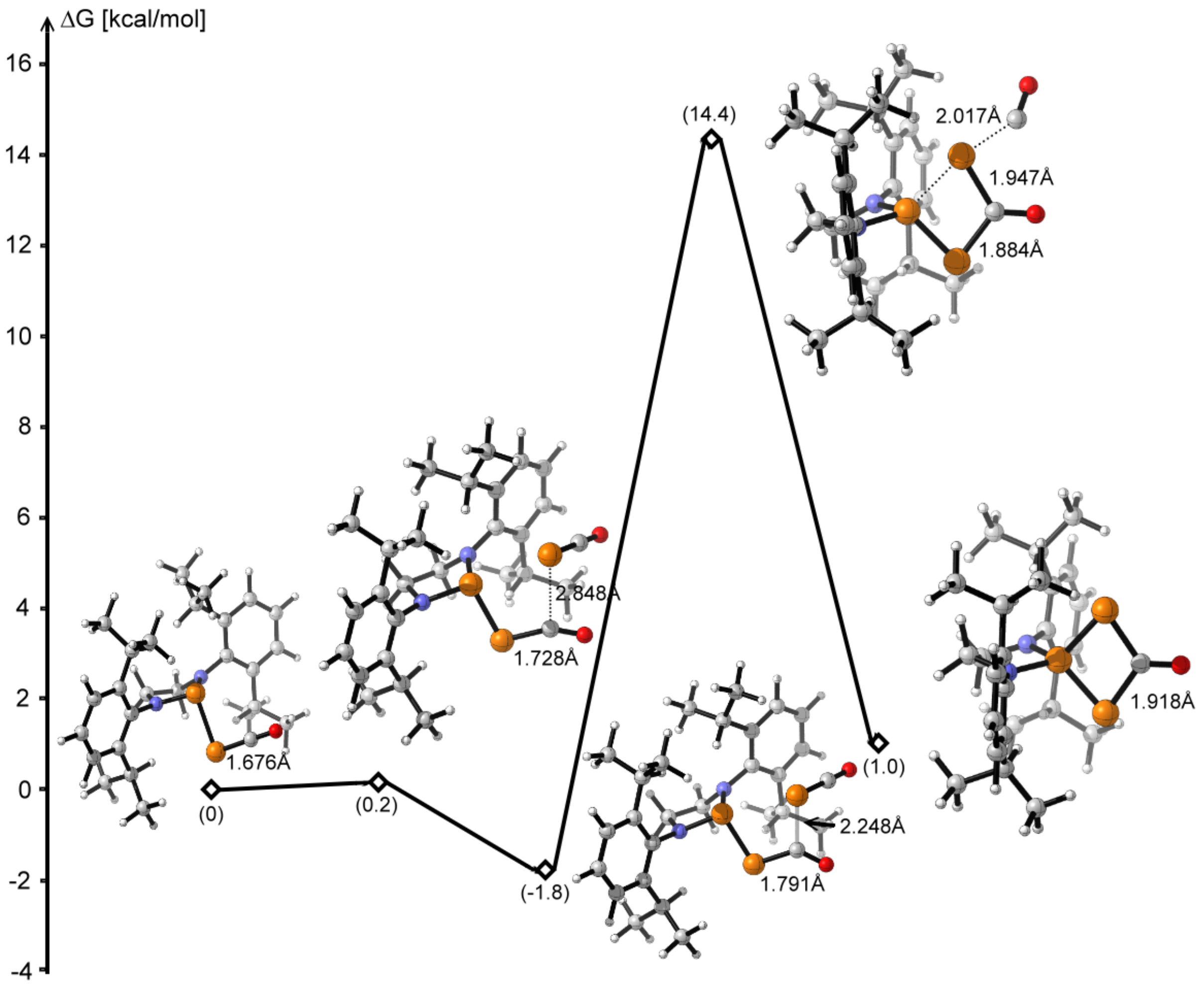

Figure S2. Energy profile for the PCO- addition to [P]-PCO calculated at the B3LYP-D3BJ/cc-pVDZ level of theory.

Comment to Figure S2: The reaction pathway shows a low lying transition state ($\Delta G^{\ddagger}$ = 16.2 kcal/mol) for the attack of the endocyclic phosphorus onto the P-atom releasing CO in agreement with our recent proposed P-CO substitution approach. Even though the reaction is predicted to be slightly endergonic, the calculations do not take into account the sodium cation as well as any solvent effects. As the latter play a crucial role in solubilizing NaPCO and therefore increasing its reactivity, the relative stability of starting materials and products might strongly vary. However, the point of this calculation is to rationalize the observed reactivity and to give an energetically reasonable mechanism for the formation of the 4-membered "P^{-}" insertion product.

Optimized coordinates:

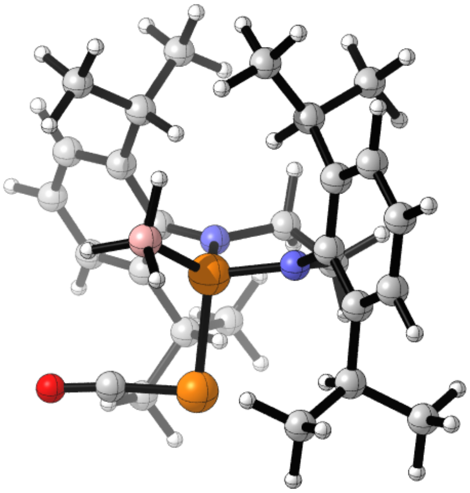

Zero-point correction=	0.630854 (Hartree/Particle)
Thermal correction to Energy=	0.667177
Thermal correction to Enthalpy=	0.668121
Thermal correction to Gibbs Free Energy=	0.564066
Sum of electronic and zero-point Energies=	-1945.948439
Sum of electronic and thermal Energies=	-1945.912117
Sum of electronic and thermal Enthalpies=	-1945.911172
Sum of electronic and thermal Free Energies=	-1946.015227

C	3.006612	1.512458	-0.203310
C	2.686193	0.142326	-0.246610
C	3.668671	-0.841649	-0.063796
C	4.978156	-0.433642	0.184299
C	5.309146	0.909574	0.234743
C	4.330331	1.870906	0.036808
N	1.337120	-0.250924	-0.525854
C	0.837125	-0.337082	-1.901330
C	-0.550598	0.307422	-1.885593
N	-1.069096	0.107976	-0.526523
P	0.093093	-0.402060	0.573006
B	0.317841	0.402439	2.282028
C	3.346258	-2.320216	-0.121926
C	3.500437	-2.973666	1.255742
C	1.971833	2.592669	-0.450980
C	2.007350	3.701256	0.604457
C	-2.452121	0.344663	-0.239196
C	-2.832661	1.567634	0.345642
C	-4.181482	1.778067	0.622279
C	-5.134609	0.821662	0.314590
C	-4.751512	-0.361321	-0.294544
C	-3.414332	-0.623118	-0.585447
C	-1.839290	2.683589	0.602990
C	-1.953802	3.755577	-0.490538
C	-3.057407	-1.906447	-1.310403
C	-3.637257	-3.149176	-0.627415
P	-0.126564	-2.657783	0.782798
C	-1.238432	-2.522005	2.016319
O	-2.017961	-2.525876	2.867901
C	-3.511509	-1.847986	-2.776871
C	-1.976629	3.301015	1.995902
C	4.179613	-3.048459	-1.182325

C	2.118726	3.182441	-1.860663	H	-2.921986	3.834379	2.113074
H	-0.492520	1.376816	-2.109346	H	-1.911307	2.535884	2.767849
H	-1.206957	-0.158199	-2.619269	H	-1.201323	4.533205	-0.343745
H	1.520054	0.184331	-2.567672	H	-1.812057	3.327278	-1.484351
H	0.776505	-1.381582	-2.227581	H	-2.937100	4.230278	-0.471936
H	-5.502960	-1.096968	-0.548120	H	1.330054	3.912810	-2.053060
H	-6.177723	1.002255	0.541136	H	3.080862	3.686501	-1.972000
H	-4.489483	2.709024	1.078531	H	2.062061	2.410395	-2.629515
H	4.599016	2.917989	0.074997	H	1.178620	4.393578	0.445365
H	6.331168	1.209501	0.428347	H	1.917407	3.286586	1.607321
H	5.747352	-1.178735	0.338617	H	2.931021	4.280099	0.551676
H	-0.841976	2.261055	0.543887	H	2.301645	-2.415587	-0.415646
H	-1.973613	-2.006705	-1.292723	H	3.196581	-4.021415	1.217614
H	0.991452	2.127327	-0.390564	H	4.538641	-2.933740	1.592130
H	-3.244797	-4.052925	-1.096900	H	2.883534	-2.469052	1.999127
H	-3.382734	-3.177882	0.430451	H	3.871166	-4.093438	-1.251944
H	-4.725104	-3.179237	-0.711402	H	4.056964	-2.589410	-2.164947
H	-3.184200	-2.740135	-3.315030	H	5.243179	-3.033105	-0.937566
H	-4.600055	-1.795633	-2.844119	H	0.571851	1.560541	2.100200
H	-3.111533	-0.972803	-3.291035	H	-0.734456	0.200551	2.835954
H	-1.171057	4.017615	2.164695	H	1.221328	-0.247296	2.746763

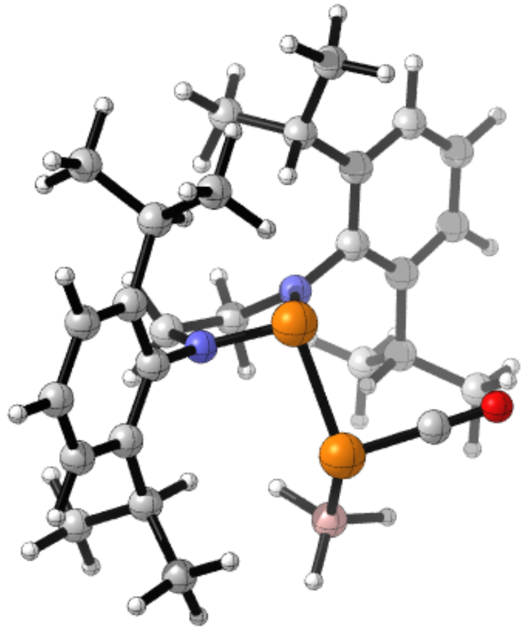

Zero-point correction=	0.630117 (Hartree/Particle)
Thermal correction to Energy=	0.667906
Thermal correction to Enthalpy=	0.668850
Thermal correction to Gibbs Free Energy=	0.560231
Sum of electronic and zero-point Energies=	-1945.928067
Sum of electronic and thermal Energies=	-1945.890278
Sum of electronic and thermal Enthalpies=	-1945.889334
Sum of electronic and thermal Free Energies=	-1945.997953

C	-3.056588	1.552192	0.213596
C	-2.645717	0.254649	-0.146481
C	-3.570645	-0.802160	-0.228716
C	-4.902159	-0.541174	0.088001
C	-5.312637	0.722126	0.480753
C	-4.395300	1.758370	0.538372
N	-1.270741	0.013130	-0.461674
P	-0.033961	0.028447	0.680056
P	-0.290890	-2.308199	1.369443
C	1.024418	-2.225124	2.402567
O	1.894892	-2.136818	3.152546
C	-3.171158	-2.193389	-0.677215
C	-3.716196	-2.494237	-2.080479
C	-2.097602	2.726301	0.240945
C	-1.836458	3.208053	1.672772
C	-0.825441	0.044252	-1.859538
C	0.660219	-0.305828	-1.837686
N	1.139182	0.106721	-0.508310
C	2.505094	0.482729	-0.286494
C	3.524129	-0.480350	-0.365179
C	4.845971	-0.059336	-0.217871
C	5.152613	1.265926	0.028811
C	4.135121	2.203304	0.129444
C	2.801790	1.839167	-0.031547
C	3.234657	-1.946097	-0.614342
C	3.565843	-2.341688	-2.059924
C	1.712488	2.897152	0.005844
C	1.344216	3.337246	-1.417421
C	-3.614200	-3.274762	0.313556
C	-2.579036	3.878224	-0.648710
C	2.053251	4.112634	0.868816
C	3.976452	-2.860379	0.366798
H	-0.998068	1.044878	-2.270732
H	-1.386765	-0.672013	-2.459648
H	1.215996	0.232920	-2.604144
H	0.803729	-1.375438	-1.989546
H	-5.626253	-1.342962	0.033159
H	-6.349693	0.901135	0.734541
H	-4.725565	2.746493	0.830814
H	4.387012	3.234817	0.328427
H	6.183935	1.572044	0.149717
H	5.642676	-0.787652	-0.284091
H	-1.150212	2.386362	-0.171618
H	-2.085066	-2.230328	-0.738581
H	0.828229	2.445176	0.452768
H	-3.208156	-4.241153	0.015252
H	-3.258477	-3.057188	1.320897
H	-4.701757	-3.361099	0.350099
H	-3.356433	-3.465142	-2.425391
H	-4.807979	-2.517847	-2.077548
H	-3.405790	-1.737738	-2.803187
H	-1.114916	4.027286	1.676685
H	-2.756518	3.568766	2.136664
H	-1.440055	2.403426	2.292512
H	-1.832605	4.674605	-0.670734
H	-2.749066	3.541010	-1.672288
H	-3.510510	4.310388	-0.279633
H	0.533146	4.067555	-1.399247

H	2.205376	3.796775	-1.906757	H	3.815979	-2.551373	1.399110
H	1.025847	2.493051	-2.027515	H	3.277382	-3.378481	-2.240313
H	1.178497	4.759524	0.951654	H	3.045013	-1.712750	-2.782649
H	2.352772	3.817201	1.875053	H	4.637232	-2.246960	-2.250666
H	2.856158	4.711294	0.435307	B	0.161229	-3.838663	0.026949
H	2.171635	-2.111609	-0.460072	H	1.268377	-4.224978	0.269903
H	3.615675	-3.883242	0.258225	H	-0.723413	-4.614641	0.268454
H	5.051897	-2.863640	0.179629	H	0.040394	-3.260099	-1.024253

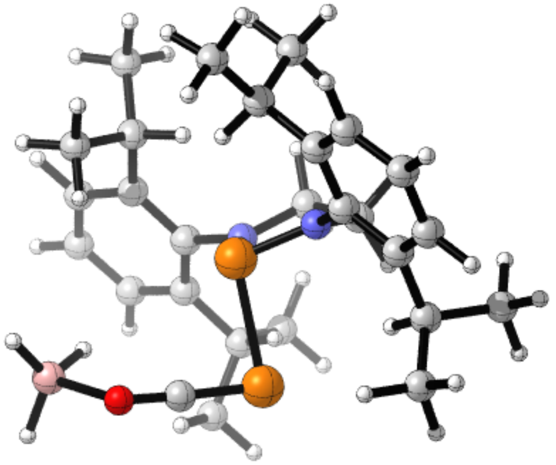

Zero-point correction=	0.629296 (Hartree/Particle)
Thermal correction to Energy=	0.667888
Thermal correction to Enthalpy=	0.668832
Thermal correction to Gibbs Free Energy=	0.555479
Sum of electronic and zero-point Energies=	-1945.910639
Sum of electronic and thermal Energies=	-1945.872047
Sum of electronic and thermal Enthalpies=	-1945.871103
Sum of electronic and thermal Free Energies=	-1945.984457

C	-2.781846	1.611192	-0.223848	P	0.101472	-0.040623	0.517266
C	-2.396134	0.302858	-0.565321	P	-0.022428	-2.424144	1.194683
C	-3.348742	-0.713684	-0.748329	C	-0.726118	-1.840563	2.534759
C	-4.693393	-0.396986	-0.577800	O	-1.232022	-1.365994	3.493622
C	-5.087610	0.881471	-0.219131	C	-2.956126	-2.129825	-1.119236
C	-4.137663	1.873555	-0.043189	C	-3.383696	-2.468546	-2.553862
N	-1.008170	0.000370	-0.741300	C	-1.776070	2.731161	-0.039020

C	-1.660583	3.134788	1.436115
C	-0.432113	-0.019589	-2.089424
C	1.006554	-0.494000	-1.913423
N	1.388005	-0.072932	-0.561250
C	2.750996	0.169112	-0.198922
C	3.670247	-0.889858	-0.158035
C	5.004472	-0.600519	0.127763
C	5.411479	0.692679	0.399756
C	4.485306	1.726272	0.391472
C	3.147606	1.492771	0.088808
C	3.262239	-2.328589	-0.409484
C	3.832520	-2.847245	-1.735477
C	2.156453	2.642698	0.026865
C	1.940948	3.095602	-1.423595
C	-3.515890	-3.155087	-0.127611
C	-2.093081	3.942495	-0.922961
C	2.529379	3.835050	0.908164
C	3.650729	-3.244209	0.756129
H	-0.474956	0.987167	-2.519151
H	-0.992858	-0.689900	-2.741401
H	1.680229	-0.044689	-2.642102
H	1.065637	-1.581669	-2.022843
H	-5.441201	-1.167779	-0.708187
H	-6.136797	1.103984	-0.072867
H	-4.453495	2.869350	0.238062
H	4.814469	2.729718	0.619247
H	6.449068	0.898863	0.629222
H	5.728163	-1.404429	0.151384
H	-0.804102	2.358143	-0.357117
H	-1.869636	-2.193098	-1.066676
H	1.202253	2.274114	0.402809
H	-3.127491	-4.149832	-0.354840
H	-3.236957	-2.903397	0.894207
H	-4.605069	-3.203693	-0.178143
H	-3.037244	-3.466338	-2.831390
H	-4.471166	-2.453023	-2.650832
H	-2.981209	-1.753893	-3.274133
H	-0.876829	3.884007	1.568121
H	-2.598627	3.561792	1.795829
H	-1.427196	2.275870	2.064827
H	-1.311347	4.698277	-0.821753
H	-2.158905	3.657313	-1.974237
H	-3.039107	4.408041	-0.642663
H	1.183549	3.880216	-1.473098
H	2.869249	3.491322	-1.840970
H	1.614562	2.271568	-2.056542
H	1.709482	4.554929	0.913899
H	2.717090	3.528292	1.937766
H	3.414697	4.356181	0.539218
H	2.177082	-2.357980	-0.477774
H	3.272727	-4.253450	0.583119
H	4.733682	-3.309556	0.872863
H	3.228193	-2.881754	1.693363
H	3.486140	-3.864145	-1.930408
H	3.531105	-2.216311	-2.573150
H	4.923868	-2.863776	-1.710913
B	-2.923429	-0.610089	3.487578
H	-3.515606	-1.446607	4.102798
H	-2.662513	0.410746	4.051815
H	-3.109321	-0.554772	2.307396

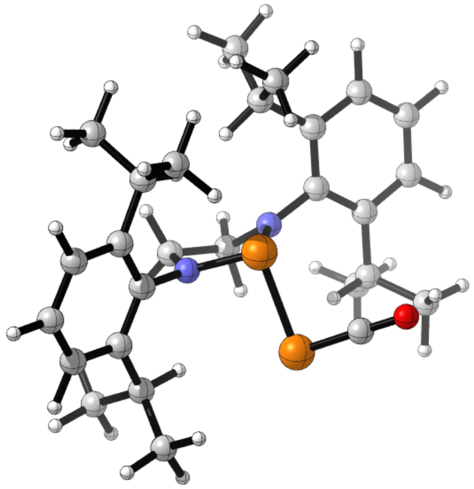

Zero-point correction=	0.596557 (Hartree/Particle)
Thermal correction to Energy=	0.631237
Thermal correction to Enthalpy=	0.632181
Thermal correction to Gibbs Free Energy=	0.530102
Sum of electronic and zero-point Energies=	-1918.870809
Sum of electronic and thermal Energies=	-1918.836130
Sum of electronic and thermal Enthalpies=	-1918.835186
Sum of electronic and thermal Free Energies=	-1918.937265

C	3.515427	-0.726089	-0.319907	C	3.139279	-2.192791	-0.452964
C	2.545097	0.298734	-0.386966	C	3.707241	-3.046168	0.689815
C	2.913952	1.661889	-0.290618	C	-2.641701	0.186327	-0.187211
C	4.265914	1.974436	-0.099177	C	-3.584552	-0.851770	-0.378317
C	5.232611	0.973240	-0.030122	C	-4.931557	-0.594436	-0.095637
C	4.857789	-0.363407	-0.148494	C	-5.341925	0.651183	0.375420
N	1.163430	-0.023734	-0.580396	C	-4.405063	1.665093	0.560449
C	0.632846	-0.463625	-1.874313	C	-3.048313	1.459913	0.275838
C	-0.806737	0.043238	-1.889438	C	-3.174521	-2.222000	-0.896718
N	-1.269875	-0.063512	-0.501003	C	-3.603727	-3.348695	0.054311
P	-0.001481	-0.090802	0.680959	C	-2.069361	2.609015	0.469925
P	-0.227710	-2.417977	1.232795	C	-2.430945	3.817499	-0.407345
C	0.751954	-2.213795	2.577526	C	-1.963603	3.001315	1.951267
O	1.436793	-2.088636	3.516140	C	-3.721872	-2.463279	-2.313509
C	1.887779	2.781258	-0.381307	C	2.278496	3.850360	-1.412041
C	1.635551	3.406608	0.999374	C	3.568952	-2.743319	-1.822528

H	-0.853304	1.095806	-2.230866	H	-1.238023	3.818304	2.086881
H	-1.439297	-0.560686	-2.558009	H	-2.935347	3.343405	2.343086
H	1.233099	-0.032810	-2.687903	H	-1.633935	2.146330	2.561097
H	0.656890	-1.566428	-1.965332	H	-1.665643	4.604923	-0.309554
H	-5.670004	-1.385393	-0.239751	H	-2.497012	3.531990	-1.469071
H	-6.395661	0.832398	0.598198	H	-3.399410	4.256121	-0.116878
H	-4.733418	2.640217	0.925644	H	1.467607	4.589047	-1.519799
H	4.567131	3.019752	-0.010602	H	3.185627	4.399556	-1.111929
H	6.282756	1.236297	0.114336	H	2.469003	3.402519	-2.400036
H	5.619791	-1.143109	-0.097584	H	0.877302	4.203542	0.932559
H	-1.077228	2.265628	0.148445	H	1.278088	2.651901	1.716163
H	-2.076539	-2.234540	-0.950229	H	2.559620	3.850278	1.405187
H	0.945507	2.330451	-0.721581	H	2.046169	-2.261106	-0.383624
H	-3.218900	-4.317423	-0.303126	H	3.315630	-4.073727	0.624173
H	-3.212581	-3.180489	1.068376	H	4.807234	-3.106568	0.648850
H	-4.701325	-3.428531	0.116515	H	3.426182	-2.637876	1.671596
H	-3.364247	-3.427521	-2.710092	H	3.250554	-3.792429	-1.936852
H	-4.824111	-2.487826	-2.312798	H	3.128639	-2.159488	-2.646371
H	-3.411281	-1.668013	-3.009662	H	4.665016	-2.707407	-1.938959

Transition state for the interconversion of $\mathbf{1}^{\mathbf{Dipp}}$ to **1***

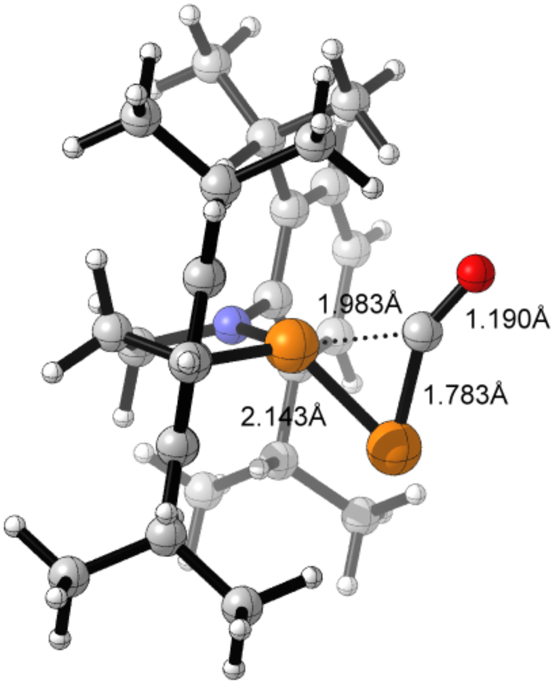

Imaginary frequency -111 (Int. 24)

Zero-point correction=	0.596209 (Hartree/Particle)
Thermal correction to Energy=	0.630878
Thermal correction to Enthalpy=	0.631822
Thermal correction to Gibbs Free Energy=	0.528844
Sum of electronic and zero-point Energies=	-1918.835491
Sum of electronic and thermal Energies=	-1918.800822
Sum of electronic and thermal Enthalpies=	-1918.799878
Sum of electronic and thermal Free Energies=	-1918.902856

C	3.408644	-1.183593	-0.161594
C	2.606252	-0.045685	-0.395639
C	3.141640	1.261208	-0.325052
C	4.500974	1.404390	-0.019077
C	5.309034	0.289404	0.197929
C	4.766745	-0.992503	0.123505
N	1.217985	-0.200371	-0.689906
C	0.706695	-0.564098	-2.012839
C	-0.735614	-0.049788	-2.037230
N	-1.247577	-0.276080	-0.682457
P	-0.002940	-0.155927	0.518309
P	-0.084108	-1.527301	2.163459
C	0.074333	0.223302	2.462888
O	0.160003	1.155929	3.197371
C	2.274298	2.491526	-0.546200
C	1.998110	3.216488	0.781383
C	2.832023	-2.589481	-0.230211
C	3.154594	-3.411132	1.025435
C	-2.604175	0.053964	-0.358430
C	-3.537348	-1.002046	-0.269999
C	-4.869171	-0.691544	0.033174
C	-5.264503	0.626452	0.253281
C	-4.328998	1.657122	0.177540
C	-2.986708	1.395285	-0.126332
C	-3.122347	-2.447336	-0.495538
C	-3.390100	-3.311093	0.745664
C	-1.997384	2.551646	-0.197809
C	-2.349528	3.512817	-1.344411
C	-1.890383	3.294160	1.142924
C	-3.799469	-3.033307	-1.744184
C	2.876173	3.447709	-1.585932
C	3.304471	-3.310255	-1.503665
H	-0.763596	1.020218	-2.318372
H	-1.351442	-0.610131	-2.758161
H	1.319396	-0.083202	-2.789812
H	0.731519	-1.658318	-2.178021
H	-5.605562	-1.494523	0.102551
H	-6.306426	0.852215	0.490724
H	-4.646290	2.685615	0.359914
H	4.934029	2.403978	0.050340
H	6.367957	0.420758	0.431137
H	5.406600	-1.858825	0.301077
H	-1.001855	2.138758	-0.411353
H	-2.037421	-2.440439	-0.669609
H	1.310170	2.140739	-0.939974
H	-3.032349	-4.340992	0.582731
H	-2.873135	-2.902844	1.627843
H	-4.466665	-3.365180	0.977558
H	-3.446331	-4.060569	-1.931715
H	-4.895249	-3.072416	-1.626603
H	-3.582861	-2.426573	-2.638048
H	-1.156158	4.111846	1.066272
H	-2.856078	3.737438	1.435294
H	-1.560132	2.622971	1.948487
H	-1.589968	4.306913	-1.430577
H	-2.406320	2.983337	-2.308816
H	-3.324371	3.998203	-1.173989
H	2.178434	4.276005	-1.789927

H	3.820442	3.893873	-1.233516	H	2.656994	-4.393249	0.976794
H	3.083412	2.930096	-2.535931	H	4.236711	-3.593673	1.126494
H	1.345175	4.088810	0.614017	H	2.806117	-2.895620	1.932718
H	1.505929	2.559979	1.513748	H	2.843904	-4.308697	-1.581620
H	2.936972	3.580207	1.231233	H	3.044235	-2.738427	-2.408372
H	1.738328	-2.491680	-0.283091	H	4.399161	-3.442133	-1.497853

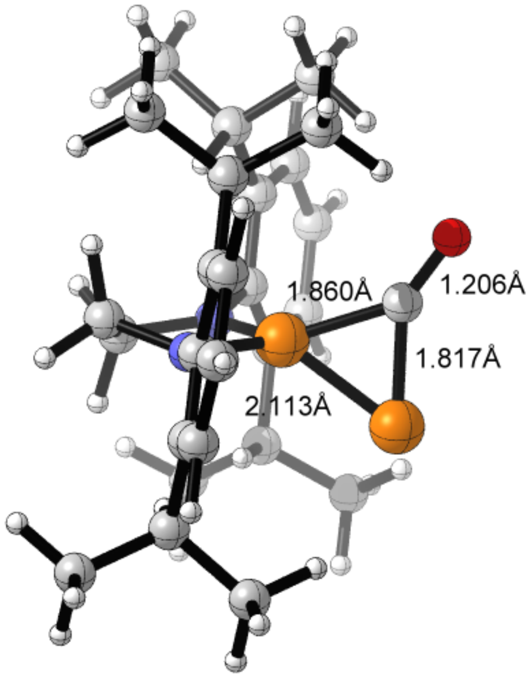

Zero-point correction=	0.596991 (Hartree/Particle)
Thermal correction to Energy=	0.631289
Thermal correction to Enthalpy=	0.632234
Thermal correction to Gibbs Free Energy=	0.531270
Sum of electronic and zero-point Energies=	-1918.835217
Sum of electronic and thermal Energies=	-1918.800920
Sum of electronic and thermal Enthalpies=	-1918.799975
Sum of electronic and thermal Free Energies=	-1918.900939

C	-2.994431	1.340047	-0.025592	C	-4.337584	1.568031	0.300424
C	-2.600517	0.017119	-0.333761	N	-1.240262	-0.275647	-0.680285
C	-3.519873	-1.053493	-0.299712	P	-0.002603	-0.281316	0.514946
C	-4.852073	-0.776101	0.032833	C	0.117705	0.355604	2.258640
C	-5.259769	0.522981	0.328492	O	0.256558	1.345618	2.932568

C	-3.092185	-2.479905	-0.607801
C	-3.830114	-3.034652	-1.835976
C	-2.022003	2.512641	-0.061448
C	-2.004405	3.301196	1.256297
C	-0.725522	0.031110	-2.019275
C	0.713521	-0.499485	-2.033694
N	1.222135	-0.301280	-0.674693
C	2.599209	-0.060299	-0.380379
C	3.452789	-1.150220	-0.108805
C	4.796385	-0.883272	0.184254
C	5.275202	0.425378	0.218306
C	4.416978	1.492143	-0.045149
C	3.067911	1.273105	-0.350716
C	2.946906	-2.583773	-0.143043
C	3.540491	-3.345703	-1.338987
C	2.151487	2.453753	-0.636820
C	2.653624	3.280793	-1.830376
P	-0.134205	-1.444231	2.274485
C	1.949166	3.329282	0.610118
C	3.214380	-3.316670	1.179003
C	-3.271541	-3.393248	0.614263
C	-2.327439	3.435270	-1.253721
H	-0.743758	1.116579	-2.227351
H	-1.349059	-0.473099	-2.773444
H	1.337511	0.059564	-2.746304
H	0.736047	-1.568778	-2.317449
H	-5.578569	-1.590317	0.064798
H	-6.301829	0.722925	0.587402
H	-4.666169	2.580701	0.540329
H	4.800125	2.513786	-0.010602
H	6.324306	0.615918	0.454970

H	5.475178	-1.711553	0.395562
H	-1.007819	2.113780	-0.203952
H	-2.019621	-2.447572	-0.844619
H	1.168714	2.047531	-0.912148
H	-2.911702	-4.411142	0.391489
H	-2.709046	-3.009892	1.479650
H	-4.331605	-3.469581	0.907717
H	-3.465526	-4.045099	-2.082870
H	-4.915567	-3.106683	-1.656498
H	-3.680620	-2.390036	-2.716941
H	-1.264083	4.114574	1.195844
H	-2.984660	3.757901	1.467996
H	-1.722311	2.660680	2.102776
H	-1.577965	4.240643	-1.322436
H	-2.327311	2.880758	-2.205515
H	-3.318739	3.905073	-1.144685
H	1.937437	4.083828	-2.068969
H	3.624765	3.755177	-1.614252
H	2.781077	2.654546	-2.728031
H	1.265887	4.163870	0.380748
H	1.517814	2.756397	1.444157
H	2.904147	3.763910	0.949110
H	1.856994	-2.541574	-0.280177
H	2.782740	-4.330263	1.149279
H	4.294064	-3.420560	1.375245
H	2.761913	-2.776107	2.023781
H	3.133366	-4.368773	-1.389703
H	3.313291	-2.836748	-2.289370
H	4.637336	-3.423402	-1.256950

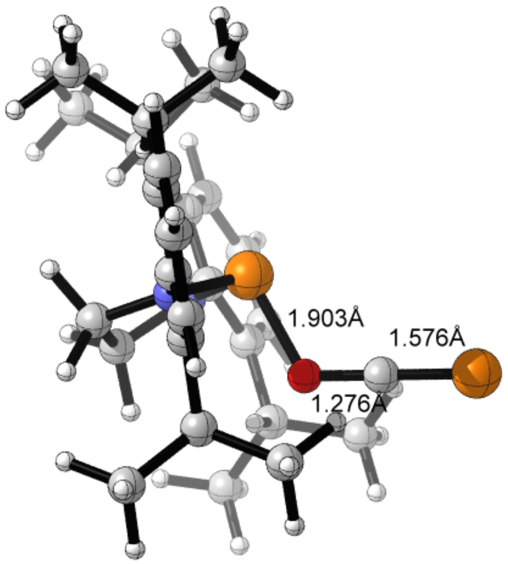

Zero-point correction=	0.597124 (Hartree/Particle)
Thermal correction to Energy=	0.631581
Thermal correction to Enthalpy=	0.632525
Thermal correction to Gibbs Free Energy=	0.531045
Sum of electronic and zero-point Energies=	-1918.846021
Sum of electronic and thermal Energies=	-1918.811564
Sum of electronic and thermal Enthalpies=	-1918.810620
Sum of electronic and thermal Free Energies=	-1918.912100

C	-2.989885	1.639268	0.215151	N	1.242115	0.143207	-0.620303
C	-2.556965	0.404766	-0.318731	C	2.639715	0.281072	-0.318344
C	-3.463336	-0.646779	-0.578345	C	3.478183	-0.852668	-0.287751
C	-4.819483	-0.434827	-0.298557	C	4.835158	-0.660821	0.009297
C	-5.263799	0.775974	0.229298	C	5.342976	0.609933	0.266248
C	-4.354758	1.800353	0.487326	C	4.502245	1.721717	0.218628
N	-1.169775	0.215418	-0.629093	C	3.142240	1.581956	-0.082056
P	0.026774	-0.082985	0.536288	C	2.960084	-2.253903	-0.573977
O	-0.097182	-1.981662	0.529969	C	3.588670	-2.820066	-1.857468
C	-3.004009	-1.982897	-1.141640	C	2.232292	2.801803	-0.115413
C	-3.590513	-2.225113	-2.541581	C	2.867391	4.004183	-0.826391
C	-2.020254	2.768496	0.533000	C	1.785867	3.178468	1.307244
C	-1.882662	2.949969	2.053328	C	3.180688	-3.193851	0.620774
C	-0.680085	0.488538	-1.989056	C	-3.340081	-3.139723	-0.188210
C	0.773143	0.012009	-2.010403	C	-2.414259	4.083856	-0.154410

H	-0.754781	1.571962	-2.199398	H	-2.849333	3.223478	2.506686
H	-1.290473	-0.046672	-2.732093	H	-1.537285	2.020806	2.532227
H	1.400266	0.632670	-2.666100	H	-1.651194	4.856790	0.032936
H	0.845432	-1.036216	-2.351173	H	-2.510755	3.952105	-1.243635
H	-5.536594	-1.235372	-0.489225	H	-3.374966	4.470398	0.222781
H	-6.324409	0.920715	0.446086	H	2.129013	4.815289	-0.930729
H	-4.710702	2.742040	0.909411	H	3.720788	4.412759	-0.261850
H	4.911475	2.713148	0.417704	H	3.226371	3.734006	-1.831748
H	6.401678	0.737376	0.502401	H	1.102399	4.042839	1.285845
H	5.501718	-1.524357	0.044015	H	1.265701	2.343146	1.800421
H	-1.032510	2.483581	0.143257	H	2.655949	3.444916	1.929554
H	-1.910637	-1.947090	-1.226498	H	1.877214	-2.190013	-0.728635
H	1.330915	2.522533	-0.680470	H	2.736017	-4.180398	0.416186
H	-2.937713	-4.086626	-0.582535	H	4.253641	-3.342702	0.826743
H	-2.901712	-2.978151	0.807434	H	2.705964	-2.800700	1.531704
H	-4.429598	-3.262040	-0.069760	H	3.166387	-3.811758	-2.086814
H	-3.201601	-3.164856	-2.966157	H	3.403715	-2.159605	-2.720065
H	-4.689853	-2.303083	-2.508439	H	4.680326	-2.936158	-1.755536
H	-3.338551	-1.404945	-3.233431	C	-0.220419	-2.594258	1.642005
H	-1.159463	3.746829	2.287871	P	-0.378149	-3.344912	3.018863

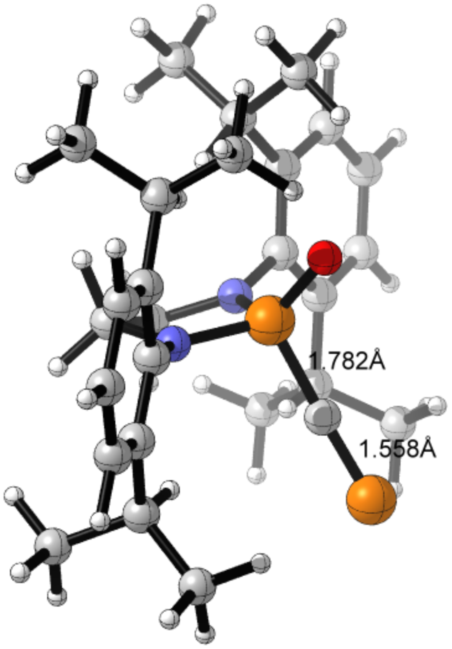

Zero-point correction= 0.597811 (Hartree/Particle)

Thermal correction to Energy=	0.631867
Thermal correction to Enthalpy=	0.632811
Thermal correction to Gibbs Free Energy=	0.533273
Sum of electronic and zero-point Energies=	-1918.851156
Sum of electronic and thermal Energies=	-1918.817101
Sum of electronic and thermal Enthalpies=	-1918.816156
Sum of electronic and thermal Free Energies=	-1918.915694

C	-3.509926	-0.877995	-0.285042
C	-2.592976	0.194563	-0.203994
C	-3.013137	1.499082	0.141109
C	-4.372687	1.702826	0.414422
C	-5.288338	0.655520	0.343226
C	-4.858612	-0.623134	-0.007399
N	-1.215070	-0.059778	-0.513046
C	-0.704355	0.204589	-1.863146
C	0.738850	-0.314888	-1.868447
N	1.239045	-0.105567	-0.508083
P	0.014374	-0.153906	0.656893
O	0.092561	0.824852	1.798626
C	-0.087600	-1.809137	1.308847
P	-0.156694	-3.239723	1.921434
C	-2.055804	2.680567	0.206217
C	-1.993938	3.277799	1.619128
C	-3.069538	-2.274080	-0.697263
C	-3.544040	-3.349631	0.289649
C	2.619216	0.160265	-0.233366
C	3.526729	-0.915404	-0.125099
C	4.873764	-0.626958	0.126998
C	5.308024	0.689112	0.275950
C	4.400123	1.740068	0.161805
C	3.044876	1.501458	-0.102337
C	3.077239	-2.356751	-0.304708
C	3.597671	-2.928011	-1.633885
C	2.089414	2.676078	-0.250952
C	1.921614	3.433771	1.073966
C	2.523145	3.611131	-1.390302
C	3.486362	-3.242267	0.880205
C	-2.414573	3.742942	-0.844963
C	-3.542302	-2.594468	-2.125452
H	0.769016	-1.384196	-2.149353
H	1.365605	0.244116	-2.577954
H	-1.321605	-0.321493	-2.606239
H	-0.736405	1.286144	-2.092053
H	4.749307	2.767913	0.276600
H	6.360737	0.896979	0.479971
H	5.591582	-1.444983	0.211063
H	-5.582422	-1.438033	-0.065291
H	-6.343260	0.836763	0.560868
H	-4.718127	2.701519	0.687618
H	1.980132	-2.356335	-0.343974
H	1.105955	2.268004	-0.517203
H	-1.971715	-2.281592	-0.693586
H	1.216045	4.271263	0.945663
H	1.521826	2.761091	1.844601
H	2.881335	3.852652	1.419644
H	1.778251	4.411027	-1.532813
H	3.490679	4.093504	-1.175308
H	2.627501	3.065323	-2.342021
H	3.068232	-4.255363	0.762824
H	4.581330	-3.343003	0.957305
H	3.115400	-2.826565	1.828965
H	3.228708	-3.955322	-1.788346
H	3.270534	-2.313139	-2.487391
H	4.699773	-2.958465	-1.649567
H	-3.177132	-3.584445	-2.445060
H	-4.643359	-2.604434	-2.185924
H	-3.179032	-1.845519	-2.846997

H	-3.111062	-4.328117	0.025472	H	-2.975126	3.666009	1.938936
H	-3.238639	-3.105648	1.318031	H	-1.653791	2.517182	2.334477
H	-4.640750	-3.460841	0.278510	H	-1.674381	4.559610	-0.831738
H	-1.047526	2.315008	-0.020843	H	-2.435307	3.313962	-1.859953
H	-1.276037	4.113333	1.645637	H	-3.404999	4.186828	-0.651583

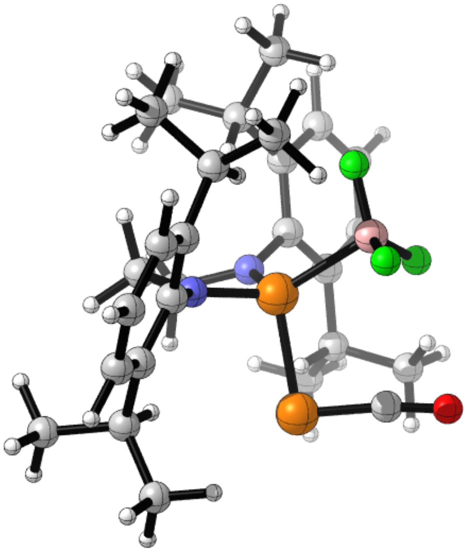

Zero-point correction=	0.610813 (Hartree/Particle)
Thermal correction to Energy=	0.650702
Thermal correction to Enthalpy=	0.651647
Thermal correction to Gibbs Free Energy=	0.538591
Sum of electronic and zero-point Energies=	-2243.449295
Sum of electronic and thermal Energies=	-2243.409406
Sum of electronic and thermal Enthalpies=	-2243.408462
Sum of electronic and thermal Free Energies=	-2243.521517

C	2.845955	1.583771	-0.724749	N	-1.299545	0.004905	-0.729657
C	2.514892	0.207340	-0.703843	P	-0.017874	-0.432986	0.320164
C	3.502197	-0.787282	-0.554818	B	0.567476	0.427617	2.215585
C	4.837671	-0.383906	-0.424031	C	3.153536	-2.265128	-0.514876
C	5.183874	0.964079	-0.450353	C	3.453010	-2.858373	0.870640
C	4.195307	1.935476	-0.600529	C	1.797050	2.670136	-0.913164
N	1.143109	-0.179265	-0.887552	C	1.991363	3.860482	0.035851
C	0.537266	-0.299170	-2.220389	C	-2.662551	0.220094	-0.338338
C	-0.844022	0.341674	-2.089304	C	-3.015494	1.405977	0.347442

C	-4.358420	1.593199	0.699260
C	-5.328081	0.648496	0.371731
C	-4.969036	-0.504176	-0.323118
C	-3.638259	-0.742320	-0.688559
C	-2.004026	2.501260	0.647443
C	-2.154489	3.655800	-0.358277
C	-3.296522	-1.995813	-1.479654
C	-3.788677	-3.273767	-0.785525
P	-0.469154	-2.661965	0.564850
C	0.174392	-2.778575	2.128320
O	0.563914	-2.983188	3.199057
C	-3.848850	-1.908504	-2.913163
C	-2.083720	3.008227	2.093003
C	3.858771	-3.050645	-1.630009
C	1.762231	3.152878	-2.373871
H	-0.789339	1.436667	-2.220568
H	-1.548131	-0.062991	-2.830326
H	1.168818	0.211147	-2.957307
H	0.451414	-1.362854	-2.514682
H	-5.735376	-1.235629	-0.585078
H	-6.369921	0.813958	0.654447
H	-4.648626	2.499983	1.231698
H	4.477103	2.989105	-0.614847
H	6.229652	1.262224	-0.349020
H	5.615579	-1.139031	-0.298825
H	-0.998761	2.088553	0.512934
H	-2.200385	-2.054129	-1.545931
H	0.820141	2.227225	-0.682438

H	-3.444809	-4.164376	-1.335778
H	-3.406068	-3.337204	0.243337
H	-4.889108	-3.313026	-0.745793
H	-3.541731	-2.787725	-3.502701
H	-4.950600	-1.873449	-2.909150
H	-3.493848	-1.004375	-3.432653
H	-1.254380	3.704560	2.288953
H	-3.026637	3.544698	2.287761
H	-1.998651	2.175656	2.804795
H	-1.386845	4.425715	-0.177676
H	-2.048605	3.302612	-1.396553
H	-3.144101	4.133565	-0.268456
H	0.960962	3.896409	-2.516718
H	2.718318	3.626309	-2.651771
H	1.588825	2.326168	-3.079672
H	1.133497	4.547253	-0.046882
H	2.062821	3.524519	1.077770
H	2.895047	4.439965	-0.213416
H	2.071697	-2.355282	-0.681410
H	3.088986	-3.896712	0.932874
H	4.537046	-2.867688	1.072735
H	2.970410	-2.269283	1.662704
H	3.542383	-4.106219	-1.613512
H	3.621506	-2.636408	-2.623050
H	4.954615	-3.030261	-1.512103
F	0.824413	1.754958	1.977039
F	-0.498008	0.171515	3.037544
F	1.687667	-0.339230	2.459689

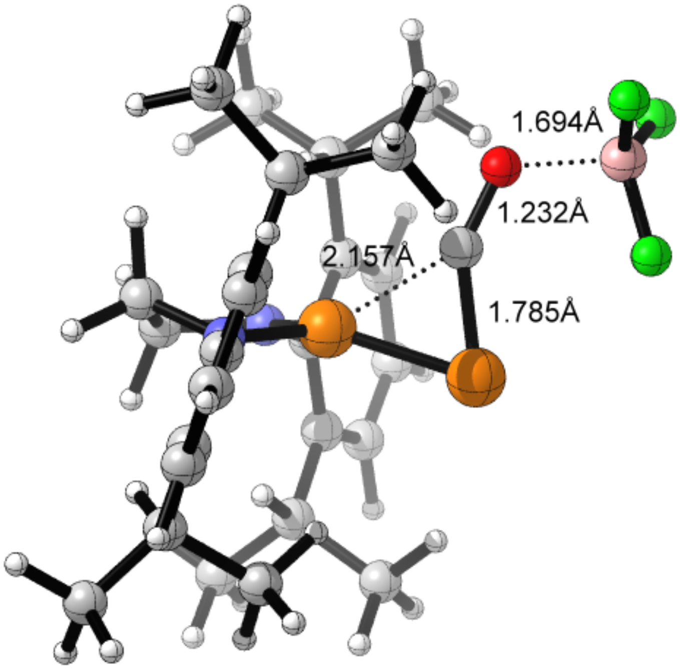

Imaginary frequency	-21 (Int. 5.6)
Zero-point correction=	0.609867 (Hartree/Particle)
Thermal correction to Energy=	0.649058
Thermal correction to Enthalpy=	0.650002
Thermal correction to Gibbs Free Energy=	0.535296
Sum of electronic and zero-point Energies=	-2243.395846
Sum of electronic and thermal Energies=	-2243.356655
Sum of electronic and thermal Enthalpies=	-2243.355711
Sum of electronic and thermal Free Energies=	-2243.470417

C	3.309599	0.913374	0.747311
C	2.796723	-0.378152	0.515636
C	3.587867	-1.412635	-0.037350
C	4.923190	-1.130037	-0.339554
C	5.452752	0.141885	-0.111309
C	4.653836	1.149549	0.419855
N	1.436266	-0.705245	0.834049
P	0.077800	-0.133274	0.006632
C	-0.456664	1.827581	-0.717192
O	-0.783699	2.978936	-0.425948
C	2.974885	-2.772917	-0.346415
C	3.970305	-3.934274	-0.246870
C	2.467269	2.045784	1.316579
C	2.442163	3.257368	0.370163
C	1.037625	-1.196352	2.163549
C	-0.337819	-1.851545	1.960672
N	-1.000909	-1.073799	0.907917
C	-2.360610	-1.287214	0.513011
C	-2.660586	-2.338448	-0.380453
C	-4.000231	-2.539059	-0.734339
C	-5.003337	-1.709591	-0.234287
C	-4.682650	-0.656307	0.620436
C	-3.357401	-0.418871	1.008045
C	-1.574392	-3.221289	-0.978554

C	-1.747761	-4.691424	-0.567490
C	-3.026126	0.731213	1.947832
C	-3.498423	0.409452	3.375996
P	-0.103093	0.653731	-2.014815
C	-3.597835	2.071618	1.462018
C	-1.515356	-3.070405	-2.506623
C	2.306162	-2.749857	-1.732503
C	2.945655	2.440057	2.722886
H	0.982534	-0.368107	2.893715
H	1.782442	-1.922640	2.517039
H	-0.934889	-1.817972	2.884132
H	-0.227664	-2.907745	1.653315
H	5.558347	-1.906283	-0.766494
H	6.496830	0.347202	-0.356901
H	5.075010	2.142722	0.582591
H	-5.473977	-0.002128	0.989310
H	-6.042795	-1.877839	-0.523788
H	-4.260691	-3.346970	-1.420688
H	1.432486	1.688097	1.412603
H	2.180644	-2.954351	0.393363
H	-1.932004	0.829819	1.973350
H	1.807927	-3.710420	-1.942144
H	1.552274	-1.952542	-1.805845
H	3.054811	-2.570431	-2.520973
H	3.440503	-4.894046	-0.352028
H	4.728079	-3.895095	-1.045623
H	4.496115	-3.935922	0.720516
H	1.707953	4.002952	0.707451
H	3.428704	3.746242	0.320242
H	2.162685	2.960041	-0.650887
H	2.305201	3.235386	3.136153
H	2.919347	1.580842	3.412569
H	3.980613	2.818758	2.701047
H	-3.211968	1.215425	4.070850
H	-4.595272	0.303670	3.415439
H	-3.061312	-0.533488	3.743159
H	-3.282355	2.880931	2.139155
H	-3.244553	2.330234	0.453964
H	-4.699907	2.063040	1.450176
H	-0.606524	-2.881999	-0.582893
H	-0.683334	-3.662303	-2.920504
H	-2.446556	-3.420735	-2.980384
H	-1.368471	-2.018601	-2.796844
H	-0.921148	-5.304032	-0.962583
H	-1.766216	-4.801274	0.528452
H	-2.689578	-5.108503	-0.959345
B	-1.037375	4.107358	-1.663583
F	-2.348132	4.444917	-1.493435
F	-0.120456	5.075906	-1.373430
F	-0.770075	3.384645	-2.813410

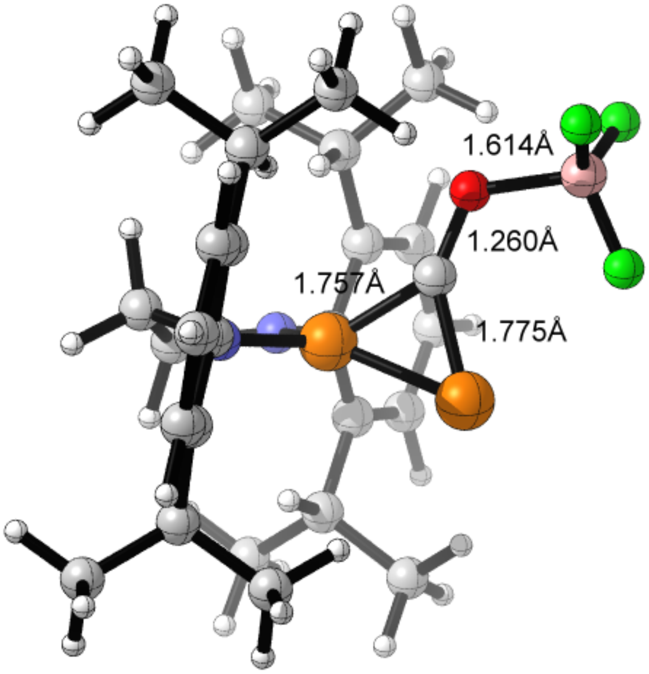

Zero-point correction=	0.612077 (Hartree/Particle)
Thermal correction to Energy=	0.650407
Thermal correction to Enthalpy=	0.651351
Thermal correction to Gibbs Free Energy=	0.540263
Sum of electronic and zero-point Energies=	-2243.427608
Sum of electronic and thermal Energies=	-2243.389278
Sum of electronic and thermal Enthalpies=	-2243.388334
Sum of electronic and thermal Free Energies=	-2243.499422

C	3.196414	-0.818194	-0.808836	C	3.871844	3.979414	-0.134632
C	2.710280	0.477850	-0.526885	C	2.336635	-1.889050	-1.467089
C	3.524755	1.470417	0.060406	C	2.406272	-3.241245	-0.743382
C	4.852098	1.141359	0.361421	C	0.884471	1.183091	-2.162519
C	5.352873	-0.129828	0.085230	C	-0.540200	1.723078	-1.977059
C	4.533258	-1.096518	-0.494068	N	-1.112624	0.936768	-0.878903
N	1.344325	0.815689	-0.813373	C	-2.515035	0.846767	-0.597552
P	0.060731	0.345842	0.179064	C	-3.149871	1.887554	0.111661
C	-0.174428	-1.193213	0.994287	C	-4.523729	1.773992	0.359309
O	-0.373450	-2.421636	0.795757	C	-5.233978	0.651467	-0.062128
C	2.979682	2.849934	0.399304	C	-4.581591	-0.383390	-0.731062

C	-3.210958	-0.312526	-1.010136
C	-2.382189	3.102605	0.611311
C	-2.811229	4.376221	-0.134434
C	-2.518859	-1.437119	-1.768314
C	-2.748388	-1.270533	-3.280964
P	0.024425	0.014773	2.279139
C	-2.937232	-2.835191	-1.294776
C	-2.514553	3.271086	2.132320
C	2.757686	2.986274	1.914758
C	2.722029	-2.038937	-2.949826
H	0.885863	0.308808	-2.836545
H	1.556585	1.947589	-2.578038
H	-1.142764	1.577894	-2.884513
H	-0.526622	2.801987	-1.736764
H	5.500670	1.887314	0.823733
H	6.389822	-0.371095	0.328374
H	4.934731	-2.089500	-0.699781
H	-5.145422	-1.265012	-1.037029
H	-6.303666	0.575511	0.144071
H	-5.041776	2.568260	0.899661
H	1.288155	-1.558606	-1.430183
H	1.998055	2.941729	-0.087557
H	-1.438938	-1.344625	-1.589397
H	2.316675	3.966636	2.158565
H	2.084446	2.201045	2.291877
H	3.710361	2.896241	2.462022
H	3.402581	4.958422	0.053480
H	4.857888	3.988890	0.357121
H	4.038862	3.880091	-1.218716
H	1.704002	-3.949678	-1.208777
H	3.413908	-3.682546	-0.810428
H	2.129133	-3.163557	0.315616
H	2.062614	-2.766719	-3.449328
H	2.652062	-1.080237	-3.488032
H	3.759541	-2.397804	-3.048542
H	-2.208953	-2.049618	-3.843685
H	-3.820172	-1.354470	-3.526268
H	-2.403333	-0.286941	-3.639065
H	-2.344172	-3.596372	-1.825688
H	-2.761056	-2.974865	-0.219544
H	-3.998304	-3.040491	-1.511176
H	-1.316089	2.935748	0.398055
H	-1.891552	4.110232	2.481645
H	-3.554634	3.482847	2.427659
H	-2.194465	2.359585	2.659959
H	-2.215285	5.242104	0.196819
H	-2.683705	4.264670	-1.222934
H	-3.872661	4.606391	0.053339
B	-0.519545	-3.424328	2.051463
F	-1.711024	-4.072586	1.822914
F	0.598435	-4.225332	1.979370
F	-0.537702	-2.609164	3.183432

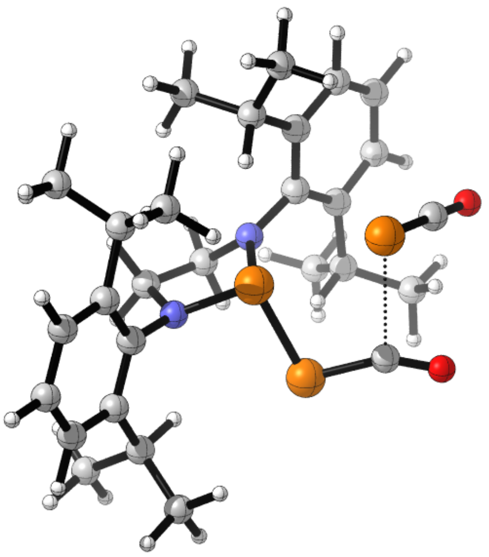

Imiginary frequency	-75.6 (Int. 38)
Zero-point correction=	0.604889 (Hartree/Particle)
Thermal correction to Energy=	0.643536
Thermal correction to Enthalpy=	0.644481
Thermal correction to Gibbs Free Energy=	0.534144
Sum of electronic and zero-point Energies=	-2373.630527
Sum of electronic and thermal Energies=	-2373.591880
Sum of electronic and thermal Enthalpies=	-2373.590936
Sum of electronic and thermal Free Energies=	-2373.701272

C	-3.350448	1.447065	0.185786
C	-3.001032	0.126465	-0.190497
C	-3.969193	-0.906371	-0.142579
C	-5.265047	-0.606469	0.296145
C	-5.607390	0.685275	0.692317
C	-4.653426	1.699845	0.635933
N	-1.684501	-0.195442	-0.624107
P	-0.290080	-0.078667	0.512312
P	-0.242262	-2.303694	0.984266
C	1.044915	-2.351643	2.136200
O	1.781802	-2.948206	2.828783
C	-3.621213	-2.323112	-0.569995
C	-4.377746	-2.717086	-1.848346
C	-2.351647	2.592600	0.106259
C	-1.944780	3.087648	1.502237
C	-1.342374	-0.006208	-2.026240
C	0.125869	-0.400531	-2.117647
N	0.739705	0.134929	-0.903714
C	2.140690	0.421006	-0.886436
C	3.099162	-0.567011	-1.203283
C	4.442647	-0.185135	-1.331151
C	4.847158	1.121860	-1.093038
C	3.903700	2.073846	-0.704589
C	2.550335	1.746735	-0.596942
C	2.735107	-2.036182	-1.365811
C	2.848515	-2.489000	-2.830731
C	1.527042	2.803649	-0.211708
C	0.933245	3.450357	-1.472584
C	2.072905	3.859693	0.753431
C	3.579015	-2.940761	-0.453584

C	-3.861921	-3.337598	0.555859
C	-2.881743	3.744219	-0.761507
H	-1.486919	1.047619	-2.346841
H	-1.965258	-0.643760	-2.681359
H	0.618942	0.030981	-3.001872
H	0.199104	-1.502773	-2.180047
H	-6.014507	-1.400556	0.336794
H	-6.619283	0.902480	1.044126
H	-4.926181	2.712160	0.943440
H	4.229752	3.090289	-0.480461
H	5.901741	1.396084	-1.176019
H	5.188094	-0.942931	-1.580063
H	-1.447069	2.207849	-0.380128
H	-2.545684	-2.326320	-0.793467
H	0.719305	2.288083	0.324232
H	-3.539097	-4.343664	0.240365
H	-3.282503	-3.065417	1.450278
H	-4.929364	-3.396609	0.830606
H	-4.068541	-3.719079	-2.190938
H	-5.468336	-2.741553	-1.679125
H	-4.184290	-2.001111	-2.663378
H	-1.199624	3.896102	1.422959
H	-2.815638	3.478950	2.055502
H	-1.494851	2.272583	2.087401
H	-2.114724	4.528236	-0.868522
H	-3.153514	3.390772	-1.769105
H	-3.775651	4.213505	-0.317323
H	0.164569	4.196487	-1.210406
H	1.719493	3.959911	-2.055560
H	0.467689	2.692180	-2.120758
H	1.253397	4.516821	1.089520
H	2.510311	3.374171	1.638591
H	2.834768	4.505466	0.284073
H	1.696717	-2.159694	-1.035070
H	3.187857	-3.971483	-0.486311
H	4.634215	-2.972256	-0.774689
H	3.558640	-2.590011	0.585999
H	2.546749	-3.544958	-2.938333
H	2.214467	-1.882820	-3.496844
H	3.888227	-2.398273	-3.189876
P	1.759383	0.333813	2.759492
C	3.230905	-0.218682	2.270250
O	4.291379	-0.625856	1.917444

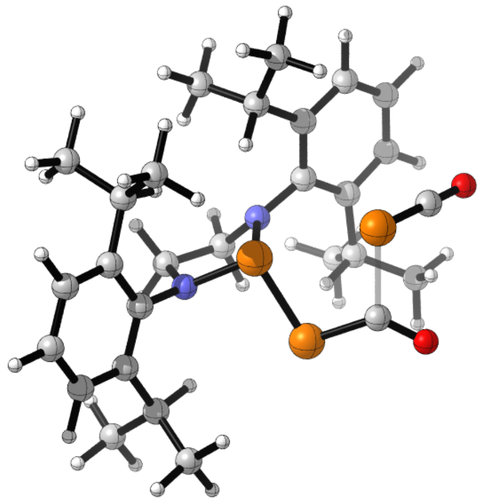

Zero-point correction=	0.604903 (Hartree/Particle)
Thermal correction to Energy=	0.644138
Thermal correction to Enthalpy=	0.645083

Thermal correction to Gibbs Free Energy=	0.532808
Sum of electronic and zero-point Energies=	-2373.632270
Sum of electronic and thermal Energies=	-2373.593035
Sum of electronic and thermal Enthalpies=	-2373.592090
Sum of electronic and thermal Free Energies=	-2373.704365

C	3.389709	1.414793	-0.220402	H	1.560354	1.142646	2.314599
C	3.017002	0.107472	0.178420	H	2.005798	-0.544981	2.709498
C	3.960103	-0.947783	0.126797	H	-0.545536	0.203124	3.067361
C	5.255293	-0.681391	-0.336123	H	-0.191302	-1.366134	2.280377
C	5.621291	0.598006	-0.750288	H	5.985252	-1.493271	-0.380737
C	4.691092	1.634607	-0.691308	H	6.632612	0.788424	-1.119119
N	1.702930	-0.180229	0.642827	H	4.981453	2.637356	-1.014263
P	0.294438	-0.083926	-0.466825	H	-4.181591	3.160255	0.219385
P	0.126556	-2.266665	-0.878801	H	-5.879363	1.523607	0.989961
C	-1.191400	-2.236373	-2.091708	H	-5.181303	-0.773327	1.602410
O	-1.792911	-3.116566	-2.637468	H	1.498727	2.214783	0.333886
C	3.587054	-2.352845	0.570951	H	2.519369	-2.327077	0.826988
C	4.374194	-2.764508	1.824873	H	-0.651317	2.302512	-0.409255
C	2.414392	2.580638	-0.145991	H	3.433206	-4.372543	-0.228940
C	2.025929	3.080448	-1.545238	H	3.143961	-3.092211	-1.427137
C	1.385257	0.080169	2.040475	H	4.814505	-3.452183	-0.882907
C	-0.092582	-0.268774	2.181161	H	4.046942	-3.756046	2.180873
N	-0.708218	0.258787	0.967608	H	5.457892	-2.822047	1.621621
C	-2.105798	0.532521	0.907614	H	4.227838	-2.041108	2.643473
C	-3.080278	-0.429612	1.263451	H	1.298521	3.905788	-1.472682
C	-4.426381	-0.037652	1.317773	H	2.906851	3.450736	-2.096919
C	-4.822904	1.245603	0.963310	H	1.562111	2.270659	-2.127004
C	-3.865218	2.163887	0.530995	H	2.214905	4.522808	0.826259
C	-2.509106	1.830756	0.498750	H	3.223629	3.362616	1.731874
C	-2.729412	-1.884498	1.545620	H	3.871794	4.169890	0.283178
C	-2.863839	-2.218375	3.040178	H	-0.116811	4.299654	1.006853
C	-1.470059	2.855994	0.069509	H	-1.692253	4.130012	1.831204
C	-0.898415	3.578215	1.298998	H	-0.453934	2.856510	2.000785
C	-1.984732	3.854405	-0.971772	H	-1.151141	4.477252	-1.336293
C	-3.564940	-2.855555	0.697194	H	-2.420289	3.326800	-1.833971
C	3.761169	-3.373630	-0.561361	H	-2.745517	4.539809	-0.560503
C	2.964794	3.721664	0.722895	H	-1.690256	-2.040986	1.232720

H	-3.198383	-3.885063	0.843646
H	-4.632212	-2.835253	0.975670
H	-3.487988	-2.620573	-0.371983
H	-2.565753	-3.262441	3.236345
H	-2.236631	-1.561054	3.662621
H	-3.907941	-2.097464	3.377652
P	-1.783167	-0.185466	-2.796495
C	-3.343676	-0.460612	-2.299361
O	-4.454317	-0.640426	-1.946435

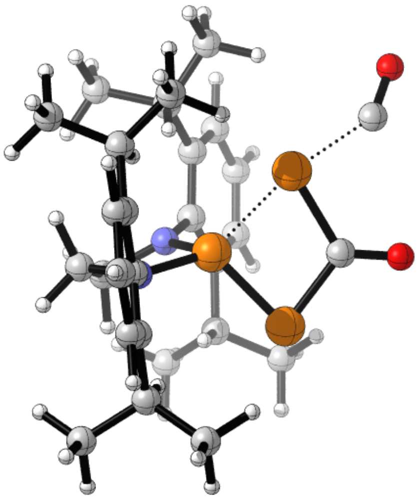

Imaginary frequency	-266 (Int. 4)
Zero-point correction=	0.603328 (Hartree/Particle)
Thermal correction to Energy=	0.641399
Thermal correction to Enthalpy=	0.642344
Thermal correction to Gibbs Free Energy=	0.531722
Sum of electronic and zero-point Energies=	-2373.607042
Sum of electronic and thermal Energies=	-2373.568970
Sum of electronic and thermal Enthalpies=	-2373.568026
Sum of electronic and thermal Free Energies=	-2373.678647

C	3.066302	-1.436292	-0.544977
C	2.761886	-0.055925	-0.480027
C	3.748535	0.883178	-0.099646
C	5.039249	0.423818	0.193073
C	5.352840	-0.932140	0.118195
C	4.370956	-1.851682	-0.246792
N	1.457528	0.419740	-0.803935
P	0.121780	0.277538	0.349939
P	0.233947	1.710798	1.930026
C	-0.395555	0.257398	2.950300
O	-0.687156	0.336883	4.130386
C	3.436314	2.370091	-0.042860
C	4.102835	3.109408	-1.214969
C	2.021071	-2.469988	-0.941009
C	1.867881	-3.573664	0.114284
C	1.047390	0.496513	-2.199636
C	-0.352832	1.108293	-2.164960
N	-0.980107	0.485207	-1.009259
C	-2.393435	0.402614	-0.874616
C	-3.170109	1.554808	-0.626210

C	-4.566023	1.427439	-0.571988
C	-5.179808	0.187793	-0.729731
C	-4.401324	-0.951340	-0.940246
C	-3.006901	-0.867279	-1.013568
C	-2.527965	2.920003	-0.432811
C	-2.799757	3.829415	-1.642289
C	-2.170123	-2.107915	-1.285014
C	-2.071402	-2.366869	-2.797053
C	-2.659769	-3.349242	-0.533152
C	-2.969078	3.581705	0.878661
C	3.816548	2.996876	1.303989
C	2.333215	-3.067348	-2.323335
H	1.011693	-0.497101	-2.690152
H	1.751261	1.131503	-2.766269
H	-0.921955	0.875291	-3.079064
H	-0.282134	2.213069	-2.078316
H	5.809558	1.140366	0.486574
H	6.364347	-1.274614	0.351968
H	4.617856	-2.914631	-0.293413
H	-4.887523	-1.922680	-1.047165
H	-6.268544	0.104195	-0.677312
H	-5.177807	2.313241	-0.386404
H	1.053640	-1.954626	-1.006240
H	2.348831	2.465903	-0.154621
H	-1.158731	-1.890914	-0.924393
H	3.522862	4.059796	1.321700
H	3.284235	2.487994	2.120769
H	4.903113	2.947100	1.492582
H	3.833581	4.179437	-1.204157
H	5.203320	3.038220	-1.160576
H	3.790016	2.687316	-2.183982
H	1.048029	-4.254947	-0.168613
H	2.788169	-4.175056	0.209758
H	1.613236	-3.136734	1.090818
H	1.536456	-3.764870	-2.632344
H	2.421333	-2.283590	-3.092486
H	3.284381	-3.626783	-2.307354
H	-1.418044	-3.232212	-3.003727
H	-3.065107	-2.576317	-3.229926
H	-1.653774	-1.492915	-3.321770
H	-1.944390	-4.176595	-0.672609
H	-2.729510	-3.142100	0.544561
H	-3.641140	-3.699449	-0.897340
H	-1.444998	2.763991	-0.347642
H	-2.430669	4.533334	1.021092
H	-4.050821	3.799754	0.888030
H	-2.721497	2.930331	1.728903
H	-2.287758	4.799696	-1.525415
H	-2.447026	3.366235	-2.578053
H	-3.879340	4.029119	-1.756885
P	-0.540392	-1.371780	1.894835
C	-1.599413	-2.321439	3.325364
O	-2.382382	-3.164642	3.485360

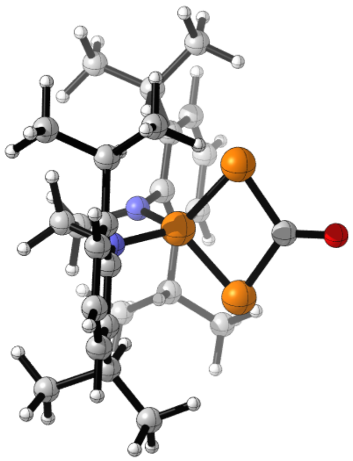

Zero-point correction=	0.597573 (Hartree/Particle)
Thermal correction to Energy=	0.632873
Thermal correction to Enthalpy=	0.633817
Thermal correction to Gibbs Free Energy=	0.532029
Sum of electronic and zero-point Energies=	-2260.298135
Sum of electronic and thermal Energies=	-2260.262836
Sum of electronic and thermal Enthalpies=	-2260.261892
Sum of electronic and thermal Free Energies=	-2260.363680

C	3.321173	1.212630	-0.393486
C	2.616930	-0.013873	-0.459490
C	3.296862	-1.243581	-0.307069
C	4.686102	-1.226052	-0.116780
C	5.388929	-0.024992	-0.061624
C	4.707120	1.184022	-0.195478
N	1.217780	0.019331	-0.717623
C	0.703982	-0.293453	-2.042201
C	-0.704015	0.293574	-2.042193
N	-1.217786	-0.019323	-0.717613
P	-0.000008	0.000149	0.555278
P	0.207684	1.564761	1.999917
C	-0.000005	0.000479	3.089730
O	0.000064	0.000591	4.306064
C	2.566064	-2.575857	-0.384069
C	2.850699	-3.464174	0.833184
C	2.605945	2.540013	-0.590178
C	3.012188	3.593100	0.445933
C	-2.616951	0.013765	-0.459483
C	-3.297051	1.243391	-0.307241
C	-4.686306	1.225688	-0.117064
C	-5.388975	0.024543	-0.061821
C	-4.706991	-1.184397	-0.195452
C	-3.321030	-1.212837	-0.393362
C	-2.566403	2.575740	-0.384256
C	-2.898020	3.304616	-1.697521
C	-2.605616	-2.540152	-0.589869
C	-3.011697	-3.593147	0.446394
P	-0.207666	-1.564145	2.000246
C	-2.812447	-3.053575	-2.024774
C	-2.851059	3.463937	0.833075
C	2.897575	-3.304711	-1.697376
C	2.812867	3.053208	-2.025156
H	-0.658627	1.384426	-2.241923
H	-1.340412	-0.169272	-2.813907
H	1.340387	0.169383	-2.813917

H	0.658516	-1.384294	-2.242002	H	-2.535180	2.949068	1.751806
H	-5.260901	-2.123933	-0.145966	H	-2.315824	4.237919	-1.781839
H	-6.471447	0.029154	0.091660	H	-2.670481	2.677926	-2.574769
H	-5.222767	2.170220	-0.004024	H	-3.968549	3.569177	-1.746296
H	5.261151	2.123489	-0.146056	H	2.257266	3.992581	-2.188666
H	6.471384	-0.029726	0.091974	H	3.880199	3.249771	-2.228350
H	5.222423	-2.170643	-0.003579	H	2.462971	2.317103	-2.766705
H	-1.489988	2.360593	-0.368383	H	2.403121	4.503153	0.316207
H	-1.534477	-2.347046	-0.452718	H	2.832054	3.208291	1.460306
H	1.534781	2.347056	-0.452992	H	4.071707	3.887384	0.350064
H	-2.402535	-4.503148	0.316741	H	1.489670	-2.360600	-0.368135
H	-2.831550	-3.208210	1.460718	H	2.274193	-4.401473	0.759987
H	-4.071190	-3.887564	0.350630	H	3.918841	-3.731390	0.907754
H	-2.256690	-3.992877	-2.188156	H	2.535041	-2.949338	1.752003
H	-3.879745	-3.250346	-2.227952	H	2.315338	-4.237991	-1.781699
H	-2.462664	-2.317510	-2.766417	H	2.670043	-2.677994	-2.574604
H	-2.274741	4.401354	0.759883	H	3.968090	-3.569313	-1.746183
H	-3.919244	3.730930	0.907802				

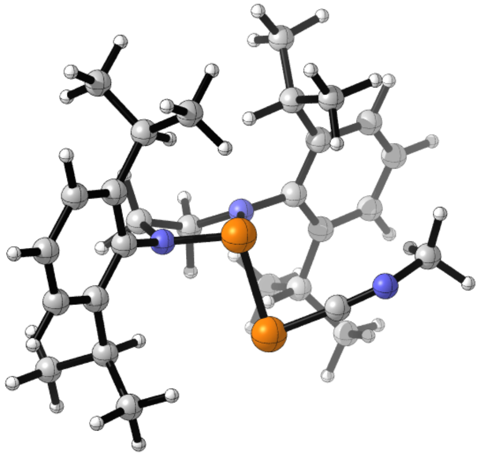

Zero-point correction=	0.635917 (Hartree/Particle)
Thermal correction to Energy=	0.672504
Thermal correction to Enthalpy=	0.673448
Thermal correction to Gibbs Free Energy=	0.568569
Sum of electronic and zero-point Energies=	-1938.250564
Sum of electronic and thermal Energies=	-1938.213977
Sum of electronic and thermal Enthalpies=	-1938.213033

Sum of electronic and thermal Free Energies= -1938.317911

C	-3.279966	1.412890	-0.007236	H	-6.533255	0.767804	0.827218
C	-2.845154	0.079242	-0.187727	H	-4.964127	2.659034	0.512381
C	-3.733966	-1.005935	-0.014212	H	4.470537	2.849486	0.037772
C	-5.059607	-0.735435	0.348742	H	6.139629	1.045017	-0.301217
C	-5.497483	0.573743	0.539757	H	5.376333	-1.255963	-0.815379
C	-4.612623	1.636085	0.362811	H	-1.379070	2.211568	-0.548175
N	-1.494023	-0.200849	-0.567161	H	-2.202443	-2.407504	-0.435363
P	-0.167898	0.023244	0.536422	H	0.791027	2.385237	-0.435566
P	0.088471	-2.130227	1.428343	H	-3.091689	-4.317806	0.861597
C	1.256313	-1.432914	2.417449	H	-2.971684	-2.870190	1.891094
C	-3.281424	-2.441410	-0.230140	H	-4.559857	-3.399578	1.274430
C	-3.974667	-3.060286	-1.454513	H	-3.592688	-4.076687	-1.644680
C	-2.345756	2.599314	-0.199365	H	-5.064563	-3.133999	-1.302999
C	-2.091468	3.329229	1.127991	H	-3.805923	-2.454105	-2.358905
C	-1.121705	-0.153616	-1.983325	H	-1.394760	4.170106	0.978918
C	0.348908	-0.554096	-2.023775	H	-3.027095	3.736550	1.544845
N	0.929016	0.049858	-0.820300	H	-1.652326	2.647760	1.871813
C	2.328944	0.278230	-0.656014	H	-2.135180	4.370812	-1.453409
C	3.276110	-0.769322	-0.792713	H	-3.034925	3.039348	-2.231485
C	4.640262	-0.460584	-0.687277	H	-3.815702	4.032181	-0.978714
C	5.072924	0.829668	-0.393204	H	1.336365	4.724803	-0.931674
C	4.133167	1.840309	-0.205391	H	3.077629	4.409825	-0.776446
C	2.761589	1.594328	-0.345582	H	2.157820	3.654775	-2.100295
C	2.873949	-2.217484	-1.042370	H	0.990232	3.954278	1.493546
C	3.132927	-2.624377	-2.502414	H	1.431283	2.294420	1.964886
C	1.785704	2.744943	-0.144006	H	2.706487	3.514226	1.687539
C	2.111813	3.948840	-1.039967	H	1.801558	-2.303282	-0.832797
C	1.726103	3.149501	1.337850	H	3.175426	-4.209730	-0.228916
C	3.567875	-3.191337	-0.078666	H	4.657484	-3.229619	-0.237066
C	-3.490697	-3.303287	1.022859	H	3.379428	-2.912298	0.968275
C	-2.864237	3.562982	-1.277666	H	2.786947	-3.654402	-2.687831
H	-1.268015	0.863422	-2.397340	H	2.617621	-1.956827	-3.209448
H	-1.741865	-0.852553	-2.568583	H	4.210051	-2.583258	-2.734968
H	0.858306	-0.165590	-2.917691	N	2.023120	-0.845976	3.151396
H	0.428351	-1.655816	-2.028690	C	3.401347	-0.430014	2.963654
H	-5.758104	-1.563135	0.486992	H	3.485825	0.634660	3.226641

H	4.046129	-0.999316	3.651446	H	3.738328	-0.575399	1.925916

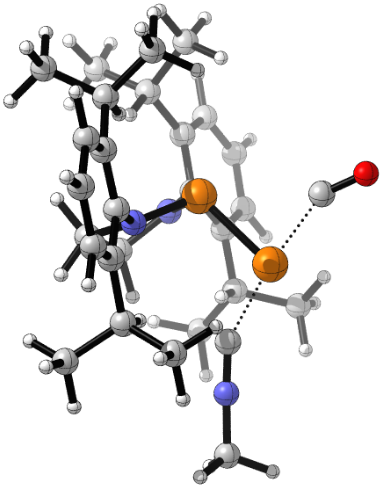

Imaginary frequency	-412 (Int.4)
Zero-point correction=	0.639352 (Hartree/Particle)
Thermal correction to Energy=	0.680264
Thermal correction to Enthalpy=	0.681208
Thermal correction to Gibbs Free Energy=	0.563638
Sum of electronic and zero-point Energies=	-2051.526520
Sum of electronic and thermal Energies=	-2051.485608
Sum of electronic and thermal Enthalpies=	2051.484664
Sum of electronic and thermal Free Energies=	-2051.602235

C	2.923062	-1.942474	-0.038967	C	1.840946	-3.707446	1.428488
C	2.580590	-0.594978	-0.332014	C	0.698207	-0.232139	-1.932794
C	3.603654	0.385863	-0.437876	C	-0.730131	0.308605	-1.850809
C	4.937170	0.007260	-0.231389	N	-1.251349	-0.180310	-0.571898
C	5.274752	-1.306916	0.082006	C	-2.649781	-0.437695	-0.373690
C	4.271297	-2.267083	0.175198	C	-3.579332	0.631660	-0.277750
N	1.219798	-0.195727	-0.563229	C	-4.941475	0.337862	-0.121761
P	-0.032292	-0.283599	0.687335	C	-5.394184	-0.976163	-0.037903
P	0.111952	1.696562	1.825504	C	-4.479360	-2.021532	-0.129372
C	0.054206	0.561751	3.512118	C	-3.109491	-1.781478	-0.311479
O	0.058442	0.451178	4.655031	C	-3.148739	2.091536	-0.354810
C	3.295351	1.837711	-0.783787	C	-3.595054	2.738498	-1.679709
C	3.855987	2.221944	-2.166404	C	-2.174703	-2.978540	-0.450423
C	1.893001	-3.065978	0.030368	C	-2.581162	-3.893538	-1.620358

C	-2.077005	-3.772609	0.864507
C	-3.640377	2.910490	0.850464
C	3.796261	2.807669	0.300419
C	2.139997	-4.128333	-1.057694
H	0.699492	-1.264161	-2.340224
H	1.318084	0.388963	-2.603259
H	-1.350167	-0.070320	-2.678073
H	-0.722637	1.415907	-1.906810
H	5.726264	0.758600	-0.311487
H	6.318701	-1.583522	0.248343
H	4.540658	-3.299512	0.409370
H	-4.837291	-3.051912	-0.070930
H	-6.458387	-1.185179	0.095184
H	-5.660745	1.157112	-0.054699
H	0.904469	-2.628003	-0.160694
H	2.201472	1.924171	-0.821959
H	-1.175953	-2.583643	-0.679056
H	3.507096	3.843932	0.053134
H	3.364063	2.558889	1.281479
H	4.895154	2.789009	0.392450
H	3.564557	3.252533	-2.432926
H	4.958109	2.175545	-2.183449
H	3.485816	1.549464	-2.957137
H	1.064887	-4.489073	1.467364
H	2.801671	-4.178478	1.695445
H	1.605458	-2.956744	2.198307
H	1.346127	-4.893584	-1.035686
H	2.153165	-3.678939	-2.063678
H	3.103069	-4.645343	-0.911660
H	-1.835189	-4.694077	-1.758144
H	-3.555633	-4.378751	-1.444692
H	-2.654779	-3.330727	-2.564760
H	-1.387520	-4.625909	0.752672
H	-1.705571	-3.137902	1.683610
H	-3.059166	-4.175361	1.164385
H	-2.052606	2.105637	-0.320545
H	-3.226653	3.932588	0.810929
H	-4.738993	3.003273	0.871010
H	-3.312815	2.449419	1.793385
H	-3.230577	3.777876	-1.750030
H	-3.212986	2.183761	-2.551486
H	-4.694831	2.766422	-1.761437
C	0.182532	3.065876	0.281620
N	0.244162	4.166644	-0.135768
C	0.294151	5.512606	-0.583863
H	0.095082	6.193315	0.258598
H	1.290046	5.733743	-0.998164
H	-0.463871	5.678309	-1.365267

Methylisonitrile

Zero-point correction=	0.044758 (Hartree/Particle)
Thermal correction to Energy=	0.048617
Thermal correction to Enthalpy=	0.049562
Thermal correction to Gibbs Free Energy=	0.020531
Sum of electronic and zero-point Energies=	-132.683859
Sum of electronic and thermal Energies=	-132.679999
Sum of electronic and thermal Enthalpies=	-132.679055
Sum of electronic and thermal Free Energies=	-132.708085

N	0.004053	0.000000	0.038991
C	-0.004123	0.000000	1.217382
C	0.014375	0.000000	-1.382110
H	-1.017344	0.000000	-1.765387

H	0.533764	0.895914	-1.754308	H	0.533764	-0.895914	-1.754308

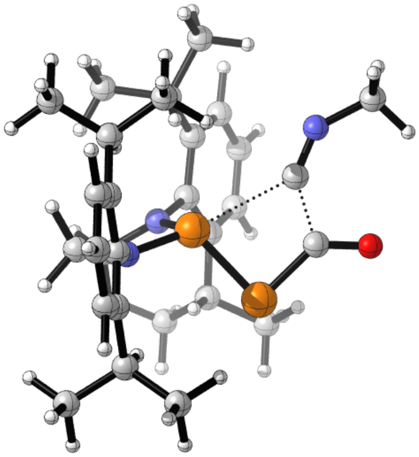

Imaginary frequency	-81 (Int. 51)
Zero-point correction=	0.643844 (Hartree/Particle)
Thermal correction to Energy=	0.682718
Thermal correction to Enthalpy=	0.683663
Thermal correction to Gibbs Free Energy=	0.571767
Sum of electronic and zero-point Energies=	2051.536186
Sum of electronic and thermal Energies=	-2051.497312
Sum of electronic and thermal Enthalpies=	-2051.496367
Sum of electronic and thermal Free Energies=	-2051.608263

C	2.899320	1.088039	-0.920240	C	3.220280	-3.467860	0.653214
C	2.390507	-0.235944	-0.906100	C	1.959935	2.276717	-1.080340
C	3.245054	-1.343583	-0.725680	C	2.493246	3.574702	-0.465819
C	4.626547	-1.113863	-0.642147	C	-2.763193	0.037419	-0.438230
C	5.142864	0.177366	-0.685996	C	-3.046927	1.422618	-0.465001
C	4.281500	1.269652	-0.805885	C	-4.337668	1.842869	-0.119312
N	0.981036	-0.415571	-1.054323	C	-5.322281	0.921807	0.236108
C	0.354778	-1.082839	-2.195388	C	-5.027920	-0.440033	0.256632
C	-1.084462	-0.563956	-2.197001	C	-3.748493	-0.905863	-0.073950
N	-1.459291	-0.446238	-0.784132	C	-1.994553	2.457200	-0.841860
P	-0.104996	-0.192107	0.267994	C	-2.431679	3.301696	-2.048087
C	0.869671	0.966141	2.430295	C	-3.444839	-2.395230	-0.050923
N	1.254863	2.029942	2.807571	C	-3.748569	-3.020911	1.317326
C	1.759502	2.662981	4.002462	P	-0.069315	-1.721845	1.830980
C	2.706499	-2.761570	-0.609537	C	0.668472	-0.537133	2.962365

O	1.100889	-0.608331	4.103367	H	-3.948234	-2.695216	-2.164413
C	-4.192081	-3.124325	-1.179190	H	-0.829753	4.054255	0.080190
C	-1.625130	3.343197	0.357639	H	-2.493358	3.925233	0.707387
C	3.021195	-3.579128	-1.872587	H	-1.257931	2.734180	1.196577
C	1.592846	2.484047	-2.558607	H	-1.626822	3.994075	-2.343878
H	-1.149421	0.411095	-2.716966	H	-2.672809	2.664785	-2.913703
H	-1.764343	-1.267439	-2.704036	H	-3.323755	3.906901	-1.818738
H	0.885264	-0.818495	-3.122161	H	0.862722	3.302753	-2.669255
H	0.361062	-2.184421	-2.094853	H	2.487044	2.739767	-3.150836
H	-5.802932	-1.155114	0.539267	H	1.150466	1.574748	-2.990268
H	-6.323637	1.268612	0.500923	H	1.713394	4.351802	-0.504315
H	-4.576128	2.908433	-0.127380	H	2.775209	3.429082	0.588100
H	4.696136	2.277721	-0.818235	H	3.368644	3.966849	-1.008806
H	6.220674	0.338470	-0.612271	H	1.615895	-2.690874	-0.504184
H	5.303639	-1.961425	-0.521170	H	2.750338	-4.459792	0.749321
H	-1.083346	1.921735	-1.136904	H	4.311830	-3.617580	0.625521
H	-2.366522	-2.500975	-0.233650	H	2.970234	-2.887317	1.553415
H	1.035381	2.031994	-0.538950	H	2.587326	-4.589994	-1.801875
H	-3.454934	-4.083079	1.325025	H	2.620422	-3.095291	-2.777275
H	-3.189091	-2.508308	2.113995	H	4.109925	-3.688203	-2.010269
H	-4.823213	-2.970471	1.559281	H	2.731106	3.126954	3.779749
H	-3.925323	-4.193802	-1.194435	H	1.062210	3.455972	4.309807
H	-5.284705	-3.051958	-1.047071	H	1.862101	1.908071	4.797025

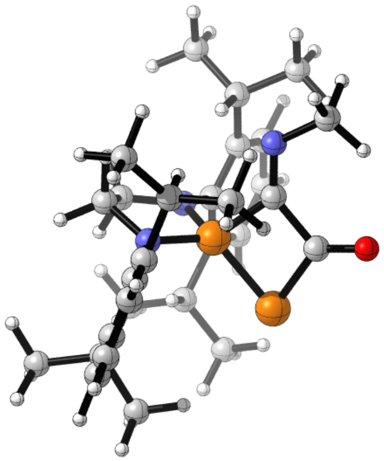

Zero-point correction=	0.647139 (Hartree/Particle)
Thermal correction to Energy=	0.684738
Thermal correction to Enthalpy=	0.685682

Thermal correction to Gibbs Free Energy= 0.577967
Sum of electronic and zero-point Energies= -2051.585825
Sum of electronic and thermal Energies= -2051.548226
Sum of electronic and thermal Enthalpies= -2051.547281
Sum of electronic and thermal Free Energies= -2051.654996

C	-3.523188	-0.637271	-0.587528	C	3.245340	-3.093383	0.505820
C	-2.585523	0.401323	-0.397234	C	-2.197118	3.904493	-0.697962
C	-2.969858	1.652976	0.137534	C	-3.639878	-2.107521	-2.624580
C	-4.317519	1.846835	0.465434	H	0.741087	-0.826773	-2.662923
C	-5.256185	0.833746	0.278253	H	1.343214	0.853127	-2.772380
C	-4.859498	-0.396441	-0.241115	H	-1.350036	0.316553	-2.883272
N	-1.223008	0.172213	-0.782581	H	-0.739222	1.791353	-2.082004
C	-0.719415	0.687187	-2.061417	H	4.752541	2.853262	0.261552
C	0.717783	0.168430	-2.182576	H	6.341359	0.952455	0.324068
N	1.227138	0.099788	-0.809724	H	5.548726	-1.351775	-0.123982
P	0.011665	-0.218115	0.327803	H	-5.599944	-1.185825	-0.381190
C	-0.106129	-1.924373	1.005032	H	-6.302197	1.003812	0.542489
N	-0.239174	-3.014743	0.371828	H	-4.635934	2.805639	0.878089
C	-0.282793	-4.278318	1.103548	H	1.949910	-2.170085	-0.919259
C	-1.978981	2.790834	0.340458	H	1.077973	2.421999	-0.459548
C	-2.023616	3.357368	1.766575	H	-2.026046	-2.015874	-1.211444
C	-3.121837	-1.977782	-1.182890	H	1.293230	4.385841	1.013031
C	-3.591529	-3.156098	-0.318984	H	1.765586	2.900070	1.881494
C	2.604884	0.317747	-0.492385	H	3.013532	4.032600	1.298199
C	3.495212	-0.774811	-0.454844	H	1.684752	4.582176	-1.506454
C	4.841376	-0.521114	-0.158783	H	3.417783	4.215017	-1.320193
C	5.288707	0.774014	0.093982	H	2.417465	3.227127	-2.408135
C	4.393725	1.842692	0.057266	H	2.856287	-4.106082	0.314937
C	3.039847	1.637642	-0.233703	H	4.315325	-3.186611	0.753838
C	3.029625	-2.197407	-0.723262	H	2.729438	-2.690408	1.390367
C	3.711487	-2.782936	-1.969510	H	3.322029	-3.791578	-2.182808
C	2.084831	2.820804	-0.278618	H	3.532927	-2.151696	-2.854639
C	2.039688	3.575959	1.057482	H	4.802360	-2.866588	-1.833310
P	0.193058	0.367012	2.364536	H	-3.307038	-3.058265	-3.072143
C	0.052259	-1.507628	2.474322	H	-4.742095	-2.084704	-2.657331
O	0.092280	-2.244236	3.440618	H	-3.273170	-1.283981	-3.258154
C	2.420099	3.763140	-1.445447	H	-3.199794	-4.102281	-0.725058

H	-3.234185	-3.053619	0.716670	H	-1.424657	4.684631	-0.597275
H	-4.690660	-3.236768	-0.292386	H	-2.160732	3.513913	-1.726906
H	-0.968085	2.388587	0.187041	H	-3.180644	4.383212	-0.560681
H	-1.224305	4.103249	1.903046	H	-1.245235	-4.767507	0.879858
H	-2.983290	3.857253	1.975035	H	0.503509	-4.934597	0.695474
H	-1.877481	2.561186	2.510438	H	-0.158842	-4.169067	2.189978

5. References

1 (a) L. Liu; D. A. Ruiz, D. Munz and G. Bertrand, *Chem*, 2016, ***1***, 147-153; (b) M. M. Hansmann, R. Jazzar and G. Bertrand, *J. Am. Chem. Soc.*, 2016, ***138***, 8356-8359

2 M. Ibarra-Rodriguez, H. V. R. Dias, V. M. Jimenez-Perez, B. M. Munoz-Flores, A. Flores-Parra, S. Sanchez, *Z. Anorg. Allgem. Chem.*, 2012, ***638***, 1486-1490.

3 Gaussian 09, Revision D.01, Frisch, M. J.; Trucks, G. W.; Schlegel, H. B.; Scuseria, G. E.; Robb, M. A.; Cheeseman, J. R.; Scalmani, G.; Barone, V.; Mennucci, B.; Petersson, G. A.; Nakatsuji, H.; Caricato, M.; Li, X.; Hratchian, H. P.; Izmaylov, A. F.; Bloino, J.; Zheng, G.; Sonnenberg, J. L.; Hada, M.; Ehara, M.; Toyota, K.; Fukuda, R.; Hasegawa, J.; Ishida, M.; Nakajima, T.; Honda, Y.; Kitao, O.; Nakai, H.; Vreven, T.; Montgomery, J. A. Jr.; Peralta, J. E.; Ogliaro, F.; Bearpark, M.; Heyd, J. J.; Brothers, E.; Kudin, K. N.; Staroverov, V. N.; Keith, T.; Kobayashi, R.; Normand, J.; Raghavachari, K.; Rendell, A.; Burant, J. C.; Iyengar, S. S.; Tomasi, J.; Cossi, M.; Rega, N.; Millam, J. M.; Klene, M.; Knox, J. E.; Cross, J. B.; Bakken, V.; Adamo, C.; Jaramillo, J.; Gomperts, R.; Stratmann, R. E.; Yazyev, O.;. Austin, A. J; Cammi, R.; Pomelli, C.; Ochterski, J. W.; Martin, R. L.; Morokuma, K.; Zakrzewski, V. G.; Voth, G. A.; Salvador, P.; Dannenberg, J. J.; Dapprich, S.; Daniels, A. D.; Farkas, O.; Foresman, J. B.; Ortiz, J. V.; Cioslowski, J.; Fox, D. J. Gaussian, Inc., Wallingford CT, **2013**.

4 (a) C. Lee, W. Yang, R. G. Parr, Phys. Rev. B 1988, 37, 785−789. (b) P. J. Stephens, F. J. Devlin, C. F. Chabalowski, M. J. Frisch, *J. Phys. Chem.* 1994, **98**, 11623−11627.

5 Y. Zhao and D. Truhlar, *Theor. Chem. Acc.*, 2008, **120**, 215-241.

6 (a) R. Ditchfield, W. J. Hehre, J. A. Pople, *J. Chem. Phys.* 1971, **54**, 724−728. (b) W. J. Hehre, R. Ditchfield, J. A. Pople, *J. Chem. Phys.* 1972, **56**, 2257−2261. (c) P. C. Hariharan, J. A. Pople, *Theor. Chim. Acta* 1973, **28**, 213−222. (d) J. D. Dill, J. A. Pople, *J. Chem. Phys.* 1975, **62**,

2921−2923. (e) M. M. Francl, W. J. Pietro, W. J. Hehre, J. S. Binkley, M. S. Gordon, D. J. DeFrees, J. A. Pople, *J. Chem. Phys.* 1982, **77**, 3654−3665.

7 (a) S. Grimme, S. Ehrlich, L. Goerigk, *J. Comp. Chem*., 2011, ***32***, 1456; (b) S. Grimme, J. Antony, S. Ehrlich, H. Krieg, *J.Chem.Phys.*, 2010, ***132***, 154104.

[8] (a) C. Peng and H. B. Schlegel, *Israel J. of Chem.,* 1993, ***33***, 449; b) C. Peng, P. Y. Ayala, H. B. Schlegel and M. J. Frisch, *J. Comp. Chem.* 1996*,* ***17***, 49.

[9] (a) K. Fukui, *Acc. Chem. Soc.* 1981, ***12***, 363; (b) C. Gonzalez and H. B. Schlegel, *J. Chem. Phys.* 1989, ***90***, 2154; (c) C. Gonzalez and H. B. Schlegel, *J. Phys. Chem.* 1990, ***94***, 5523.

10 C. Y. Legault, CYLview, 1.0b; Université de Sherbrooke: Sherbrooke, Québec, Canada, 2009; www.cylview.org.
